# Exploring the RC-106 Chemical Space: Design and Synthesis of Novel (*E*)-1-(3-Arylbut-2-en-1-yl)-4-(Substituted) Piperazine Derivatives as Potential Anticancer Agents

**DOI:** 10.3389/fchem.2020.00495

**Published:** 2020-06-30

**Authors:** Roberta Listro, Silvia Stotani, Giacomo Rossino, Marta Rui, Alessio Malacrida, Guido Cavaletti, Michela Cortesi, Chiara Arienti, Anna Tesei, Daniela Rossi, Marcello Di Giacomo, Mariarosaria Miloso, Simona Collina

**Affiliations:** ^1^Medicinal Chemistry and Pharmaceutical Technology Section, Department of Drug Sciences, University of Pavia, Pavia, Italy; ^2^Medicinal Chemistry, Taros Chemicals GmbH and Co. KG, Dortmund, Germany; ^3^Experimental Neurology Unit, School of Medicine and Surgery & Milan Center for Neuroscience, University of Milan Bicocca, Monza, Italy; ^4^Biosciences Laboratory, Istituto Scientifico Romagnolo per lo Studio e la Cura dei Tumori (IRCCS), Meldola, Italy

**Keywords:** cancer, multiple myeloma, glioblastoma, drug discovery, compound library

## Abstract

Despite the fact that significant advances in treatment of common cancers have been achieved over the years, orphan tumors still represent an important unmet medical need. Due to their complex multifactorial origin and limited number of cases, such pathologies often have very limited treatment options and poor prognosis. In the search for new anticancer agents, our group recently identified **RC-106**, a Sigma receptor modulator endowed with proteasome inhibition activity. This compound showed antiproliferative activity toward different cancer cell lines, among them glioblastoma (GB) and multiple myeloma (MM), two currently unmet medical conditions. In this work, we directed our efforts toward the exploration of chemical space around **RC-106** to identify new active compounds potentially useful in cancer treatment. Thanks to a combinatorial approach, we prepared 41 derivatives of the compound and evaluated their cytotoxic potential against MM and GB. Three novel potential anticancer agents have been identified.

## Introduction

Cancer represents one of the leading causes of death worldwide (9.6 million deaths in 2018) (“Cancer” n.d.^1^). Despite the relevant progresses accomplished in the diagnosis and treatment of common cancers, rare tumors are still considered a global issue, in virtue of their negative prognosis (Pillai and Jayasree, 2017). Among the numerous rare cancers listed by the competent organizations, in this work, we focused the attention on glioblastoma (GB) and multiple myeloma (MM), for which effective treatment options are still needed (Shergails et al., 2018; Willenbacher et al., 2018). Glioblastoma is a malignant brain tumor that develops from astrocytes; it is often aggressive and grows into surrounding normal brain tissues (Hambardzumyan and Bergers, 2015). Signs and symptoms of GB are strictly related to the size and location of the tumor (Esmaeili et al., 2018). The availability of only palliative treatments, as well as the risk of relapses, makes GB the most malignant and lethal form of primary brain tumors (Hanif et al., 2017). Multiple myeloma led to abnormal and uncontrolled growth of plasma cells in the bone marrow (Fairfield et al., 2016). Patients in early stages of the pathology have no concerning signs or symptoms, and therefore, the diagnosis is confirmed too late (Rajkumar, 2009). MM is mostly associated with anemia, which exacerbates secondarily to the suppression of erythropoiesis by cytokine networks. Although novel target therapies prolonged from 2.5 to over 10 years the life expectancy, nowadays, a concrete cure is still missing (Banaszkiewicz et al., 2019).

Despite the fact that the molecular basis underlying their pathogenesis has yet to be fully clarified, both cancers share a complex multifactorial origin, where genetic and environmental factors concur in promoting the pathological manifestations (Kanu et al., 2009; Kyrtsonis et al., 2010). Among others, proteasome machinery plays a role in the major degradation of misfolded proteins involved in cancer etiology (Cvek and Dvorak, 2008; Chen Y. et al., 2017). Proteasome inhibitors like Bortezomib (commercially known as Velcade), carfilzomib, and ixazomib (Okazuka and Ishida, 2018) are already used in therapy for the treatment of MM (Chen et al., 2011; Gelman et al., 2013; Ao et al., 2017; Lee et al., 2018). Several other proteasome inhibitors are undergoing clinical trials and testing, including disulfiram (Lövborg et al., 2006), epigallocatechin-3-gallate (Mereles and Hunstein, 2011), Salinosporamide A (Groll et al., 2018), ONX 0912 (Chauhan et al., 2010), CEP-18770 (Sanchez et al., 2010), and MLN9708 (Lee et al., 2011). Recently it has also been demonstrated that Bortezomib is cytotoxic against patient-derived GB cells (Wang et al., 2018) *in vitro* and that it is able to enhance the effect of natural killer significantly reducing tumor volumes in GB-bearing mice (Gras Navarro et al., 2019). Moreover, increasing evidence suggests the involvement of Sigma receptors (SRs) in proteasomal dysfunction and in molecular cascades of cell proliferation and survival (Tesei et al., 2018). Several molecules able to modulate SR-mediated pathways are able to inhibit growth, migration, and invasion of cancer cells (Aydar et al., 2006; Collina et al., 2017). A high density of SRs has been found in numerous cancer cell lines, including Roswell Park Memorial Institute (RPMI) 8226, a human MM cell line, and U87MG, a glioblastoma cell line (Brune et al., 2012). Moreover, GB malignancy and aggressiveness are strictly related to the expression level of SRs (Kranz et al., 2018; Liu et al., 2019).

**Figure 1:**
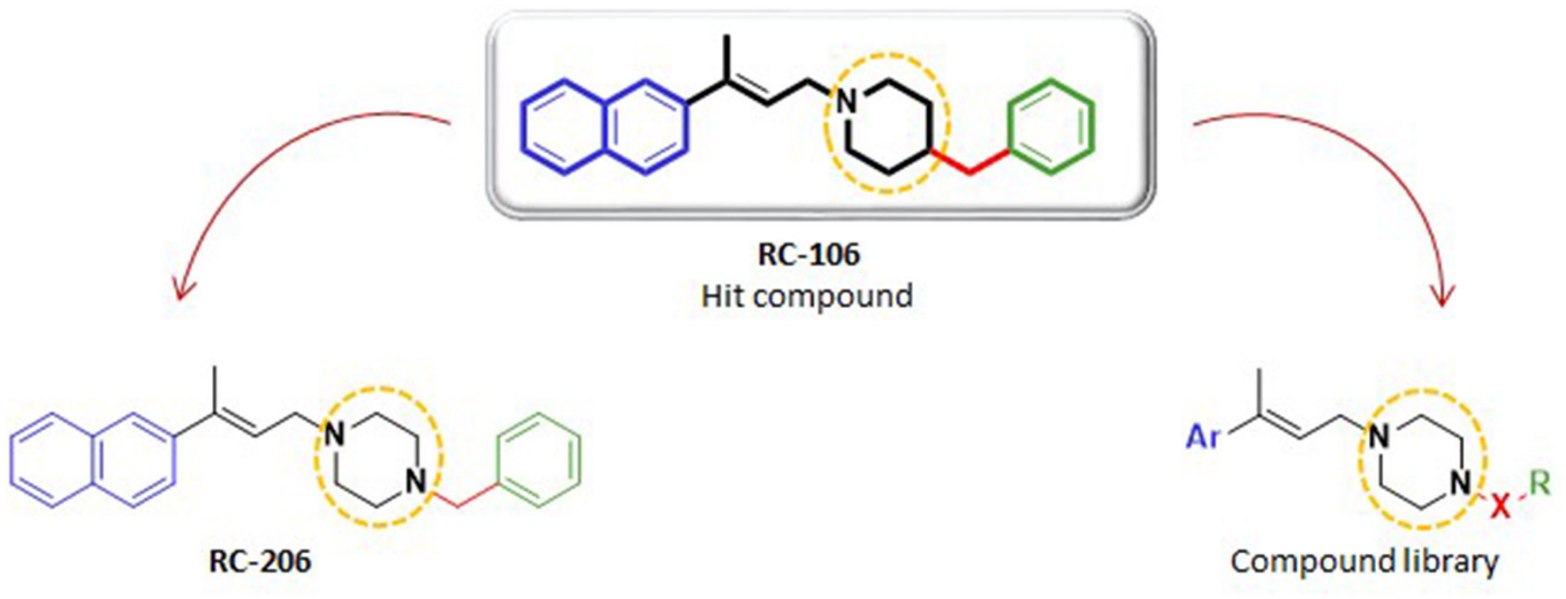
Structures of *hit* compound **RC-106**, its analog **RC-206**, and common scaffold of the new compound library.

Our research team has been active in this field, studying potential anticancer compounds acting through proteasome complex inhibition and SR modulation (Collina et al., 2013; Rui et al., 2016a; Rossi et al., 2017; Malacrida et al., 2019). Our medicinal chemistry campaign recently led to the identification of (*E*)-4-benzyl-1-[3-(naphthalen-2-yl)but-2-en-1-yl]piperidine (henceforth **RC-106**) (Figure 1). This compound is able to activate terminal unfolded protein response (UPR) and to inhibit proteasome complex activity, through the induction of endoplasmic reticulum stress (ER) (Tesei et al., 2019). Moreover, **RC-106** possesses a pan-SRs profile—ability to bind both S1R and S2R—and shows a cytotoxic effect against a wide panel of cancer cell lines, all expressing SRs, acting as a proapoptotic drug, which induces a fast triggering of cell death program (Rui et al., 2016b). Despite the promising antitumor profile of **RC-106**, this compound still suffers from some drawbacks, namely, solubility issues, which might interfere significantly with further investigations and development. Accordingly, we planned to develop a series of analogs in the search for molecules with comparable or enhanced *in vitro* efficacy and improved pharmacokinetic properties.

Herein, we report the results of a study broadening the structural diversity of the *hit* **RC-106**. We replaced the piperidine ring with a piperazine moiety, speculating that this structural elaboration might not affect the anticancer profile. To validate our hypothesis, we first synthesized the piperazine analog named **RC-206**, and then we built and synthesized *via* a combinatorial approach a compound library of 40 molecules. The designed members were *in silico* studied to evaluate their drug-likeness (employing the swissADME free web tool Daina et al., 2017), and their synthetic feasibility was evaluated. Lastly, their cytotoxic profile against U87 and RPMI 8226 cell lines, representative of GB and MM, respectively, was evaluated.

## Materials and Methods

### Chemistry

#### General Remarks

Chemicals and solvents were obtained from commercial suppliers and were used without further purification. All dry reactions were performed under nitrogen atmosphere using commercial dry solvents. For Fourier-transform infrared spectroscopy (FTIR) analysis, a Spectrum One Perkin Elmer spectrophotometer equipped with a MIRacle™ ATR device was used. The IR spectra were scanned over a wavenumber range of 4,000–650 cm^−1^ with a resolution of 4 cm^−1^. Analytical thin-layer chromatography (TLC) was carried out on silica-gel-precoated glassbacked plates (Fluka Kieselgel 60 F254, Merck), visualized by ultraviolet (UV) radiation, acidic ammonium molybdate (IV), or potassium permanganate. Flash chromatography (FC) was performed with Silica Gel 60 (particle size, 230–400 mesh, purchased from Sigma Aldrich) or Grace Reveleris X2 flash chromatography system using silica-gel-packed Macherey Nagel Chromabond Flash BT cartridges (60 Å, 45 μm) and Grace Reveleris flash Cartridges (60 Å, 40 μm). Proton nuclear magnetic resonance (NMR) spectra were recorded on Bruker Avance 400 and 300 spectrometers operating at 400 and 300 MHz, respectively. Proton chemical shifts (δ) are reported in parts per million with the solvent reference relative to tetramethylsilane (TMS) internal standard (CDCl_3_, δ = 7.26 ppm). The following abbreviations are used to describe spin multiplicity: s, singlet; d, doublet; t, triplet; q, quartet; m, multiplet; br, broad signal; dd, doublet–doublet; td, triplet–doublet. The coupling constant values are reported in Hertz. ^13^C NMR spectra were recorded on 400 and 300 MHz spectrometers operating at 100 MHz, with complete proton decoupling. Carbon chemical shifts (δ) are reported in parts per million relative to TMS with the respective solvent resonance as the internal standard (CDCl_3_, δ = 77.23 ppm). Ultraperformance liquid chromatography-UV-electron spray ionization/mass spectrometry (UPLC-UV-ESI/MS) analyses were carried out on an Acuity UPLC Waters LCQ FLEET system using an ESI source operating in positive ion mode, controlled by Acquidity PDA and 4 MICRO (Waters). Analyses were run on a Acquity BEH Phenyl (ABP) (50 × 2.1, 1.7 mm) or Acquity BEH Shield (ABS) (100 × 2.1, 1.7 mm) columns, at room temperature, with gradient elution (solvent A: water containing 0.1% of formic acid; solvent B: methanol containing 0.1% of formic acid; gradient: 10% B in A to 100% B in 3 min, followed by isocratic elution 100% B for 1.5 min, return to the initial conditions in 0.2 min) at a flowrate of 0.5 ml min^−1^. All the final compounds had 95% or greater purity.

Analytical, preparative high-performance liquid chromatography (HPLC) and ESI condition mass spectra were performed on an Agilent UHPLC (1290 Infinity) and an Agilent Prep-HPLC (1260 Infinity) both equipped with a diode array detector and a quadrupole MS Dusing mixture gradient of formic acid/water/acetonitrile as system solvent. High-resolution ESI Fourier transform mass spectrometry (ESI-FTMS) mass spectra were recorded on a Thermo LTQ Orbitrap (high-resolution mass spectrometer from Thermo Electron) coupled to an “Accela” HPLC system supplied with a “Hypersil GOLD” column (Thermo Electron).

#### General Procedure for the Synthesis of Allylic Esters 1[1–4]

In a two-neck round-bottom flask, preserving the anhydrous conditions, solid reagents are added quickly. After AcONa (2 equiv.) addition, Palladium EnCat® (loading, 0.4 mmol/g, 1%, 0.01 equiv.) is added with TEAC (2 equiv.). Afterwards, the liquid reagent (*E*)-ethyl but-2-enoate (1.5 equiv.; *d* = 0.918 g/ml) and the dry solvent dimethylformamide (DMF) are added, too. Separately, in a round-bottom flask, a solution of aryl bromide (Ar-Br, 1 equiv.) in dry DMF is prepared. This solution is then transferred to a dropping funnel, preventively added to the second neck of the reaction flask. The total volume of dry DMF used within these two steps has to provide a final concentration in the reaction ambient close to 0.35 M. This reaction mixture, kept in inert medium and under magnetic stirring, is heated under reflux with an oil bath. At the beginning, the temperature is settled at 50°C. During the addition of the aryl bromide solution, that should last for 1 h, the temperature is gradually increased until it reaches 75°C. Once the additions are finished, the heating is increased again until reaching a temperature of 105°C. After almost 2 h from the beginning of the aryl bromide addition, the reaction mixture starts getting darker, and after one more hour, it gets totally black. This color change means that the reaction has gone to completion, as confirmed by TLC with MP Hex/AcOEt 95:5 where the aryl bromide spot disappears. The raw product is then filtered on a paper filter and simultaneously transferred in a separating funnel. The reaction mixture is diluted and extracted three times with Et_2_O. The collected organic phases are washed with water, dried with Na_2_SO_4_ anhydrous, filtrated, and evaporated to dryness at reduced pressure. The dark-brown raw oil obtained after solvent evaporation is purified by chromatography on silica gel (see below for more detail). The purified intermediate products were characterized by ^1^H and ^13^C NMR.

*(E)-Ethyl 3-phenylbut-2-enoate* **1**[1]: by following the general procedure, starting from bromobenzene (4.9 g), the desired product was obtained as clear oil (2.8 g). Yield: 46.7%. *R*_f_: 0.5 (TLC Hex/AcOEt, 95:5 *v*/*v*). Purification: flash chromatography, gradient elution, MP Hex/AcOEt 99:1, 97:3, and 96:4 *v*/*v*, 5 cm diameter column. IR (cm^−1^): 601.682; 628.68; 692.32; 724.139; 734.746; 764.637; 870.703; 1,041.37; 1,094.4; 1,155.15; 1,270.86; 1,343.18; 1,365.35; 1,445.39; 1,493.6; 1,539.88; 1,576.52; 1,626.66; 1,708.62; 2,116.49; 2,301.63; 2,359.48; 2,959.23; 3,024.8. ^1^H NMR (400 MHz, CDCl_3_): δ 7.50 (d, *J* = 5.1 Hz, 2H), 7.44–7.33 (m, 3H), 6.16 (s, 1H), 4.24 (q, *J* = 7.1 Hz, 2H), 2.60 (s, 3H), 1.34 (t, *J* = 7.1 Hz, 3H). ^13^C NMR (101 MHz, CDCl_3_): δ 166.77, 155.41, 142.11, 128.86, 128.37, 126.19, 117.06, 59.73, 17.83, 14.23.

*(E)-Ethyl 3-(4-methoxyphenyl)but-2-enoate* **1**[2]: by following the general procedure, starting from 1-bromo-4-methoxybenzene (4.5 g), the desired product was obtained as a clear oil (2.6 g). Yield: 49%. *R*_f_: 0.35 (TLC Hex/AcOEt, 95:5 *v*/*v*). Purification: flash chromatography, gradient elution, MP Hex/AcOEt 98:2, 97:3, and 96:4 *v*/*v*, 5 cm diameter column. IR (cm^−1^): 627.716; 697.141; 735.71; 828.277; 870.703; 1,030.77; 1,152.26; 1,249.65; 1,273.75; 1,343.18; 1,365.35; 1,440.56; 1,511.92; 1,573.63; 1,601.59; 1,624.73; 1.705.73; 2,143.49; 2,338.27; 2,836.77; 2,901.38; 2,988.16. ^1^H NMR (400 MHz, CDCl_3_): δ 7.47 (d, *J* = 8.8 Hz, 2H), 6.91 (d, *J* = 8.8 Hz, 2H), 6.13 (d, *J* = 0.7 Hz, 1H), 4.22 (q, *J* = 7.1 Hz, 2H), 3.84 (s, 3H), 2.58 (d, *J* = 0.5 Hz, 3H), 1.33 (t, *J* = 7.1 Hz, 3H). ^13^C NMR (101 MHz, CDCl_3_): δ 166.98, 160.28, 154.78, 134.15, 127.54, 115.15, 113.67, 59.61, 55.20, 17.52, 14.25.

*(E)-Ethyl 3-(naphthalen-2-yl)but-2-enoate* **1**[3]: by following the general procedure, starting from 2-bromonaphtalene (4.2 g), the desired product was obtained as a white solid (2.4 g). Yield: 49.3%. *R*_f_: 0.44 (TLC Hex/AcOEt, 95:5 *v*/*v*). mp: 54°C. Purification: flash chromatography, gradient elution, MP Hex/AcOEt 97:3 and 96:4 *v*/*v*, 5 cm diameter column. IR (cm^−1^): 601.682; 627.716; 665.321; 707.747; 724.139; 735.71; 816.706; 1,129.12; 1,413.57; 1,540.85; 1,704.76; 2,143.49; 2,286.2; 2,337.3; 2,391.3; 2,847.38; 2,911.99; 2,953.45; 2,997.8. ^1^H NMR (400 MHz, CDCl_3_): δ 7.98 (s, 1H), 7.92–7.81 (m, 3H), 7.63 (dd, *J* = 8.6, 1.7 Hz, 1H), 7.57–7.49 (m, 2H), 6.33 (d, *J* = 0.9 Hz, 1H), 4.28 (q, *J* = 7.1 Hz, 2H), 2.73 (d, *J* = 0.9 Hz, 3H), 1.37 (t, *J* = 7.1 Hz, 3H). ^13^C NMR (101 MHz, CDCl_3_): δ 167.39, 155.70, 139.53, 133.93, 133.30, 128.71, 128.45, 127.77, 126.92, 126.80, 126.24, 124.39, 118.01, 60.20, 17.95, 14.69.

*(E)-Ethyl 3-(6-methoxynaphthalen-2-yl)but-2-enoate* **1**[4]: by following the general procedure, starting from 2-bromo-6-methoxynaphthalene (6.6 g), the desired product was obtained as a white solid (2.9 g). Yield: 50%. *R*_f_: 0.3 (TLC Hex/AcOEt, 95:5 *v*/*v*). mp: 78°C. Purification: flash chromatography, MP Hex/AcOEt 9:1 *v*/*v*, 5 cm diameter column. IR (cm^−1^): 601.682; 618.074; 627.716; 665.321; 706.783; 735.71; 815.742; 850.454; 1,157.08; 1,424.17; 1,540.85; 1,565.92, 1,647.88, 1,747.19, 2,217.74; 2,995.87; 3,613.95. ^1^H NMR (400 MHz, CDCl_3_): δ 7.91 (s, 1H), 7.76 (dd, *J* = 15.0, 8.8 Hz, 2H), 7.61 (d, *J* = 8.6 Hz, 1H), 7.22–7.16 (m, 1H), 7.15 (d, *J* = 1.3 Hz, 1H), 6.30 (s, 1H), 4.27 (q, *J* = 7.1 Hz, 2H), 3.95 (s, 3H), 2.71 (s, 3H), 1.36 (t, *J* = 7.1 Hz, 3H). ^13^C NMR (101 MHz, CDCl_3_): δ 166.89, 158.26, 155.14, 136.92, 134.72, 129.92, 128.43, 126.81, 125.67, 124.32, 119.22, 116.53, 105.46, 59.69, 55.20, 17.64, 14.26.

#### General Procedure for the Reduction to Allylic Alcohols 2[1–4]

In a one-neck round-bottom flask, preserving the anhydrous conditions, the allylic ester (1 equiv.) from the previous synthetic step is solubilized in dry Et_2_O under magnetic stirring. Once the solution is homogeneous, the reaction flask is put in a 0°C ice bath. Afterwards, LiAlH_4_ [1 equiv., 1 M solution in dry tetrahydrofuran (THF)] is added, slowly and dropwise, to the reaction mixture. After almost 30 min from the end of the reducing agent addition, the reaction is monitored by TLC with MP Hex/AcOEt 7:3 and results completed. The reaction is then quenched by adding a few drops of AcOEt and, afterwards, some of NH_4_Cl saturated aqueous solution. The workup procedure follows three extractions with Et_2_O and washing of the collected organic phases with brine. After that, the organic phase is dried with anhydrous Na_2_SO_4_, filtrated, and evaporated to dryness at reduced pressure. Depending on the purity of the raw product, as reported below, this is either purified by chromatography on silica gel or used directly in the subsequent reaction. The products were characterized by ^1^H and ^13^C NMR.

*(E)-3-Phenylbut-2-en-1-ol* **2**[1]: by following the general procedure, starting from compound **1**[1] (0.8 g), the desired product was obtained as a clear oil (0.617 g). Yield: 95.4%. *R*_f_: 0.375 (TLC Hex/AcOEt, 6:4 *v*/*v*). Purification: flash chromatography, gradient elution, MP Hex/AcOEt 95:5, 7:3, and 6:4 *v*/*v*, 5 cm diameter column. IR (cm^−1^): 601.682; 609.396; 627.716; 698.105; 735.71; 758.852; 871.667; 1,026.91; 1,061.62; 1,149.37; 1,375.96; 1,445.39; 1,475.28; 1,493.6; 1,540.85; 1,648.84; 1,703.8; 1,722.12; 2,062.5; 2,337.3; 2,888.84; 2,985.27; 3,310.21. ^1^H NMR (400 MHz, CDCl_3_): δ 7.44 (d, *J* = 6.8 Hz, 2H), 7.36 (t, *J* = 6.8 Hz, 2H), 7.29 (m, 1H), 6.00 (t, *J* = 6.4 Hz, 1H), 4.39 (d, *J* = 6.4 Hz, 2H), 2.11 (s, 3H), 1.76 (s, 1H). ^13^C NMR (101 MHz, CDCl_3_): δ 142.73, 137.71, 128.18, 127.18, 126.38, 125.67, 59.83, 15.92.

*(E)-3-(4-Methoxyphenyl)but-2-en-1-ol* **2**[2]: by following the general procedure, starting from compound **1**[2] (1.17 g), the desired product was obtained as a white solid (0.95 g). Yield: ≥99.9%. *R*_f_: 0.3 (TLC Hex/AcOEt, 6:4 *v*/*v*). mp: 96°C. Purification: none; after the NMR analysis, the crude product obtained from the reaction workup displayed a suitable purity for the following step. IR (cm^−1^): 601.682; 627.716; 665.321; 706.783; 735.71; 798.385; 1,025.94; 1,180.22; 1,247.72; 1,285.32; 1,440.56; 1,509.99; 1,605.45; 2,338.27; 2,871.49; 2,952.48; 2,999.73; 3,244.65. ^1^H NMR (400 MHz, CDCl_3_): δ 7.38 (d, *J* = 8.7 Hz, 2H), 6.89 (d, *J* = 8.7 Hz, 2H), 5.94 (t, *J* = 6.7 Hz, 1H), 4.37 (d, *J* = 6.7 Hz, 2H), 3.83 (s, 3H), 2.08 (s, 3H), 1.50 (s, 1H). ^13^C NMR (101 MHz, CDCl_3_): δ 158.88, 137.27, 135.18, 126.71, 124.70, 113.49, 59.83, 55.17, 15.91, 15.79.

*(E)-3-(Naphthalen-2-yl)but-2-en-1-ol* **2**[3]: by following the general procedure, starting from compound **1**[3] (2.00 g), the desired product was obtained as a white solid (1.35 g). Yield: 81.8%. *R*_f_: 0.36 (TLC Hex/AcOEt, 6:4 *v*/*v*). mp: 63°C. Purification: flash chromatography, gradient elution, MP Hex/AcOEt 7:3 and 4:6 *v*/*v*, 5 cm diameter column. IR (cm^−1^): 609.396; 627.716; 665.321; 724.139; 738.603; 814.777; 858.168; 893.844; 1,008.59; 1,099.23; 1,375; 1,506.13; 1,540.85; 1,596.77; 1,670.05; 1,705.73; 1,747.19, 2,217.74, 2,372.01; 2,985.27; 3,339.14. ^1^H NMR (400 MHz, CDCl_3_): δ 7.94–7.78 (m, 4H), 7.70–7.58 (m, 1H), 7.55–7.43 (m, 2H), 6.17 (t, *J* = 6.6 Hz, 1H), 4.46 (d, *J* = 6.6 Hz, 2H), 2.22 (s, 3H), 1.60 (s, 1H). ^13^C NMR (101 MHz, CDCl_3_): δ 139.83, 137.51, 133.24, 132.58, 128.02, 127.68, 127.40, 126.91, 126.00, 125.71, 124.39, 124.03, 59.95, 15.93.

*(E)-3-(6-Methoxynaphthalen-2-yl)but-2-en-1-ol* **2**[4]: by following the general procedure, starting from compound **1**[4] (1.11 g), the desired product was obtained as a white solid (0.8 g). Yield: 84.7%. *R*_f_: 0.28 (TLC Hex/AcOEt, 6:4 *v*/*v*). mp: 105°C. Purification: none; after the NMR analysis, the raw product obtained from the reaction workup results adequately pure to move forward with the following step. IR (cm^−1^): 602.646; 627.716; 665.321; 735.71; 809.956; 1,028.84; 1,164.79; 1,540.85; 1,646.91; 1,705.73; 1,747.19; 2,217.74; 2,996.84; 3,208. ^1^H NMR (400 MHz, CDCl_3_): δ 7.75 (m, 3H), 7.60 (d, *J* = 8.8 Hz, 1H), 7.15 (m, 1H), 6.14 (t, *J* = 6.5 Hz, 1H), 4.44 (d, *J* = 6.5 Hz, 2H), 3.95 (s, 3H), 2.20 (s, 3H), 1.55 (s, 1H). ^13^C NMR (101 MHz, CDCl_3_): δ 197.91, 157.56, 137.64, 137.60, 133.72, 129.54, 128.64, 126.53, 124.49, 124.24, 118.85, 105.42, 59.90, 55.20, 15.89.

#### General Procedure for the Synthesis of Allylic Amines 3[1–4]

In a one-neck round-bottom flask, a mixture of allylic alcohol (1 equiv.) and PPh_3_ (1.5 equiv.) is solubilized in dry THF (2/3 of the total volume calculated in order to have a final concentration of ~0.5 M) under magnetic stirring and preserving the anhydrous conditions. Separately, a Dewar containing ice, NaCl, and MeOH is set up to reach the temperature of −18°C. Once the solution is homogeneously stirred, the flask is cooled in the ice bath. Afterwards, the reaction mixture is treated with *N*-bromosuccinimide (NBS) (1.4 equiv.): this operation should be done carefully, adding the NBS portion-wise in six equal fractions each 5–10 min. During this step, it is extremely important to pay attention to the NBS solubilization: these white crystals tend to precipitate as a yellow solid or form a yellow oil phase above the reaction mixture. Therefore, for each two additions, it is useful to bring out the flask from the ice bath and let it heat to room temperature, improving the NBS solubilization. Nevertheless, every addition must be done at −18°C. Once the entire amount of NBS is added, the reaction flask is allowed to warm to room temperature and stirred for 20–30 min. A color variation from a clear solution (in some case lightly yellow) to a brownish suspension (more or less dark, depending on the substrate) is observed. Monitoring the reaction by TLC with MP Hex/AcOEt 7:3, the alcohol spot slowly disappears in favor of the one representing the hypothetical alcohol-PPh_3_-NBS abduct. Meanwhile, in another anhydrous round-bottom flask, the amines 1-Boc-piperazine (1.2 equiv.) and Et_3_N (2 equiv.) are solubilized in the residual part of dry THF (1/3 of the total volume calculated in order to have a final concentration of almost 0.5 M). This solution is later added to the reaction flask, previously cooled again to −18°C. After the addition of these last reagents, the reaction flask is brought out from the ice bath one more time and allowed to react overnight at room temperature under magnetic stirring. The day after, the reaction is monitored by TLC with MP AcOEt/Hex 8:2, and then, the TLC plate is developed with the stain reagent ninhydrin to confirm the presence of the amine and the absence of the alcohol-PPh_3_-NBS abduct previously observed. The raw product is worked up by dilution with Et_2_O, filtration directly into the separating funnel, and three times washing with a Na_2_CO_3_ saturated aqueous solution. The washed organic phase is then dried with Na_2_SO_4_ anhydrous, filtrated, and evaporated to dryness at reduce pressure. This way, a raw dark oil to be purified by chromatography on silica gel is obtained. The purified key intermediates were characterized by ^1^H and ^13^C NMR.

*(E)-Tert-butyl 4-(3-phenylbut-2-en-1-yl)piperazine-1-carboxylate* **3**[1]: by following the general procedure, starting from compound **2**[1] (0.60 g), the desired product was obtained as a red-brown oil (1.0 g). Yield: 78.9%. Overall yield: 35.2%. *R*_f_: 0.41 (TLC AcOEt/Hex, 7:3 *v*/*v*). Purification: flash chromatography, MP Hex/AcOEt 3:7 *v*/*v*, 5 cm diameter column. IR (cm^−1^): 601.682; 627.716; 696.177; 755.959; 865.882; 914.093; 1,001.84; 1,122.37; 1,169.62; 1,244.83; 1,287.25; 1,364.39; 1,417.42; 1,693.19; 2,217.74; 2,764.46; 2,843.52; 2,981.41. ^1^H NMR (400 MHz, CDCl_3_): δ 7.41 (d, *J* = 7.6 Hz, 2H), 7.33 (t, *J* = 7.6 Hz, 2H), 7.26 (m, 1H), 5.89 (t, *J* = 6.8 Hz, 1H), 3.51–3.45 (m, 4H), 3.21 (d, *J* = 6.8 Hz, 2H), 2.55–2.42 (m, 4H), 2.08 (s, 3H), 1.47 (s, 9H). ^13^C NMR (101 MHz, CDCl_3_): δ 154.60, 143.05, 138.02, 128.11, 126.94, 125.55, 123.85, 79.49, 56.55, 52.89, 28.30, 16.09.

*(E)-Tert-butyl 4-(3-(4-methoxyphenyl)but-2-en-1-yl) piperazine-1-carboxylate* **3**[2]: by following the general procedure, starting from compound **2**[2] (0.90 g), the desired product was obtained as a yellow-orange oil (1.0 g). Yield: 57.2%. Overall yield: 28%. *R*_f_: 0.34 (TLC AcOEt/Hex, 7:3 *v*/*v*). Purification: flash chromatography, MP Hex/AcOEt 3:7 *v*/*v*, 5 cm diameter column. IR (cm^−1^): 627.716; 670.142; 735.71; 826.348; 864.917; 916.986; 999.91; 1,032.69; 1,119.48; 1,170.58; 1,243.86; 1,287.25; 1,364.39; 1,417.42; 1,511.92; 1,607.38; 1,692.23; 2,340.19; 2,361.41; 2,388.41; 2,814.6; 2,871.49; 2,983.34. ^1^H NMR (400 MHz, CDCl_3_): δ 7.36 (d, *J* = 8.7 Hz, 2H), 6.87 (d, *J* = 8.7 Hz, 2H), 5.82 (t, *J* = 6.8 Hz, 1H), 3.82 (s, 3H), 3.48 (brs, 4H), 3.19 (d, *J* = 6.8 Hz, 2H), 2.48 (brs, 4H), 2.05 (s, 3H), 1.47 (s, 9H). ^13^C NMR (101 MHz, CDCl_3_): δ 177.45, 158.66, 154.64, 137.32, 135.51, 128.84, 126.59, 122.19, 113.44, 79.54, 56.57, 55.14, 52.87, 29.51, 28.31, 16.09.

*(E)-Tert-butyl 4-(3-(naphthalen-2-yl)but-2-en-1-yl) piperazine-1-carboxylate* **3**[3]: by following the general procedure, starting from compound **2**[3] (0.81 g), the desired product was obtained as a white-yellow solid (1.16 g). Yield: 77.7%. Overall yield: 31.6%. *R*_f_: 0.37 (TLC AcOEt/Hex, 7:3 *v*/*v*). mp: 93°C. Purification: flash chromatography, MP Hex/AcOEt 4:6 *v*/*v*, 5 cm diameter column. IR (cm^−1^): 601.682; 627.716; 665.321; 735.71; 816.706; 1,094.4; 1,424.17; 1,540.85; 1,647.88; 1,705.73; 1,747.19, 2,217.74, 2,372.01; 2,912.95; 2,991.05. ^1^H NMR (400 MHz, CDCl_3_): δ 7.83 (m, 4H), 7.61 (d, *J* = 8.6 Hz, 1H), 7.54–7.40 (m, 2H), 6.07 (t, *J* = 6.7 Hz, 1H), 3.52 (brs, 4H), 3.29 (d, *J* = 6.7 Hz, 2H), 2.54 (brs, 4H), 2.20 (s, 3H), 1.49 (s, 9H). ^13^C NMR (101 MHz, CDCl_3_): δ 154.60, 140.13, 133.27, 132.50, 127.97, 127.62, 127.37, 126.01, 125.59, 124.25, 124.16, 124.06, 79.57, 56.65, 52.92, 29.57, 28.22, 16.12.

*(E)-Tert-butyl 4-(3-(6-methoxynaphthalen-2-yl)but-2-en-1-yl)piperazine-1-carboxylate* **3**[4]: by following the general procedure, starting from compound **2**[4] (0.80 g), the desired product was obtained as a white-yellow solid (1.1 g). Yield: 79.2%. Overall yield: 38.1%. mp: 133°C. *R*_f_: 0.34 (TLC AcOEt/Hex, 7:3 *v*/*v*). Purification: flash chromatography, MP Hex/AcOEt 3:7 *v*/*v*, 5 cm diameter column. IR (cm^−1^): 601.682; 617.109; 627.716; 665.321; 706.783; 724.139; 7735.71; 815.742; 1,114.65; 1,397.17; 1,565.92; 1,646.91; 1,745.26; 2,284.27; 2,389.37; 2,996.84. ^1^H NMR (400 MHz, CDCl_3_): δ 7.84–7.66 (m, 3H), 7.58 (d, *J* = 8.6 Hz, 1H), 7.21–7.08 (m, 2H), 6.03 (t, *J* = 6.7 Hz, 1H), 3.93 (s, 3H), 3.51 (brs, 4H), 3.26 (d, *J* = 6.7 Hz, 2H), 2.52 (brs, 4H), 2.17 (s, 3H), 1.49 (s, 9H). ^13^C NMR (101 MHz, CDCl_3_): δ 157.49, 154.63, 138.03, 137.70, 133.60, 129.47, 128.70, 126.47, 124.53, 123.97, 123.73, 118.78, 105.45, 79.50, 56.70, 55.17, 52.97, 28.35, 16.07.

#### General Procedure for the de-boc Reactions

Into a two-necked round bottomed flask of the appropriate volume, the Boc-protected intermediate was dissolved in 10 ml of 1,4-dioxane. The mixture was cooled to 0°C using an ice bath and 10 ml of 4 N HCl in dioxane were added dropwise. The mixture was allowed to reach room temperature and stirred at such temperature overnight. Solvent was evaporated, and the resulting de-Boc products (as HCl salts) were used without further purification.

*1-[(2E)-3-Phenylbut-2-en-1-yl]piperazine* **4**[1], orange oil, 88%, *R*_f_ = 0.55 (CHCl_3_/MeOH 5:1), UHPLC-ESI-MS: *R*_t_ = 1.34, *m*/*z* = 217.3 [M + H]^+^. ^1^H NMR (300 MHz, CDCl_3_), δ 7.42–7.38 (m, 2H), 7.34–7.28 (m, 3H), 5.90 (dt, *J* = 1.3 Hz, *J* = 6.8 Hz, 1H), 3.18 (dd, *J* = 0.7 Hz, *J* = 6.8 Hz, 2H), 2.94 (t, *J* = 4.9 Hz, 4H), 2.52 (s, 4H), 2.07 (s, 3H) ppm; ^13^C NMR (100 MHz, CDCl_3_), δ 143.3, 137.7, 128.2, 127.0, 125.7, 124.4, 57.3, 54.4, 46.0, 16.2 ppm.

*1-[(2E)-3-(4-Methoxyphenyl) but-2-en-1-yl]piperazine* **4**[2], orange oil, 87%, *R*_f_ = 0.62 (CHCl_3_/MeOH 5:1), UHPLC-ESI-MS: *R*_t_ = 1.71, *m*/*z* = 247.3 [M + H]^+^. ^1^H NMR (300 MHz, CDCl_3_), δ 7.35 (d, *J* = 8.7 Hz, 2H), 6.85 (d, *J* = 8.7 Hz, 2H), 5.82 (dt, *J* = 1.0 Hz, *J* = 6.9 Hz, 1H), 3.81 (s, 3H), 3.17 (d, *J* = 6.5 Hz, 2H), 2.95–2.92 (m, 4H), 2.51 (s, 4H), 2.04 (s, 3H) ppm; ^13^C NMR (100 MHz, CDCl_3_), δ 158.7, 137.1, 135.8, 126.7, 122.7, 113.6, 57.3, 55.3, 54.4, 46.0, 16.2 ppm.

*1-[(2E)-3-(Naphthalen-2-yl)but-2-en-1-yl]piperazine* **4**[3], orange solid, 93%, *R*_f_ = 0.65 (CHCl_3_/MeOH 5:1), UHPLC-ESI-MS: *R*_t_ = 1.91, *m*/*z* = 267.2 [M + H]^+^. ^1^H NMR (300 MHz, CDCl_3_), δ 7.81–7.77 (m, 4H), 7.60 (d, *J* = 8.3 Hz, 1H), 7.48–7.41 (m, 2H), 6.05 (t, *J* = 7.1 Hz, 1H), 3.25 (d, *J* = 6.7 Hz, 2H), 3.00 (s, 4H), 2.59 (s, 4H), 2.18 (s, 3H) ppm; ^13^C NMR (100 MHz, CDCl_3_), δ 140.3, 137.8, 133.4, 132.6, 128.1, 127.7, 127.5, 126.1, 125.7, 124.8, 124.2, 124.2, 57.2, 53.7, 45.6, 16.2 ppm.

*1-[(2E)-3-(6-Methoxynaphthalen-2-yl)but-2-en-1-yl]piperazine* **4**[4], white solid, 80%, *R*_f_ = 0.68 (CHCl_3_/MeOH 5:1), UHPLC-ESI-MS: *R*_t_ = 1.99, *m*/*z* = 297.2 [M + H] ^+^. ^1^H NMR (300 MHz, CDCl_3_), δ 7.75–7.66 (m, 4H), 7.57 (dd, *J* = 1.9 Hz, *J* = 8.6 Hz, 1H), 7.15–7.11 (m, 2H), 6.02 (dt, *J* = 1.4 Hz, *J* = 6.9 Hz 1H), 3.92 (s, 3H), 3.25 (d, *J* = 6.6 Hz, 2H), 2.98 (s, 4H), 2.58 (s, 4H), 2.16 (s, 3H) ppm; ^13^C NMR (100 MHz, CDCl_3_), δ 159.0, 142.5, 139.8, 137.9, 132.2, 128.8, 127.4, 126.6, 124.4, 121.8, 119.1, 105.6, 54.9, 54.2, 50.7, 43.1, 16.2 ppm.

#### General Procedure for Sulfonylation

Reactions were performed in parallel in 15-ml reaction tubes in a 24-position Mettler-Toledo Miniblock® equipped with a heat transfer block and inert gas manifold. Each reaction tube was loaded with a previously prepared solution of 30 mg of the corresponding amine (1.0 equiv.) in 2 ml of DCM and TEA (5.0 equiv.). The corresponding sulfonyl chlorides (1.5 equiv.) were added. The reaction mixtures were stirred at room temperature overnight. Reaction conversion was confirmed through UHPLC check of some representative samples. The mixtures were evaporated until dryness. The crudes were redissolved in 1.0 ml of acetonitrile (ACN), filtered and purified with preparative HPLC (gradient acetonitrile/water with 0.1% formic acid, 2–98%). Fractions containing pure product were combined and evaporated to dryness in Mettler vials.

*(E)-1-(Cyclopentylsulfonyl)-4-(3-phenylbut-2-en-1-yl) piperazine* **SU**[1,1], yellow oil, 75%, UHPLC-ESI-MS: *R*_t_ = 2.21, *m*/*z* = 349.2 [M + H]^+^. Purity (UHPLC) = 97%

*(E)-1-(Cyclohexylsulfonyl)-4-(3-phenylbut-2-en-1-yl) piperazine* **SU**[1,2], white solid, 52%, UHPLC-ESI-MS: *R*_t_ = 2.40, *m*/*z* = 363.2 [M + H]^+^. Purity (UHPLC) = 96%

*1-[(2E)-3-Phenylbut-2-en-1-yl]-4-[4-(trifluoromethyl) benzenesulfonyl]piperazine* **SU**[1,3], white solid, 37%, *R*_f_ = 0.90 (DCM/MeOH 19:1), UHPLC-ESI-MS: *R*_t_ = 2.46, *m*/*z* = 425.2 [M + H]^+^. Purity (UHPLC) = 99%. ^1^H NMR (300 MHz, CDCl_3_), δ 7.88 (d, *J* = 8.4 Hz, 1H), 7.83–7.80 (m, 2H), 7.57 (d, *J* = 8.2 Hz, 1H), 7.34–7.28 (m, 5H), 5.80 (dt, *J* = 0.9 Hz, *J* = 7.2 Hz, 1H), 3.61 (s, 3H), 3.39 (s, 4H), 3.06 (s, 4H), 2.09 (s, 3H) ppm; ^13^C NMR (100 MHz, CDCl_3_), δ 142.0, 139.2, 135.2, 134.7, 128.4, 128.2, 127.8, 126.4, 126.3, 125.7, 125.4, 55.9, 51.7, 44.8, 16.4 ppm.

*1-(4-Methylbenzenesulfonyl)-4-[(2E)-3-phenylbut-2-en-1-yl] piperazine* **SU**[1,4], yellow oil, 31%, *R*_f_ = 0.66 (DCM/MeOH 19:1), UHPLC-ESI-MS: *R*_t_ = 2.30, *m*/*z* = 371.0 [M + H]^+^. Purity (UHPLC) = 89%. ^1^H NMR (300 MHz, CDCl_3_), δ 7.63–7.59 (m, 3H), 7.34–7.28 (m, 5H), 7.10 (d, *J* = 8.0 Hz, 1H), 5.80 (dt, *J* = 1.0 Hz, *J* = 7.3 Hz, 1H), 3.57 (s, 2H), 3.27 (s, 4H), 2.98 (s, 4H), 2.43 (s, 3H), 2.07 (s, 3H) ppm; ^13^C NMR (100 MHz, CDCl_3_), δ 144.3, 141.8, 140.1, 132.2, 129.9, 128.8, 128.3, 127.7, 125.8, 125.7, 55.7, 51.4, 44.5, 21.5, 16.4 ppm.

*1-(2,6-Difluorobenzenesulfonyl)-4-[(2E)-3-phenylbut-2-en-1-yl] piperazine* **SU**[1,5], yellow oil, 37%, UHPLC-ESI-MS: *R*_t_ = 2.24, *m*/*z* = 393.0 [M + H]^+^. Purity (UHPLC) = 99%.

*1-(Cyclopentanesulfonyl)-4-[(2E)-3-(4-methoxyphenyl)but-2-en-1-yl] piperazine* **SU**[2,1], white solid, 9%, *R*_f_ = 0.36 (DCM/MeOH 19:1), UHPLC-ESI-MS: *R*_t_ = 2.30, *m*/*z* = 379.2 [M + H]^+^. Purity (UHPLC) = 97%. ^1^H NMR (300 MHz, CDCl_3_), δ 7.38 (dd, *J* = 2.1 Hz, *J* = 6.9 Hz, 2H), 6.87 (dd, *J* = 2.1 Hz, *J* = 6.9 Hz, 2H), 5.93 (t, *J* = 7.7 Hz, 1H), 3.82 (s, 3H), 3.80–3.77 (m, 4H), 3.50–3.40 (m, 1H), 3.11–2.94 (m, 4H), 2.10 (s, 3H), 2.03–1.91 (m, 8H) ppm; ^13^C NMR (100 MHz, CDCl_3_), δ 160.0, 146.1, 133.3, 127.3, 113.9, 111.4, 61.4, 55.7, 55.3, 51.5, 43.1, 28.0, 25.5, 16.6 ppm.

*1-(Cyclohexanesulfonyl)-4-[(2E)-3-(4-methoxyphenyl) but-2-en-1-yl]piperazine* **SU**[2,2], yellow solid, 17%, UHPLC-ESI-MS: *R*_t_ = 2.39, *m*/*z* = 393.2 [M + H]^+^. Purity (UHPLC) = 98%.

*1-[(2E)-3-(4-Methoxyphenyl)but-2-en-1-yl]-4-[4-(trifluoromethyl)benzenesulfonyl]piperazine* **SU**[2,3], white solid, 6%, UHPLC-ESI-MS: *R*_t_ = 2.62, *m*/*z* = 455.2 [M + H]^+^. Purity (UHPLC) = 85%.

*1-[(2E)-3-(4-Methoxyphenyl)but-2-en-1-yl]-4-(4-methyl benzenesulfonyl)piperazine* **SU**[2,4], yellow solid, 12%, UHPLC-ESI-MS: *R*_t_ = 2.39, *m*/*z* = 401.2 [M + H]^+^. Purity (UHPLC) = 85%.

*1-(Cyclopentanesulfonyl)-4-[(2E)-3-(naphthalen-2-yl)but-2 -en-1-yl]piperazine* **SU**[3,1], white solid, 22%, *R*_f_ = 0.44 (DCM/MeOH 19:1), UHPLC-ESI-MS: *R*_t_ = 2.60, *m*/*z* = 399.2 [M + H]^+^. Purity (UHPLC) = 97%. ^1^H NMR (300 MHz, CDCl_3_), δ 7.85–7.78 (m, 4H), 7.58 (dd, *J* = 1.8 Hz, *J* = 8.6 Hz, 1H), 7.49–7.46 (m, 2H), 6.13 (t, *J* = 7.3 Hz, 1H), 3.74 (s, 6H), 3.47–3.41 (m, 1H), 2.21 (s, 3H), 2.05–1.96 (m, 7H), 1.79–1.75 (m, 3H), 1.63–1.59 (m, 2H) ppm; ^13^C NMR (100 MHz, CDCl_3_), δ 141.3, 138.5, 135.8, 133.1, 131.4, 128.3, 128.1, 127.6, 126.5, 126.4, 125.2, 123.9, 61.2, 55.7, 51.8, 30.9, 27.9, 25.5, 16.6 ppm.

*1-(Cyclohexanesulfonyl)-4-[(2E)-3-(naphthalen-2-yl)but-2-en-1-yl]piperazine* **SU**[3,2], white solid, 20%, UHPLC-ESI-MS: *R*_t_ = 2.72, *m*/*z* = 413.2 [M + H]^+^. Purity (UHPLC) = 97%.

*1-[(2E)-3-(Naphthalen-2-yl)but-2-en-1-yl]-4-[4-(trifluoromethyl) benzenesulfonyl]piperazine* **SU**[3,3], yellow solid, 22%, UHPLC-ESI-MS: *R*_t_ = 2.85, *m*/*z* = 477.0 [M + H]^+^. Purity (UHPLC) = 93%.

*1-(4-Methylbenzenesulfonyl)-4-[(2E)-3-(naphthalen-2-yl)but-2-en-1-yl]piperazine* **SU**[3,4], white solid, 5%, UHPLC-ESI-MS: *R*_t_ = 2.79, *m*/*z* = 421.0 [M + H]^+^. Purity (UHPLC) = 96%.

#### General Procedure for Reductive Amination

Reactions were performed in parallel in 15-ml reaction tubes in a 24-position Mettler-Toledo Miniblock® equipped with a heat transfer block and inert gas manifold. Each reaction tube was loaded with a previously prepared solution of 30 mg of the corresponding amine (1.0 equiv.) in 2 mL of DCE and acetic acid (2.0 equiv). The corresponding aldehydes were added, and the mixtures were stirred at room temperature for 20 min. Afterwards, NaBH(OAc)_3_ (2.5 equiv.) was added. The reactions were stirred at room temperature overnight. Reaction conversion was confirmed through UHPLC check of some representative samples. The reaction mixtures were washed with 1 ml of water, and the organic layers were evaporated to dryness. The crudes were redissolved in 1.0 ml of ACN, filtered and purified with preparative HPLC (gradient acetonitrile/water with 0.1% formic acid, 2–98%). Fractions containing pure product were combined and evaporated to dryness in Mettler vials.

*(E)-1-Benzyl-4-(3-(naphthalen-2-yl)but-2-en-1-yl)piperazine* **RC-206**, brown solid, 28%, m.p. = 98°C, *R*_f_ = 0.37 (DCM/MeOH 95:5), ^1^H-NMR [400 MHz, (CD_3_)_2_CO], δ (ppm) 7.93–7.91 (m, 2H), 7.89–7.86 (m, 2H), 7.69–7.67 (m, 1H), 7.50–7.46 (m, 3H), 7.35–7.33 (m, 5H), 6.08 (t,1H), 3.52 (s, 2H), 3.25 (d, 2H), 2.62–2.39 (brs, 8H), 2.20 (s,3H) ppm; ^13^C-NMR [100 MHz (CD_3_)_2_CO], δ (ppm) 128.86,128.08,127.65,127.39,126.85,126.07,125.64, 124.19, 124.08, 62.49, 56.24, 53.08, 52.88, 15.55.

*1-(Cyclopentylmethyl)-4-[(2E)-3-phenylbut-2-en-1-yl]piperazine* **RA**[1,1], orange oil, 99%, UHPLC-ESI-MS: *R*_t_ = 1.82, *m*/*z* = 299.2 [M + H]^+^. Purity (UHPLC) = 99%.

*1-(Cyclohexylmethyl)-4-[(2E)-3-phenylbut-2-en-1-yl]piperazine* **RA**[1,2], orange solid, 52%, UHPLC-ESI-MS: *R*_t_ = 1.98, *m*/*z* = 313.2 [M + H]^+^. Purity (UHPLC) = 99%.

*1-[(2E)-3-Phenylbut-2-en-1-yl]-4-{[4-(trifluoromethyl)phenyl]methyl}piperazine* **RA**[1,3], brown solid, 60%, UHPLC-ESI-MS: *R*_t_ = 2.33, *m*/*z* = 375.2 [M + H]^+^. Purity (UHPLC) = 99%.

*1-[(4-Methylphenyl)methyl]-4-[(2E)-3-phenylbut-2-en-1-yl]piperazine* **RA**[1,4], orange oil, 55%, *R*_f_ = 0.41 (DCM/MeOH 19:1), UHPLC-ESI-MS: *R*_t_ = 2.07, *m*/*z* = 321.2 [M + H]^+^. Purity (UHPLC) = 99%. ^1^H NMR (300 MHz, CDCl_3_), δ 7.41–7.37 (m, 2H), 7.34–7.31 (m, 1H), 7.32–7.25 (m, 2H), 7.20 (d, *J* = 8.0 Hz, 2H), 7.13 (d, *J* = 7.9 Hz, 2H), 5.87 (dt, *J* = 1.3 Hz, *J* = 7.2 Hz, 1H), 3.58 (s, 2H), 3.39 (d, *J* = 7.2 Hz, 2H), 2.79 (s, 4H), 2.67 (s, 4H), 2.33 (s, 3H), 2.06 (s, 3H) ppm; ^13^C NMR (100 MHz, CDCl_3_), δ 142.7, 140.1, 137.2, 133.2, 129.4, 129.0, 128.2, 127.3, 125.7, 121.1, 61.9, 55.5, 51.7, 51.4, 21.1, 16.2 ppm.

*1-[(2,6-Difluorophenyl)methyl]-4-[(2E)-3-phenylbut-2-en-1-yl]piperazine* **RA**[1,5], orange oil, 42%, UHPLC-ESI-MS: *R*_t_ = 2.09, *m*/*z* = 343.2 [M + H]^+^. Purity (UHPLC) = 93%.

*1-(Cyclopentylmethyl)-4-[(2E)-3-(4-methoxyphenyl)but-2-en-1-yl]piperazine* **RA**[2,1], white solid, 37%, UHPLC-ESI-MS: *R*_t_ = 1.85, *m*/*z* = 329.2 [M + H]^+^. Purity (UHPLC) = 99%.

*1-(Cyclohexylmethyl)-4-[(2E)-3-(4-methoxyphenyl)but-2-en-1-yl]piperazine* **RA**[2,2], yellow oil, 45%, *R*_f_ = 0.22 (DCM/MeOH 19:1), UHPLC-ESI-MS: *R*_t_ = 1.99, *m*/*z* = 343.2 [M + H]^+^. Purity (UHPLC) = 99%. ^1^H NMR (300 MHz, CDCl_3_), δ 7.34 (d, *J* = 8.9 Hz, 2H), 6.85 (d, *J* = 8.9 Hz, 2H), 5.81 (dt, *J* = 1.2 Hz, *J* = 7.2 Hz, 1H), 3.80 (s, 3H), 3.36 (d, *J* = 7.2 Hz, 2H), 2.77 (s, 4H), 2.64 (s, 4H), 2.25 (d, *J* = 7.1 Hz, 2H), 2.04 (s, 3H), 1.77–1.68 (m, 5H), 1.52–1.46 (m, 1H), 1.27–1.11 (m, 3H) ppm; ^13^C NMR (100 MHz, CDCl_3_), δ 159.0, 139.3, 135.2, 126.8, 119.7, 113.6, 64.8, 55.6, 55.3, 52.2, 51.7, 34.6, 31.7, 26.5, 26.0, 16.2 ppm.

*1-[(2E)-3-(4-Methoxyphenyl)but-2-en-1-yl]-4-{[4-(trifluoromethyl)phenyl]methyl}piperazine* **RA**[2,3], orange oil, 21%, *R*_f_ = 0.30 (DCM/MeOH 19:1), UHPLC-ESI-MS: *R*_t_ = 2.36, *m*/*z* = 405.2 [M + H]^+^. Purity (UHPLC) = 99%. ^1^H NMR (300 MHz, CDCl_3_), δ 7.57 (d, *J* = 8.1 Hz, 2H), 7.44 (d, *J* = 8.0 Hz, 2H), 7.34 (d, *J* = 8.9 Hz, 2H), 6.86 (d, *J* = 8.9 Hz, 2H), 5.82 (dt, *J* = 1.3 Hz, *J* = 7.2 Hz, 1H), 3.81 (s, 3H), 3.59 (s, 2H), 3.40 (d, *J* = 7.3 Hz, 2H), 2.79 (s, 4H), 2.61 (s, 4H), 2.05 (s, 3H) ppm; ^13^C NMR (100 MHz, CDCl_3_) δ 159.1, 141.9, 139.6, 135.1, 129.7, 129.2, 128.8, 125.3 (q, *J* = 3.8Hz), 119.3, 118.6, 62.0, 55.6, 55.3, 52.1, 51.8, 16.2 ppm.

*1-[(2E)-3-(4-Methoxyphenyl)but-2-en-1-yl]-4-[(4-methylphenyl)methyl]piperazine* **RA**[2,4], yellowish solid, 15%, UHPLC-ESI-MS: *R*_t_ = 2.12, *m*/*z* = 351.2 [M + H]^+^. Purity (UHPLC) = 99%.

*1-[(2,6-Difluorophenyl)methyl]-4-[(2E)-3-(4-methoxyphenyl) but-2-en-1-yl]piperazine* **RA**[2,5], yellow oil, 43%, UHPLC-ESI-MS: *R*_t_ = 2.13, *m*/*z* = 373.2 [M + H]^+^. Purity (UHPLC) = 95%.

*1-(Cyclopentylmethyl)-4-[(2E)-3-(naphthalen-2-yl)but-2-en-1-yl]piperazine* **RA**[3,1], colorless oil, 28%, *R*_f_ = 0.33 (DCM/MeOH 19:1), UHPLC-ESI-MS: *R*_t_ = 2.17, *m*/*z* = 349.2 [M + H]^+^. Purity (UHPLC) = 99%. ^1^H NMR (300 MHz, CDCl_3_), δ 7.85–7.78 (m, 4H), 7.57 (dd, *J* = 1.9 Hz, *J* = 8.6 Hz, 1H), 7.49–7.45 (m, 2H), 5.97 (dt, *J* = 1.3 Hz, *J* = 7.3 Hz, 1H), 3.53–3.43 (m, 2H), 3.00 (s, 8H), 2.70 (d, *J* = 7.3 Hz, 2H), 2.21 (s, 3H), 1.89–1.80 (m, 2H), 1.68–1.55 (m, 5H), 1.29–1.20 (m, 2H) ppm; ^13^C NMR (100 MHz, CDCl_3_), δ 141.4, 139.5, 133.2, 132.8, 128.2, 127.9, 127.5, 126.3, 126.0, 124.6, 124.0, 120.4, 63.0, 55.4, 51.7, 50.3, 35.8, 31.5, 25.0, 16.5 ppm.

*1-(Cyclohexylmethyl)-4-[(2E)-3-(naphthalen-2-yl)but-2-en-1-yl]piperazine* **RA**[3,2], white solid, 53%, UHPLC-ESI-MS: *R*_t_ = 2.30, *m*/*z* = 363.2 [M + H]^+^. Purity (UHPLC) = 95%.

*1-[(2E)-3-(Naphthalen-2-yl)but-2-en-1-yl]-4-{[4-(trifluoromethyl)phenyl]methyl}piperazine* **RA**[3,3], yellow oil, 42%, UHPLC-ESI-MS: *R*_t_ = 2.56 *m*/*z* = 425.2 [M + H]^+^. Purity (UHPLC) = 98%.

*1-[(4-Methylphenyl)methyl]-4-[(2E)-3-(naphthalen-2-yl) but-2-en-1-yl]piperazine* **RA**[3,4], yellow oil, 16%, UHPLC-ESI-MS: *R*_t_ = 2.38 *m*/*z* = 371.2 [M + H]^+^. Purity (UHPLC) = 88%.

*1-[(2,6-Difluorophenyl)methyl]-4-[(2E)-3-(naphthalen-2-yl)but-2-en-1-yl]piperazine* **RA**[3,5], yellow oil, 26%, UHPLC-ESI-MS: *R*_t_ = 2.39 *m*/*z* = 393.2 [M + H]^+^. Purity (UHPLC) = 88%.

*1-(Cyclopentylmethyl)-4-[(2E)-3-(6-methoxynaphthalen-2-yl)but-2-en-1-yl]piperazine* **RA**[4,1], colorless oil, 5%, UHPLC-ESI-MS: *R*_t_ = 2.18 *m*/*z* = 379.2 [M + H]^+^. Purity (UHPLC) = 99%.

*1-[(2E)-3-(6-Methoxynaphthalen-2-yl)but-2-en-1-yl]-4-{[4-(trifluoromethyl)phenyl]methyl}piperazine* **RA**[4,3], colorless oil, 5%, UHPLC-ESI-MS: *R*_t_ = 2.57 *m*/*z* = 455.2 [M + H]^+^. Purity (UHPLC) = 88%.

*1-[(2,6-Difluorophenyl)methyl]-4-[(2E)-3-(6-methoxynaphthalen-2-yl)but-2-en-1-yl]piperazine* **RA**[4,5], white solid, 21%, *R*_f_ = 0.50 (DCM/MeOH 19:1), UHPLC-ESI-MS: *R*_t_ = 2.38, *m*/*z* = 423.0 [M + H]^+^. Purity (UHPLC) = 95%. Purity (UHPLC) = 97%. ^1^H NMR (300 MHz, CDCl_3_), δ 7.75–7.68 (m, 3H), 7.53 (dd, *J* = 1.9 Hz, *J* = 8.6 Hz, 1H), 7.32–7.27 (m, 1H), 7.16–7.11 (m, 2H), 6.93–6.87 (m, 2H), 5.99 (dd, *J* = 6.6 Hz, *J* = 7.9 Hz, 1H), 3.92 (s, 3H), 3.76 (s, 2H), 3.61 (d, *J* = 7.5 Hz, 2H), 2.99 (s, 4H), 2.80 (s, 4H), 2.18 (s, 3H) ppm; ^13^C NMR (100 MHz, CDCl_3_), δ 160.3 (d, *J* = 8.3 Hz), 157.9, 142.9, 136.8, 134.2, 129.9, 129.8, 128.7, 128.6, 126.9, 124.7, 124.4, 119.2, 111.5, 111.1, 105.5, 55.3, 55.1, 51.7, 50.0, 48.1, 16.4 ppm.

#### General Procedure for the Amide Formation

Reactions were performed in parallel in 15-ml reaction tubes in a 24-position Mettler-Toledo Miniblock® equipped with a heat transfer block and inert gas manifold. Each reaction tube was loaded with a previously prepared solution of 30 mg of the corresponding amine (1.0 equiv.) in 2 ml of DMF, DIPEA (5.0 equiv.), HOBt (2.0 equiv.), EDC^*^HCl (2.5 equiv.). The corresponding acids were added (2.0 equiv.). The reaction mixtures were stirred at room temperature overnight. Reaction conversion was confirmed through UHPLC check of some representative samples. The mixtures were evaporated until dryness. The crudes were redissolved in 1.0 ml of ACN, filtered and purified with preparative HPLC (gradient acetonitrile/water with 0.1% formic acid, 2–98%). Fractions containing pure product were combined and evaporated to dryness in Mettler vials.

*1-Cyclopentanecarbonyl-4-[(2E)-3-phenylbut-2-en-1-yl]piperazine* **AM**[1,1], brown oil, 72%, UHPLC-ESI-MS: *R*_t_ = 2.13 *m*/*z* = 313.2 [M + H]^+^. Purity (UHPLC) = 96%.

*1-Cyclohexanecarbonyl-4-[(2E)-3-phenylbut-2-en-1-yl]piperazine* **AM**[1,2], orange oil, 70%, UHPLC-ESI-MS: *R*_t_ = 2.20 *m*/*z* = 327.2 [M + H]^+^. Purity (UHPLC) = 99%.

*1-[(2E)-3-Phenylbut-2-en-1-yl]-4-[4-(trifluoromethyl)benzoyl]piperazine* **AM**[1,3], orange oil, 54%, UHPLC-ESI-MS: *R*_t_ = 2.39 *m*/*z* = 389.2 [M + H]^+^. Purity (UHPLC) = 99%.

*1-(4-Methylbenzoyl)-4-[(2E)-3-phenylbut-2-en-1-yl]piperazine* **AM**[1,4], orange oil, 78%, UHPLC-ESI-MS: *R*_t_ = 2.21 *m*/*z* = 335.2 [M + H] ^+^. Purity (UHPLC) = 99%.

*1-(2,6-Difluorobenzoyl)-4-[(2E)-3-phenylbut-2-en-1-yl]piperazine* **AM**[1,5], orange oil, 31%, *R*_f_ = 0.50 (DCM/MeOH 19:1), UHPLC-ESI-MS: *R*_t_ = 2.13, *m*/*z* = 357.2 [M + H]^+^. Purity (UHPLC) = 95%. ^1^H NMR (300 MHz, CDCl_3_), δ 7.41–7.28 (m, 6H), 6.95 (dd, *J* = 7.3 Hz, *J* = 8.4 Hz, 2H), 5.87 (dt, *J* = 1.3 Hz, *J* = 7.0 Hz, 1H), 3.94–3.91 (m, 2H), 3.44–3.41 (m, 2H), 3.32 (d, *J* = 7.1 Hz, 2H), 2.73–2.70 (m, 2H), 2.63–2.59 (m, 2H), 2.08 (s, 3H) ppm; ^13^C NMR (100 MHz, CDCl_3_), δ 160.0, 157.2 (d, *J* = 7.9 Hz), 142.9, 139.5, 131.1, 128.3, 127.3, 125.7, 122.3, 113.5, 111.8 (d, *J* = 24.9 Hz), 56.1, 52.9, 52.3, 46.3, 41.4, 16.3 ppm.

*1-Cyclopentanecarbonyl-4-[(2E)-3-(4-methoxyphenyl)but-2-en-1-yl]piperazine* **AM**[2,1], yellow oil, 37%, UHPLC-ESI-MS: *R*_t_ = 2.11*m*/*z* = 343.2 [M + H]^+^. Purity (UHPLC) = 99%.

*1-Cyclohexanecarbonyl-4-[(2E)-3-(4-methoxyphenyl)but-2-en-1-yl]piperazine* **AM**[2,2], yellow oil, 32%, UHPLC-ESI-MS: *R*_t_ = 2.21 *m*/*z* = 357.2 [M + H]^+^. Purity (UHPLC) = 99%.

*1-[(2E)-3-(4-Methoxyphenyl)but-2-en-1-yl]-4-[4-(trifluoromethyl)benzoyl]piperazine* **AM**[2,3], yellow oil, 36%, UHPLC-ESI-MS: *R*_t_ = 2.35 *m*/*z* = 419.0 [M + H]^+^. Purity (UHPLC) = 99%.

*1-[(2E)-3-(4-Methoxyphenyl)but-2-en-1-yl]-4-(4-methylbenzoyl)piperazine* **AM**[2,4], yellow oil, 34%, *R*_f_ = 0.35 (DCM/MeOH 19:1), UHPLC-ESI-MS: *R*_t_ = 2.23, *m*/*z* = 365.2 [M + H]^+^. Purity (UHPLC) = 98%. ^1^H NMR (300 MHz, CDCl_3_), δ 7.35–7.29 (m, 4H), 7.21 (d, *J* = 7.8 Hz, 2H), 6.86 (dd, *J* = 2.1 Hz, *J* = 9.0 Hz, 2H), 5.79 (dt, *J* = 1.2 Hz, *J* = 7.2 Hz, 1H), 3.96–3.87 (m, 1H), 3.81 (s, 3H), 3.71–3.59 (m, 2H), 3.40 (d, *J* = 7.3 Hz, 2H), 2.80–2.61 (m, 5H), 2.37 (s, 3H), 2.05 (d, *J* = 1.0 Hz, 3H), ppm; ^13^C NMR (100 MHz, CDCl_3_), δ 170.6, 159.1, 140.3, 140.1, 135.0, 132.1, 129.1, 127.2, 126.8, 118.7, 113.6, 55.7, 55.3, 52.2, 41.0, 21.4, 16.3 ppm.

*1-(2,6-Difluorobenzoyl)-4-[(2E)-3-(4-methoxyphenyl)but-2-en-1-yl]piperazine* **AM**[2,5], yellow oil, 15%, UHPLC-ESI-MS: *R*_t_ = 2.16 *m*/*z* = 387.2 [M + H]^+^. Purity (UHPLC) = 95%.

*1-Cyclopentanecarbonyl-4-[(2E)-3-(naphthalen-2-yl)but-2-en-1-yl]piperazine* **AM**[3,1], yellow oil, 35%, *R*_f_ = 0.37 (DCM/MeOH 19:1), UHPLC-ESI-MS: *R*_t_ = 2.42, *m*/*z* = 363.2 [M + H]^+^. Purity (UHPLC) = 98%. ^1^H NMR (300 MHz, CDCl_3_), δ 9.75 (s br, 1H), 7.85–7.78 (m, 4H), 7.57 (dd, *J* = 1.8 Hz, *J* = 8.6 Hz, 1H), 7.48–7.44 (m, 2H), 6.03 (dt, *J* = 1.2 Hz, *J* = 7.1 Hz, 1H), 3.79–3.76 (m, 2H), 3.69–3.65 (m, 2H), 3.43 (d, *J* = 7.1 Hz, 2H), 2.90–2.85 (m, 1H), 2.76–2.72 (m, 4H), 2.18 (s, 3H), 1.83–1.67 (m, 5H), 1.60–1.55 (m, 3H) ppm; ^13^C NMR (100 MHz, CDCl_3_), δ 174.6, 140.1, 139.8, 133.3, 132.7, 128.1, 127.8, 127.5, 126.2, 125.9, 124.5, 124.1, 121.6, 55.9, 52.6, 44.5, 40.9, 30.1, 26.0, 16.3 ppm.

*1-Cyclohexanecarbonyl-4-[(2E)-3-(naphthalen-2-yl)but-2-en-1-yl]piperazine* **AM**[3,2], yellow oil, 99%, UHPLC-ESI-MS: *R*_t_ = 2.52 *m*/*z* = 377.2 [M + H]^+^. Purity (UHPLC) = 97%.

*1-(2,6-Difluorobenzoyl)-4-[(2E)-3-(naphthalen-2-yl)but-2-en-1-yl]piperazine* **AM**[3,5], colorless oil, 6%, *R*_f_ = 0.42 (DCM/MeOH 19:1), UHPLC-ESI-MS: *R*_t_ = 2.53, *m*/*z* = 407.0 [M + H]^+^. Purity (UHPLC) = 85%. ^1^H NMR (300 MHz, CDCl_3_), δ 7.81 (d, *J* = 4.4 Hz, 3H), 7.70 (d, *J* = 8.3 Hz, 1H), 7.57 (dd, *J* = 1.8 Hz, *J* = 8.6 Hz, 1H), 7.48–7.44 (m, 2H), 7.38–7.35 (m, 1H), 6.99–6.94 (m, 2H), 6.07 (dt, *J* = 1.0 Hz, *J* = 7.2 Hz, 1H), 4.04 (s, 2H), 3.56–3.49 (m, 4H), 2.93–2.89 (m, 2H), 2.83–2.80 (m, 2H), 2.21 (s, 3H) ppm; ^13^C NMR (100 MHz, CDCl_3_), δ 168.5, 160.0, 139.7, 133.3, 132.8, 131.3, 130.3, 128.1, 127.9, 127.5, 126.2, 126.0, 125.4 (d, *J* = 3.7 Hz), 124.6, 124.1, 112.1 (d, *J* = 3.0 Hz), 111.8 (d, *J* = 3.4 Hz), 55.9, 52.5, 52.0, 45.8, 40.8, 16.4 ppm.

### *In silico* Studies

Prediction of some basic absorption, distribution, metabolism, and excretion (ADME) parameters, most importantly solubility, was performed with the SwissADME web tool. This open-access and user-friendly tool is accessible at http://www.swissadme.ch. SMILES strings corresponding to all the designed compounds, both as free bases and protonated species, were submitted to the software.

### Biological Assays

#### Cell Culture

Human multiple myeloma RPMI 8226 cells were cultured in RPMI 1640 medium supplemented with 10% fetal bovine serum, 1% l-glutamine, and 1% penicillin and streptomycin (Euroclone, Italy). Human glioblastoma U87-MG cells were cultured in Dulbecco's modified Eagle's medium (DMEM) low glucose supplemented with 10% fetal bovine serum, 1% l-glutamine, and 1% penicillin and streptomycin (Euroclone, Italy). Cells were incubated in a humidified incubator at 37°C and 5% CO_2_. A stock solution of all compounds in dimethyl sulfoxide (DMSO) (50 mM) has been prepared and then directly diluted in culture medium.

#### MTT Assay

Cells were seeded in 96-well plates at a density of 1 × 10^4^ cells/well and were treated after 24 h with RA, AM, or SU molecules. After 24 h, a 3-(4,5-dimethylthiazol-2-yl)-2,5-diphenyltetrazolium bromide (MTT, Sigma Aldrich, United States) solution was added to each well to reach a final concentration of 0.5 mg/ml. After 2 h of incubation (4 h for RPMI 8226 cells), formazan salt was solubilized in ethanol, and absorbance was measured at 570 nm in a microplate reader (BMG-Labtech, Germany).

#### Trypan Blue Cell Viability Assay

Cells were seeded in six-well plates at a density of 2.5 × 10^5^ cells/well and were treated after 24 h with different concentrations of RA molecules. After 24 h of treatment, cells were collected and stained with Trypan blue vital dye (Sigma Aldrich, United States). Viable and dead cells were then counted in a hemocytometer under a light microscope.

#### Proteasome Activity Assay

Cells not used in Trypan blue assay were lysed to assess proteasome activity. Briefly, cells were resuspended in a lysis buffer [50 mM Hepes pH 7.5, 150 mM NaCl, 10% glycerol, 1% Triton X-100, 1.5 mM MgCl_2_, 5 mM ethylene glycol tetraacetic acid (EGTA)] and were mechanically lysed with a vortex. Obtained protein lysates were centrifuged at 13,500 rpm for 15 min and were quantified using the Bradford method. Forty micrograms of proteins was then loaded in black 96-well plate to perform the proteasome activity assay. In each well, 7.6 mg/ml proteasome substrate (*N*-succinyl-Leu-Leu-Val-Tyr-7-Amido-4-methylcoumarin, Sigma Aldrich, United States) and proteasome buffer [250 mM Hepes pH 7.5, 5 mM ethylenediaminetetraacetic acid (EDTA) pH 8.0, 0.5% NP-40, 0.01% sodium dodecyl sulfate (SDS)] were added to proteins. Fluorescence was measured after 2 h of incubation in a microplate reader (excitation, 380 nm; emission, 460 nm; BMG-Labtech, Germany).

#### Statistical Analysis

Data showed the mean ± standard deviation (*SD*) from at least three independent experiments. Statistical analysis was performed using GraphPad Prism 3 software. The differences between control and treated cells were evaluated using one-way ANOVA analysis of variance followed by Dunnet's multiple comparison test. Statistical significance was set at *p* < 0.05 and *p* < 0.01.

## Results

### Compound Library Design and *in silico* Evaluation

As a first step of this work, we synthesized compound **RC-206** (Figure 1), characterized by the presence of a piperazine as a versatile moiety suitable for different types of derivatization. Once we verified that this structural change does not significantly affect the cytotoxic activity, three different series of compounds, named **RA**, **SU**, and **AM**, have been designed, thus obtaining the library of 60 members (Table 1). Compounds are identified by the series name (which refers to the final reaction exploited) and matrix, indicating the variable portions of the final molecules. For each compound, water solubility and lipophilicity were predicted with the SwissADME web tool (Daina et al., 2017), which allows to compare the outputs of different computational methods. In detail, five freely available predictive models are employed in SwissADME to estimate log *P*_O/W_ (i.e., XLOGP3 (Cheng et al., 2007), WLOGP (Wildman and Crippen, 1999), MLOGP (Souza et al., 2011), SILICOS-IT (“Silicos-It | Filter-It™” n.d.^2^), and iLOGP Daina et al., 2014), and the consensus log *P*_O/W_ is calculated as the arithmetic mean of the values derived from these methods. On the other hand, three different models used to predict water solubility: the ESOL model (Delaney, 2004), an adaptation of the one developed by Ali et al. (2012) and the one by SILICOS-IT (“Silicos-It | Filter-It™” n.d.). The solubility predicted for the majority of the compound library, including **RC-206**, is enhanced with only a few compounds having log *S* and log *P-*values close to that of **RC-106**. Moreover, most of the designed compounds are predicted to cross both the gastrointestinal (GI) tract and the blood–brain barrier (BBB). Lastly, no pan-assay interference compounds (PAINS) (Baell and Walters, 2014; Dahlin et al., 2015) have been identified among our molecules. Data retrieved from the software are presented in Tables S1, S2. Overall, results thus obtained suggested a promising drug-like profile for the designed compound library, with improved solubility respect to *hit* compound and the desired BBB permeability and absorption requirements. Hence, all these molecules have been selected for the synthesis and experimental investigation.

**Table 1:** Compound library of **RC-106** analogs.

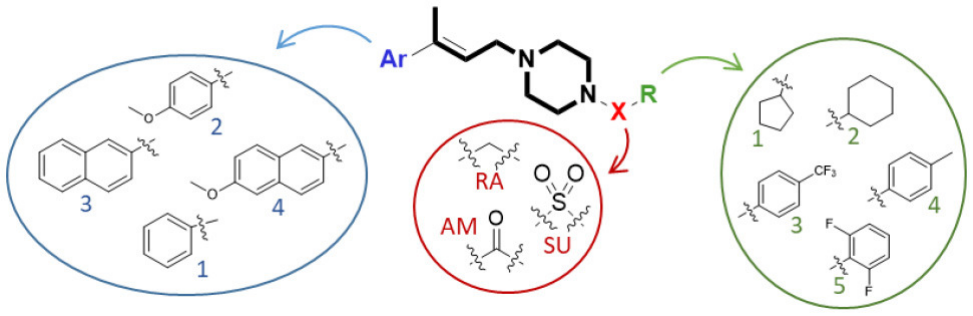							
**Name**	**X**	**Ar**	**R**	**Name**	**X**	**Ar**	**R**
**SU**[1,1]	SO_2_	phenyl	cyclopentyl	**RA**[3,1]	CH_2_	naphthalen-2-yl	cyclopentyl
**SU**[1,2]	SO_2_	phenyl	cyclohexyl	**RA**[3,2]	CH_2_	naphthalen-2-yl	cyclohexyl
**SU**[1,3]	SO_2_	phenyl	4-(trifluoromethyl)phenyl	**RA**[3,3]	CH_2_	naphthalen-2-yl	4-(trifluoromethyl)phenyl
**SU**[1,4]	SO_2_	phenyl	*p*-tolyl	**RA**[3,4]	CH_2_	naphthalen-2-yl	*p*-tolyl
**SU**[1,5]	SO_2_	phenyl	2,6-difluorophenyl	**RA**[3,5]	CH_2_	naphthalen-2-yl	2,6-difluorophenyl
**SU**[2,1]	SO_2_	4-methoxyphemnyl	cyclopentyl	**RA**[4,1]	CH_2_	6-methoxynaphthalen-2-yl	cyclopentyl
**SU**[2,2]	SO_2_	4-methoxyphemnyl	cyclohexyl	**RA**[4,2]	CH_2_	6-methoxynaphthalen-2-yl	cyclohexyl
**SU**[2,3]	SO_2_	4-methoxyphemnyl	4-(trifluoromethyl)phenyl	**RA**[4,3]	CH_2_	6-methoxynaphthalen-2-yl	4-(trifluoromethyl)phenyl
**SU**[2,4]	SO_2_	4-methoxyphemnyl	*p*-tolyl	**RA**[4,4]	CH_2_	6-methoxynaphthalen-2-yl	*p*-tolyl
**SU**[2,5]	SO_2_	4-methoxyphemnyl	2,6-difluorophenyl	**RA**[4,5]	CH_2_	6-methoxynaphthalen-2-yl	2,6-difluorophenyl
**SU**[3,1]	SO_2_	naphthalen-2-yl	cyclopentyl	**AM**[1,1]	CO	phenyl	cyclopentyl
**SU**[3,2]	SO_2_	naphthalen-2-yl	cyclohexyl	**AM**[1,2]	CO	phenyl	cyclohexyl
**SU**[3,3]	SO_2_	naphthalen-2-yl	4-(trifluoromethyl)phenyl	**AM**[1,3]	CO	phenyl	4-(trifluoromethyl)phenyl
**SU**[3,4]	SO_2_	naphthalen-2-yl	*p*-tolyl	**AM**[1,4]	CO	phenyl	*p*-tolyl
**SU**[3,5]	SO_2_	naphthalen-2-yl	2,6-difluorophenyl	**AM**[1,5]	CO	phenyl	2,6-difluorophenyl
**SU**[4,1]	SO_2_	6-methoxynaphthalen-2-yl	cyclopentyl	**AM**[2,1]	CO	4-methoxyphemnyl	cyclopentyl
**SU**[4,2]	SO_2_	6-methoxynaphthalen-2-yl	cyclohexyl	**AM**[2,2]	CO	4-methoxyphemnyl	cyclohexyl
**SU**[4,3]	SO_2_	6-methoxynaphthalen-2-yl	4-(trifluoromethyl)phenyl	**AM**[2,3]	CO	4-methoxyphemnyl	4-(trifluoromethyl)phenyl
**SU**[4,4]	SO_2_	6-methoxynaphthalen-2-yl	*p*-tolyl	**AM**[2,4]	CO	4-methoxyphemnyl	*p*-tolyl
**SU**[4,5]	SO_2_	6-methoxynaphthalen-2-yl	2,6-difluorophenyl	**AM**[2,5]	CO	4-methoxyphemnyl	2,6-difluorophenyl
**RA**[1,1]	CH_2_	phenyl	cyclopentyl	**AM**[3,1]	CO	naphthalen-2-yl	cyclopentyl
**RA**[1,2]	CH_2_	phenyl	cyclohexyl	**AM**[3,2]	CO	naphthalen-2-yl	cyclohexyl
**RA**[1,3]	CH_2_	phenyl	4-(trifluoromethyl)phenyl	**AM**[2,3]	CO	naphthalen-2-yl	4-(trifluoromethyl)phenyl
**RA**[1,4]	CH_2_	phenyl	*p*-tolyl	**AM**[2,4]	CO	naphthalen-2-yl	*p*-tolyl
**RA**[1,5]	CH_2_	phenyl	2,6-difluorophenyl	**AM**[3,5]	CO	naphthalen-2-yl	2,6-difluorophenyl
**RA**[2,1]	CH_2_	4-methoxyphemnyl	cyclopentyl	**AM**[4,1]	CO	6-methoxynaphthalen-2-yl	cyclopentyl
**RA**[2,2]	CH_2_	4-methoxyphemnyl	cyclohexyl	**AM**[4,2]	CO	6-methoxynaphthalen-2-yl	cyclohexyl
**RA**[2,3]	CH_2_	4-methoxyphemnyl	4-(trifluoromethyl)phenyl	**AM**[4,3]	CO	6-methoxynaphthalen-2-yl	4-(trifluoromethyl)phenyl
**RA**[2,4]	CH_2_	4-methoxyphemnyl	*p*-tolyl	**AM**[4,4]	CO	6-methoxynaphthalen-2-yl	*p*-tolyl
**RA**[2,5]	CH_2_	4-methoxyphemnyl	2,6-difluorophenyl	**AM**[4,5]	CO	6-methoxynaphthalen-2-yl	2,6-difluorophenyl

*The acronym **SU**, **RA**, and **AM** corresponds to the final reaction to obtain the compounds, respectively, sulfonylation, reductive amination, and amidation. The first index of the matrix identifies the aryl group, while the second index is correlated to the final R moiety*.

### Chemistry

**Scheme 1:**
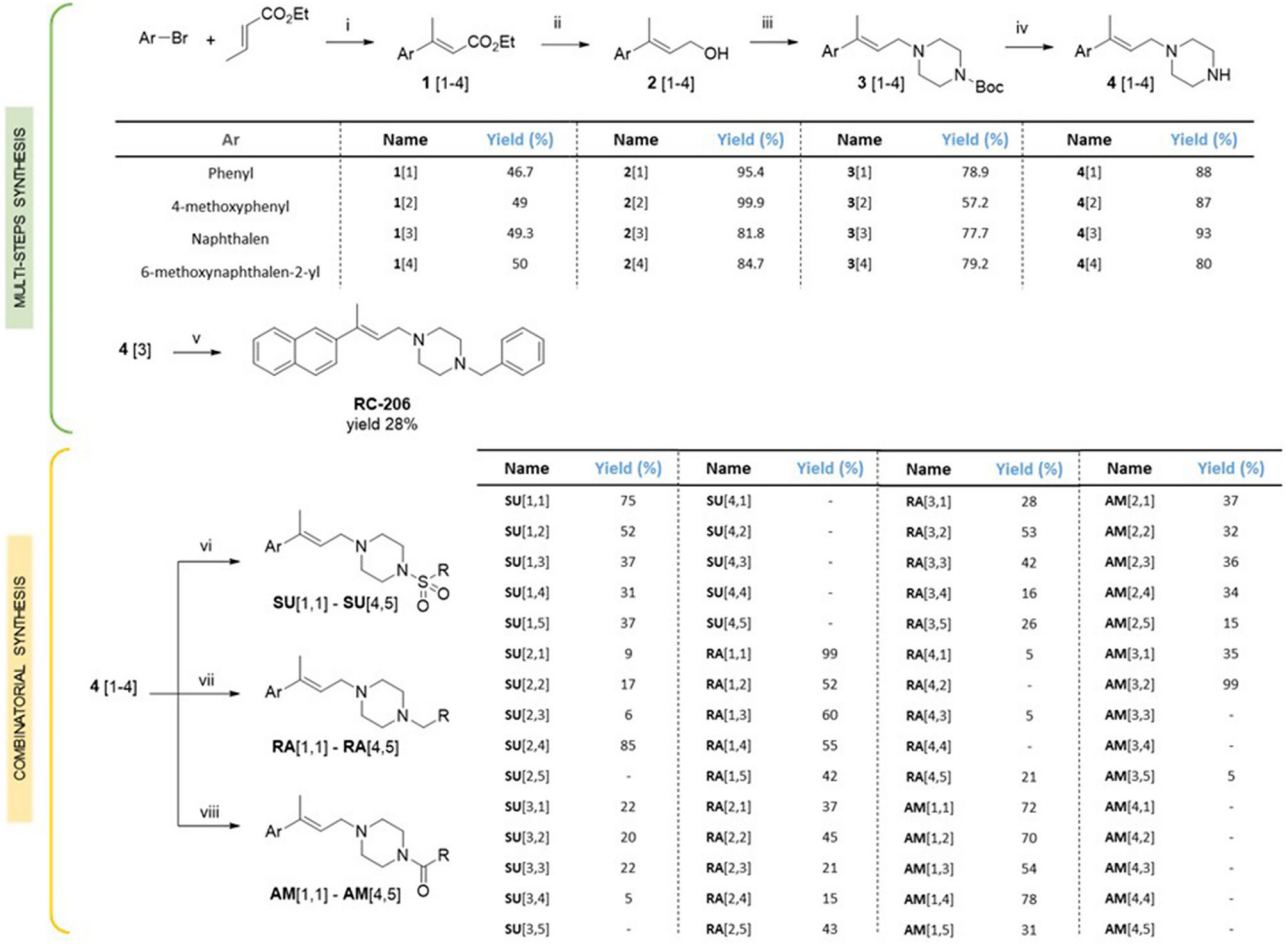
Synthesis of the compound library. Reagents and reaction conditions: (i) Pd EnCat (0.01 equiv.), TEAC (2 equiv.), AcONa (2 equiv.), dry DMF, 105 °C; (ii) LiAlH_4_ (1M in THF, 1 equiv.), dry Et_2_O, 0°C; (iii) PPh_3_ (1.5 equiv.), NBS (1.4 equiv.), 1-Boc-piperazine (1.2 equiv.), Et_3_N (2 equiv.), THF, −18°C; (iv) HCl 4 N, 1,4-dioxane, 0°C to RT; (v) benzaldehyde (1 equiv.), AcOH (2 equiv.), DCE (2 equiv.), RT, 20 min, then NaBH(OAc)_3_ (2.5 equiv.), RT, o.n.; (vi) RSO_2_Cl (1.5 equiv.), TEA (5 equiv.), DCM, RT, o.n.; (vii) RCHO (1 equiv.), AcOH (2 equiv.), DCE (2 equiv.), RT, 20 min, then NaBH(OAc)_3_ (2.5 equiv.), RT, o.n.; (viii) RCO_2_H (2 equiv.), DIPEA (5 equiv.), HOBt (2 equiv.), EDC·HCl (2.5 equiv.), DMF, RT, o.n.

For the synthesis of compound **RC-206** (Figure 1) and the library of derivatives (Table 1) the procedure already set up for **RC-106** (Rui et al., 2016b) has been optimized, and a final diversification step, suitable for combinatorial synthesis, was added as reported in Scheme 1. In detail, Heck reaction was exploited to prepare the α,β-unsaturated esters **1**[1–4]. Particularly, the reaction was optimized using Pd EnCat® 40 (palladium acetate microencapsulated in polyurea matrix) instead of ordinary palladium acetate, to both simplify the workup and reduce its exposure to air while limiting palladium contamination in the resulting products. The isolated (*E*) α,β-unsaturated esters were then reduced with LiAlH_4_ to prepare allylic alcohols **2**[1–4]. The latter were reacted with *N*-Boc-piperazine according to the procedure described by Frøyen (Frøyen and Juvvik, 1995). This reaction consists in a nucleophilic substitution *via* alkoxyphosphonium salt, generated by the addition of triphenylphosphine (PPh_3_) and *N*-bromosuccinimide (NBS) to the alcohol. The protocol reported in the literature was slightly modified in order to find the optimal conditions (i.e., reagent equivalents, temperature, and timing of reagent additions) to access compounds **3**[1–4]. Upon Boc- deprotection with TFA, key intermediates **4**[1–4] were isolated in quantitative yield. At this point, to prepare **RC-206**, a bench-scale reductive amination was performed on intermediate **4**[3]. Conversely, for the preparation of the library, a combinatorial approach was exploited, and the piperazinic nitrogen was derivatized using three different reactions: sulfonylation (Scheme 1, **SU**[1,1]–**SU**[4,5]), reductive amination (Scheme 1, **RA**[1,1]–**RA**[4,5]), and amide coupling (Scheme 1, **AM**[1,1]–**AM**[4,5]). The preparation of the derivatives was performed in a parallel fashion using a 24-position Mettler-Toledo block equipped with 15-ml reaction tubes (Scheme 1).

The compounds were characterized by NMR, UPLC-MS, and IR analyses. Overall, 44 compounds were obtained from the combinatorial synthesis in suitable amount and purity for subsequent biological investigations.

### Biological Investigation

The cytotoxic activities of all synthesized compounds were evaluated *in vitro* via MTT assay against the two human cancer cell lines U87MG and RPMI 8226, representative of human glioblastoma multiforme (GBM) and multiple myeloma, respectively.

**Figure 2:**
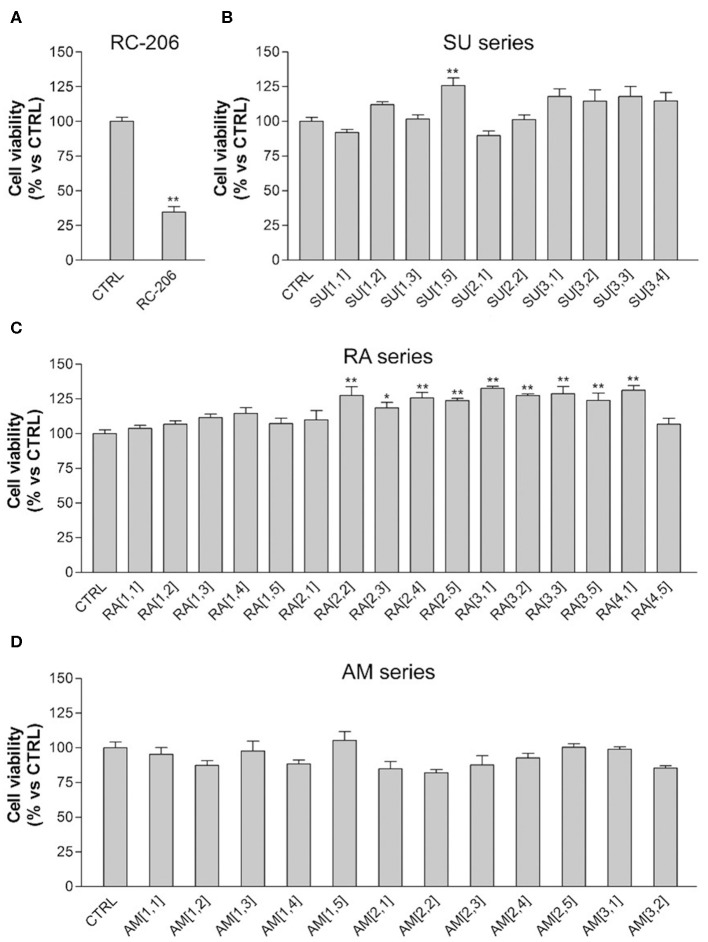
3-(4,5-Dimethylthiazol-2-yl)-2,5-diphenyltetrazolium bromide (MTT) assay, U87-MG cells treated with test compounds for 24 h at 40 μM concentration. Results are expressed as cell viability (%). **(A) RC-206**. **(B) SU** molecules. **(C) RA** molecules. **(D) AM** molecules. All graphs are represented as the mean percentage ± *SD* of three independent experiments and are compared to untreated controls (CTRL) arbitrarily set to 100%. *p < 0.05 vs. CTRL; **p < 0.01 vs. CTRL.

**Figure 3:**
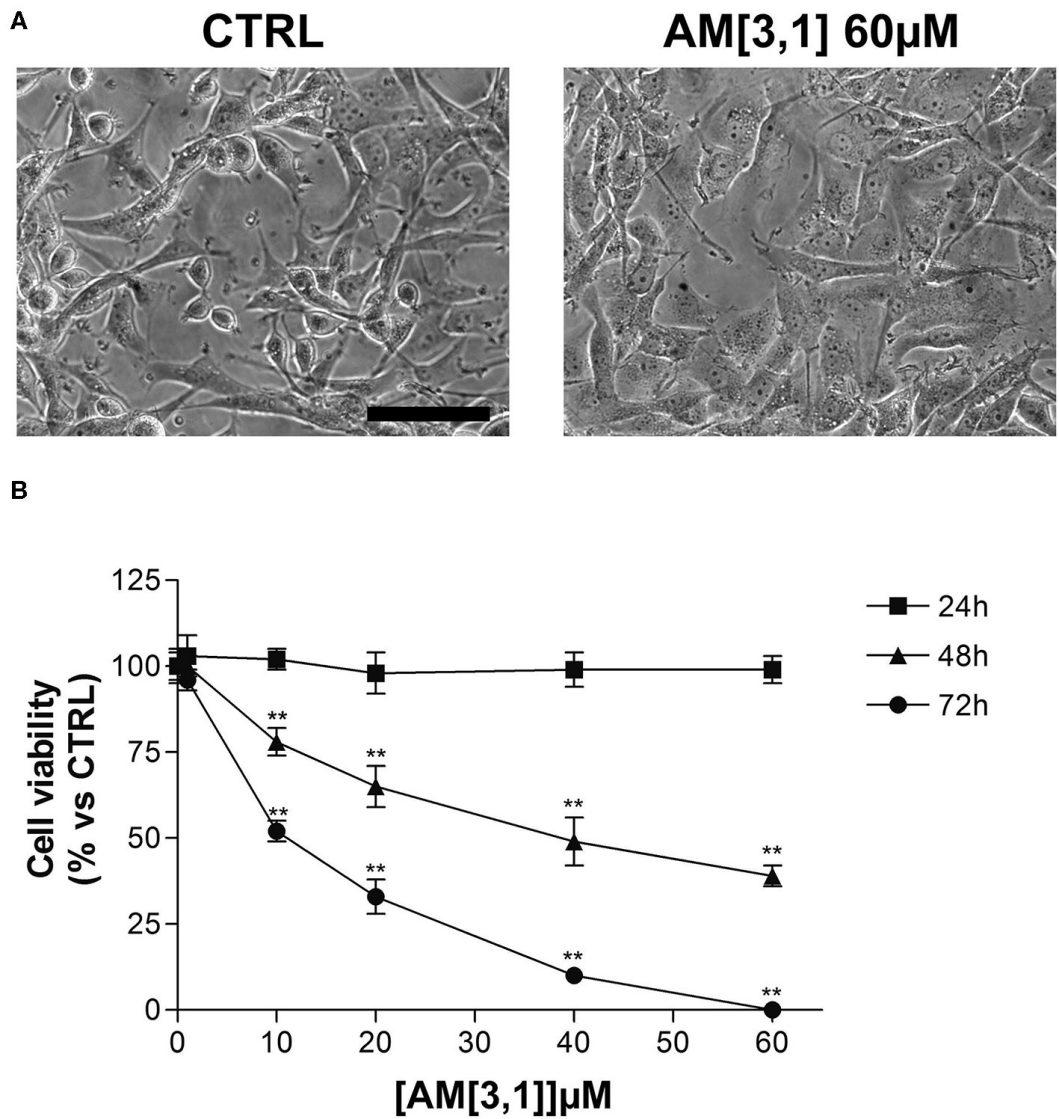
U87-MG cell morphological alterations and 3-(4,5-dimethylthiazol-2-yl)-2,5-diphenyltetrazolium bromide (MTT) assay after **AM**[3,1] treatment. **(A)** Morphology of U87-MG cells, untreated (CTRL), or treated with **AM**[3,1] 60 μM. Scale bar, 80 μm. **(B)** MTT assay of U87-MG cells treated with increasing concentrations of **AM**[3,1] (1–60 μM) for 24, 48, and 72 h. Graph represents the mean percentage ± *SD* of viable cells compared to untreated control cells arbitrarily set to 100%. **p < 0.01 vs. CTRL.

In particular, the cell viability of U87-MG cells was assessed by MTT assay after 24 h of continuous treatment with all synthesized compounds, at the concentration of 60 μM, corresponding to the **RC-106** IC_50_ value observed on the same cell lines after a similar time exposure. In this preliminary screening, **RC-206** showed a moderate activity against U87-MG cells after 24 h of treatment, reducing the cell viability to 34% (Figure 2A). Conversely, all compounds belonging to **RA**, **SU**, and **AM** series (Figures 2B–D) did not show any cell viability reduction. Nevertheless, compound **AM**[3,1] induced an alteration of cell morphology. Actually, treated U87-MG cells showed an epithelial-like morphology, characterized by a flat cell body, and by the presence of tight junctions between cells, whereas U87-MG control cells had an elongated cell body, and no or few tight junctions between near cells (Figure 3A). Therefore, the cytotoxicity of **AM**[3,1] compound at long-term exposure times was evaluated, determining the IC_50_ values at 48 and 72 h (Figure 3B). A time-dependent effect in the micromolar range on cell viability was observed (IC_50_, 39.05 and 11.21 μM, at 48 and 72 h, respectively).

We also observed in this preliminary screening that 3 out of 20 compound of **RA** series (**RA**[2,2], **RA**[3,1], **RA**[4,1]) and only 1 out of 20 molecules belonging to SU series (**SU**[1,5]) induced a slight, but significant, increase in cell viability, which is ~25% higher than in untreated controls (Figures 2A–C).

**Figure 4:**
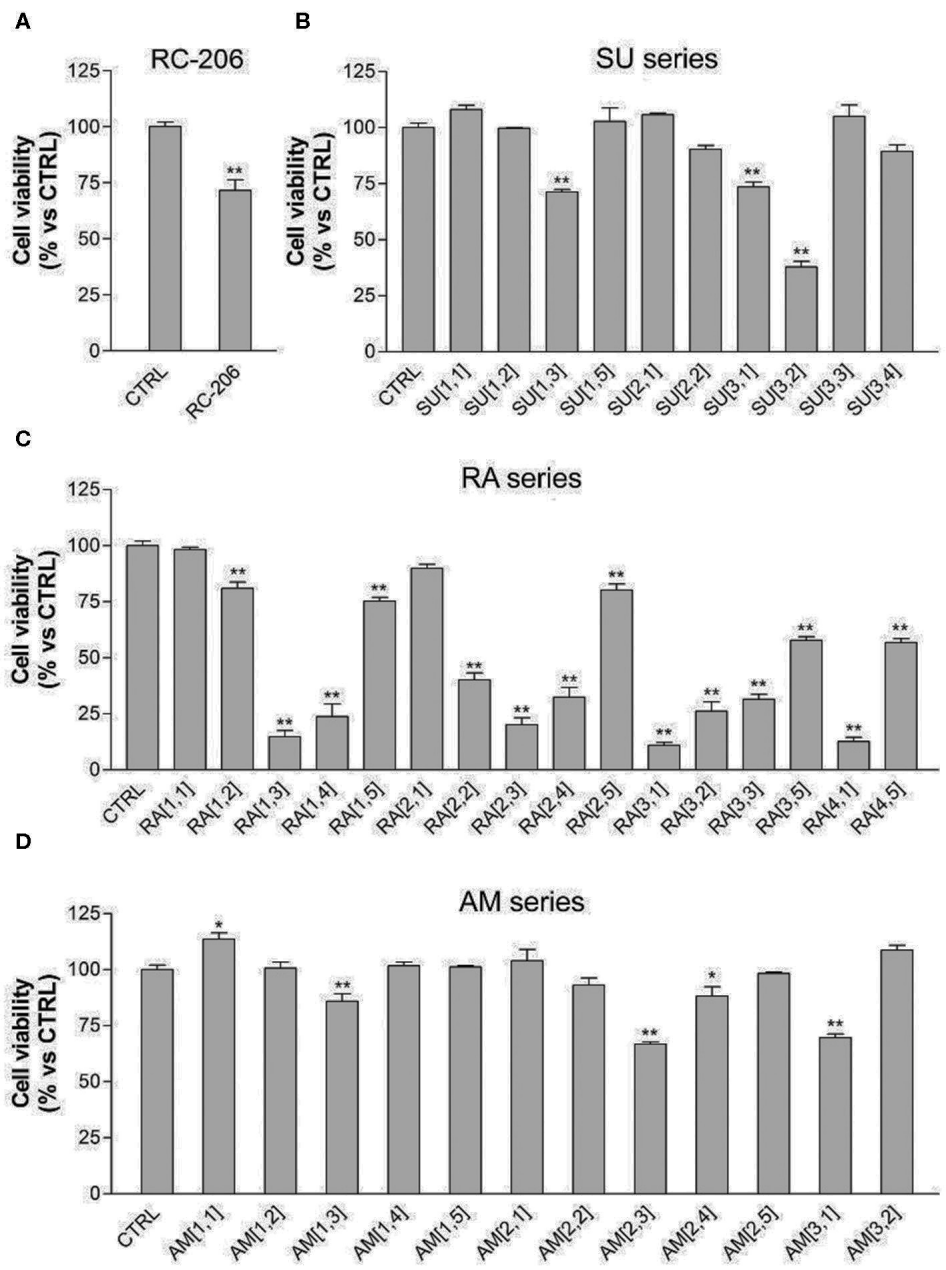
3-(4,5-Dimethylthiazol-2-yl)-2,5-diphenyltetrazolium bromide (MTT) assay, Roswell Park Memorial Institute (RPMI) 8226 cells treated with test compounds for 24 h at 40 μM concentration. Results are expressed as cell viability (%). **(A) RC-206**. **(B) SU** molecules. **(C) RA** molecules. **(D) AM** molecules. All graphs are represented as the mean percentage ± *SD* of three independent experiments and are compared to untreated controls (CTRL) arbitrarily set to 100%. *p < 0.05 vs. CTRL; **p < 0.01 vs. CTRL.

Regarding the effect of the novel compounds against RPMI 8226 cells, the cell viability was preliminarily assessed by MTT assay after 24 h of treatment, at the concentration of 40 μM, corresponding to the IC_50_ 24 h of **RC-106**. Results were compared to RPMI 8226 untreated control cells (Figure 4). **RC-206** resulted to be effective, thus confirming that the replacement of the piperidine moiety by the piperazine one does not greatly affect the antiproliferative activity (Figure 4A). Within the compound library, the most interesting compounds belong to **RA** series, followed by those of **SU** and **AM** series. Indeed, 9 out 19 of **RA** compounds, i.e., **RA**[1,3], **RA**[1,4], **RA**[2,2], **RA**[2,3], **RA**[2,4], **RA**[3,1], **RA**[3,2], **RA**[3,3], and **RA**[4,1], impaired cell viability in a more effective way than **RC**-**106** (Figure 4A). Only three compounds of the **SU** series significantly impaired cell viability of RPMI 8226 cells, i.e., **SU**[1,3], **SU**[3,1], and **SU**[3,2]. Of particular interest is the compound **SU**[3,2], which is able to induce a greater reduction in cell viability compared to **RC-106** effect (Figure 4C). Only four molecules of **AM** series (**AM**[1,3], **AM**[2,3], **AM**[2,4], and **AM**[3,1]) impaired cell viability of RPMI 8226 cells, but they are less effective than **RC-106**. Among all compounds tested, only **AM**[1,1], characterized by the presence of a cycloalkyl as R substituent, was found to induce a slight, but significant, increase in cell viability (Figure 4B), and for this reason, it must be discarded. Of note, none of the assayed compounds caused relevant changes in RPMI 8226 cell morphology. The IC_50_ of the 10 most effective compounds against RPMI 8226 cell viability were then calculated (Table 2).

**Table 2:** IC_50_ of Roswell Park Memorial Institute (RPMI) 8226 cell viability after treatment with the 10 most effective compounds.

	**IC_**50**_ (μm)**	***SD***
**RC-106**	40.07	2.34
**RC-206**	64.72	5.64
**RA**[1,3]	28.26**	2.37
**RA**[1,4]	32.34**	1.81
**RA**[2,2]	36.91	0.41
**RA**[2,3]	27.58**	1.46
**RA**[2,4]	34.42**	0.14
**RA**[3,1]	26.15**	0.23
**RA**[3,2]	33.86**	0.84
**RA**[3,3]	33.89**	0.37
**RA**[4,1]	27.11**	2.48
**SU**[3,2]	38.18	2.43

** *p < 0.01 vs. **RC-106***.

Results described so far clearly suggest that, against MM, the change in piperidine ring into piperazine is an allowed modification and that the “X” portion of the general structure reported in Figure 1 plays a key role in the activity: indeed, the optimal results were obtained for compounds belonging to **RA** series, i.e., when the linker between piperazine and the “R” substituent consists of a simple methylene. Finally, a small aryl group (i.e., phenyl, 4-methoxy-phenyl) is preferred when combined with cyclohexyl and *p*-substituted aromatics attached to the piperazine ring, whereas the bulkier naphthalene group is allowed when “R” consists of the small cyclopentyl and cyclohexyl ring (**RA**[3,1], **RA**[3,2], **RA**[4,1]). An exception is represented by **RA**[3,3].

**Figure 5:**
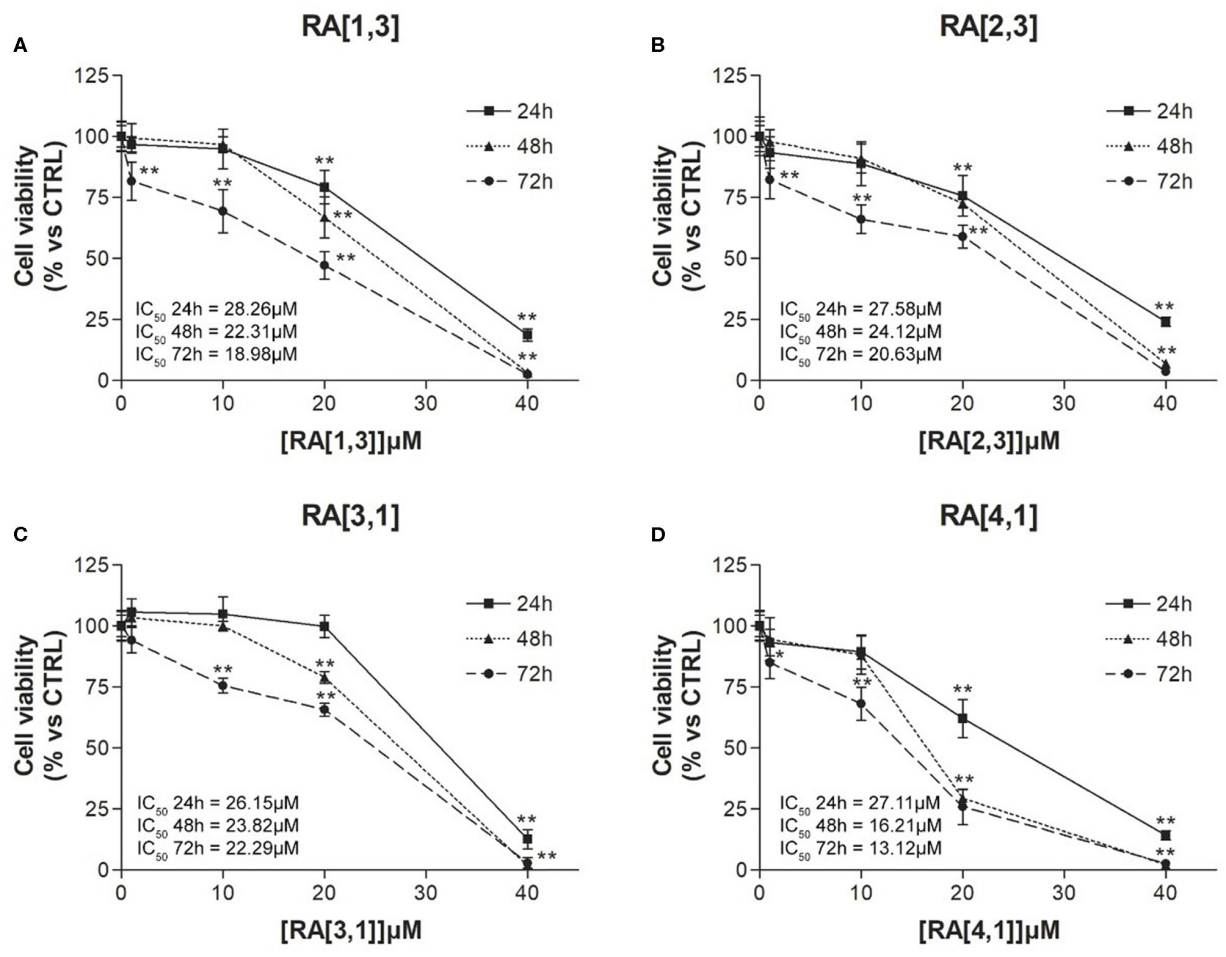
Cell viability (%) curves of Roswell Park Memorial Institute (RPMI) 8226 cells treated with **RA**[1,3], **RA**[2,3], **RA**[3,1], and **RA**[4,1] molecules. 3-(4,5-Dimethylthiazol-2-yl)-2,5-diphenyltetrazolium bromide (MTT) assay of RPMI 8226 cells treated with different concentration (1–40 μM) of **(A) RA**[1,3], **(B) RA**[2,3], **(C) RA**[3,1], and **(D) RA**[4,1] for 24, 48, and 72 h. All graphs are represented as the mean percentage ± *SD* of three independent experiments and are compared to untreated controls (CTRL) arbitrarily set to 100%. **p < 0.01 vs. CTRL.

Cell viability of the four most effective compound (**RA**[1,3], **RA**[2,3], **RA**[3,1], and **RA**[4,1]) was evaluated at different time points (24, 48, and 72 h) and different concentrations (1–40 μM) by MTT assay. All compounds were able to significantly reduce in a dose- and time-dependent manner the cell viability of RPMI 8226 cells (Figure 5).

**Figure 6:**
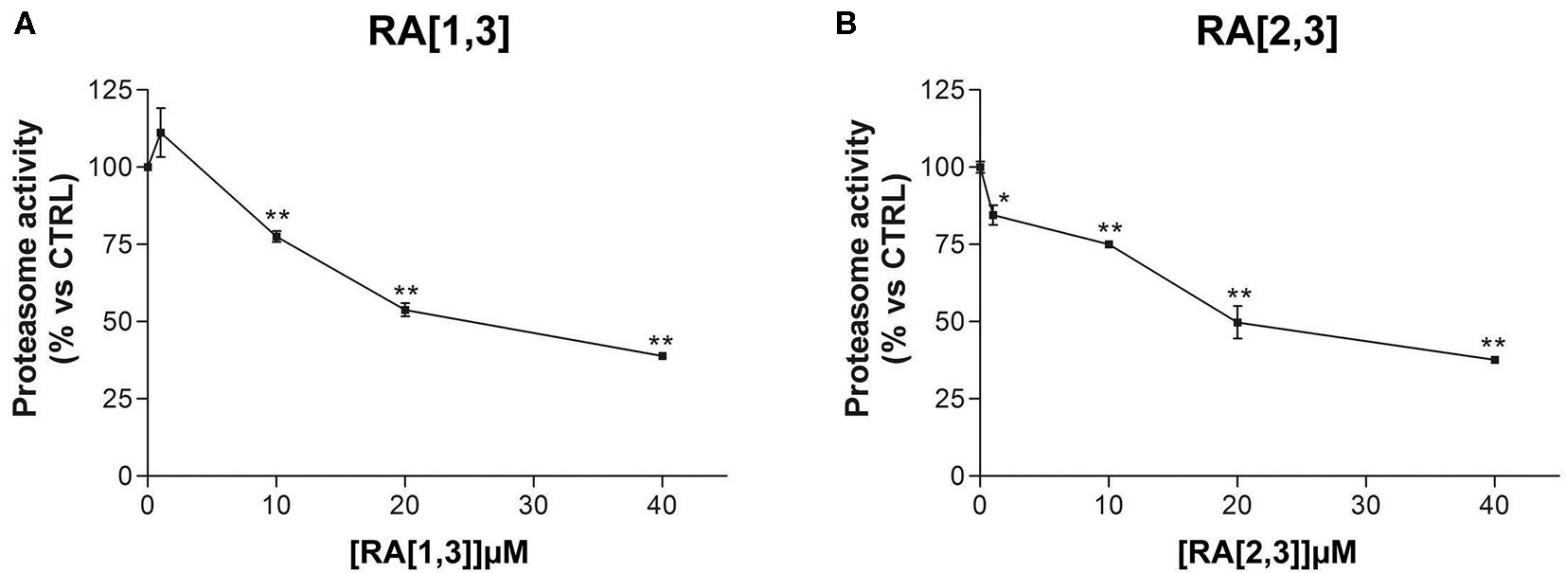
Proteasome activity of Roswell Park Memorial Institute (RPMI) 8226 cells after treatment with **RA**[1,3] and **RA**[2,3]. **(A)** Proteasome activity of RPMI 8226 cells after treatment with increasing concentrations of **RA**[1,3] (1–40 μM) for 24 h. **(B)** Proteasome activity of RPMI 8226 cells after treatment with increasing concentrations of **RA**[2,3] (1–40 μM) for 24 h. All graphs are represented as the mean percentage ± *SD* of three independent experiments, and results are compared to untreated control cells (CTRL) arbitrarily set to 100%. *p < 0.05 vs. CTRL; **p < 0.01 vs. CTRL.

**Figure 7:**
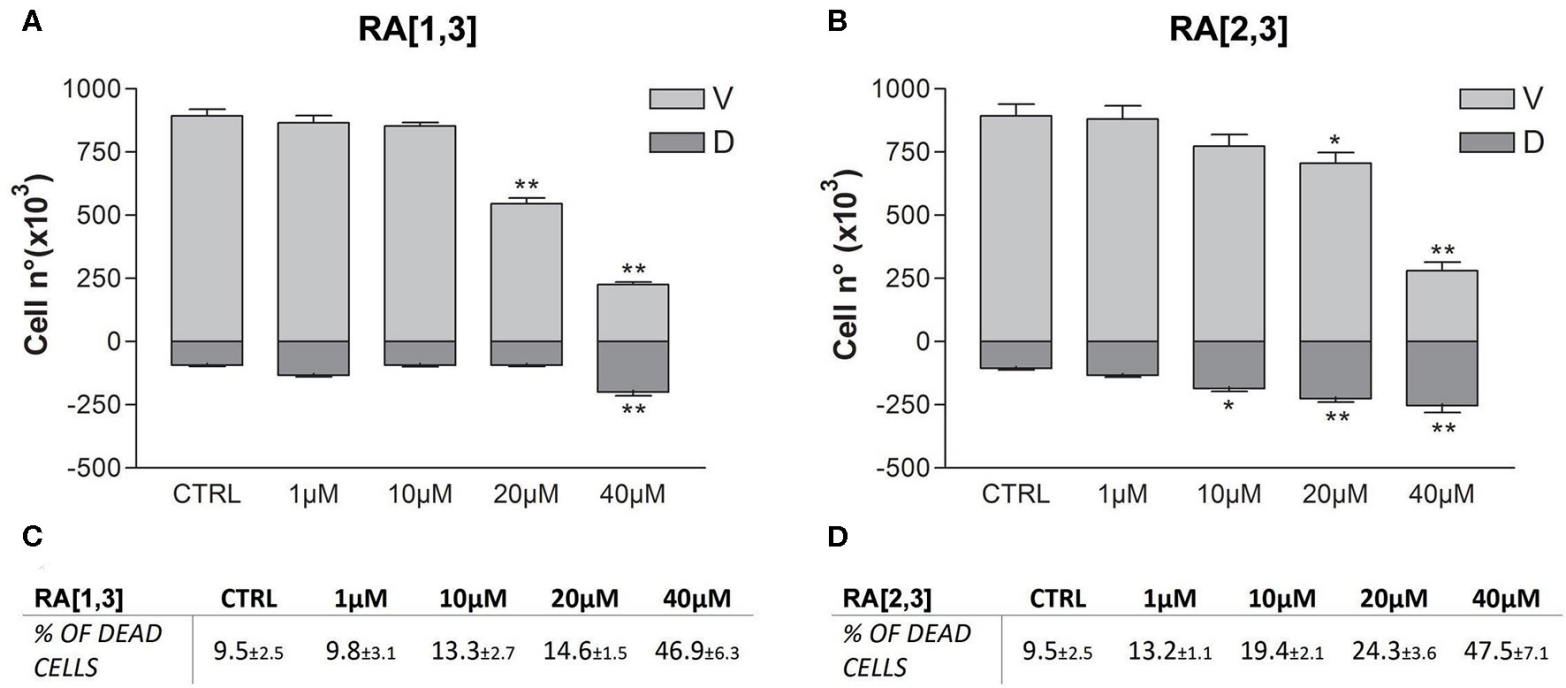
Trypan blue vital count of Roswell Park Memorial Institute (RPMI) 8226 cells treated with **RA**[1,3] and **RA**[2,3]. **(A)** Vital count of RPMI 8226 cells after treatment with increasing concentrations of **RA**[1,3] (1–40 μM) for 24 h. **(B)** Vital count of RPMI 8226 cells after treatment with increasing concentrations of **RA**[2,3] (1–40 μM) for 24 h. In graphs **(A,B)**, both viable (V) and dead (D) cells number are represented. **(C)** Table represents the percentage of dead cells compared to all counted cells after treatment with increasing concentrations of **RA**[1,3] (1–40 μM). **(D)** Table represents the percentage of dead cells compared to all counted cells after treatment with increasing concentrations of RA8 (1–40 μM). *p < 0.05 vs. CTRL; **p < 0.01 vs. CTRL.

Prompted by the positive results obtained, the proteasome activity of the compounds belonging to the RA series was also evaluated. After 24 h treatment of RPMI 8226 cells, **RA**[1,3] and **RA**[2,3] showed higher proteasome inhibition activity than **RC-106**. Both compounds significantly inhibited proteasome in a dose-dependent manner and showed an IC_50_ value, for the inhibition of proteasome, ~22 μM (versus an **RC-106** IC_50_ value of 35 μM) (Figure 6A). Moreover, both **RA**[1,3] and **RA**[2,3] reduced in a dose-dependent manner the number of viable cells counted by trypan blue vital count (Figure 7). However, the induction of cell death was quite different. After **RA**[1,3] 20 μM treatment, the percentage of RPMI 8226 dead cells were comparable to untreated controls, then at 40 μM concentration, a significant increase in cell mortality was observable. Instead, after **RA**[2,3] treatment, the number of RPMI 8226 dead cells increased in a dose-dependent manner. This could suggest a cytostatic effect of **RA**[1,3] and a cytotoxic effect of **RA**[2,3].

## Discussion

To identify novel chemical entities active against aggressive tumors with poor prognosis, i.e., GB and MM for which effective drugs or therapeutic strategies are still needed, we investigated the chemical space around compound **RC-106** (Rui et al., 2016b), characterized by a promising cytotoxic activity against glioblastoma (U87MG) and multiple myeloma (RPMI-8226) cell lines. To determine which positions are amenable to the lead optimization extension strategy and to find effective developable compounds, we designed a small compounds library, replacing the piperidine moiety of **RC-106** with the piperazine ring, thus gaining a third point of derivatization. Moreover, the derivatization of piperazine allows the attachment of a wide range of alkyl or aryl substituents through a combinatorial approach. At first, we synthesized compound **RC-206**, replacing the piperidine moiety with piperazine nucleus, and evaluated its antiproliferative properties against GB and MM. *In vitro* results showed that such a minor structural change is allowed. Therefore, we built a compound library on the new scaffold, taking into account the synthetic feasibility and commercial availability of building blocks. In detail, we choose phenyl, 4-methoxy-phenyl, naphthyl, and 6-methoxy-naphthyl scaffolds for the exploration of stereoelectronic features of the primary aryl group, and we envisaged to adopt three different approaches for piperidine derivatization: (i) sulfonylation (**SU**), (ii) reductive amination and (**RA**), and (iii) amidation (**AM**). As a result, a compound library of 60 members was designed. Since it is well known that pharmacokinetic studies performed in the early stages of drug discovery may reduce attrition rate (Kola and Landis, 2004; Merlot, 2010), we evaluated the *in silico* ADME profile and drug-likeness of the compounds. In particular, the prediction of log *P* and log *S*-values indicates that the majority of designed compounds is endowed with an improved water-solubility with respect to *hit* compound **RC-106**. Only a few compounds have a predicted solubility profile comparable to that of **RC-106**. In addition, good GI and BBB permeability were predicted, and no PAINS structures were identified.

Only a few compounds have a predicted solubility profile comparable to that of **RC-106**. In addition, good GI and BBB permeability were predicted, and no PAINS structures were identified. Considering the results obtained for the whole library, in terms of solubility and barriers permeability, we moved forward to synthesis and experimental evaluation of the designed compounds. The synthetic strategy previously reported for **RC-106** (Rui et al., 2016b) was successfully optimized and adapted to obtain a library of structurally diverse analogs. All the designed compounds, with few exceptions, were obtained in suitable amounts and purity for biological investigation. Moreover, the adopted approach could be exploited for the synthesis of other **RC-106** analogs to further expand the exploration of chemical space around our *hit* compound. All compounds have been evaluated in two different human cancer cell lines, U87-MG glioblastoma cells and RPMI 8226 multiple myeloma cells, and screened their ability in inhibiting or hampering the tumor growth. In general, new compounds showed poor or no effect against glioblastoma U87-MG cell line, whereas nine molecules of **RA** series and one of **SU** series were significantly more effective in reducing RPMI 8226 cells viability than **RC-106**. In particular, compounds **RA**[1,3], **RA** [2,3], **RA** [3,1], and **RA** [4,1] deserved to be mentioned: they showed a good dose–response curve by MTT assay (IC_50_ values <30 μM) and therefore the capability to significantly slow the metabolic activity of tumor cells after only 24 h exposure. Such effect was mirrored by the impairment of proteasome activity (Figure 6), an enzyme involved in the protein homeostasis. This activity was also documented in our previous work focused on the antitumor activity of the *hit* **RC-106** and where we showed its capability to trigger the UPR response machinery (Tesei et al., 2019). Accordingly, the compounds also hamper tumor cell growth starting from the lowest concentrations tested, as evidenced by the proliferation test (Figure 7) showing also a significant induction of cell death (~50%) at the highest concentration tested (Figure 5).

For U87-MG, none of the new molecules of **RA**, **AM**, and **SU** series was able to reduce cell viability of U87-MG after 24 h of treatment. Only **RC-206** showed a cytotoxic activity higher than the *hit* compound, thus representing the most promising molecules against U87-MG cells. However, of particular interest is molecule **AM**[3,1] that even if it was not able to reduce U87-MG cell viability after 24 h of treatment, it caused evident alteration of cell morphology. U87-MG cells lost the elongated cell body with long processes and acquired an epithelial-like phenotype with a flat cell body without processes and presence of tight junctions between cells. These results suggest that the process inducing a reduction in U87-MG cell viability triggered by **AM**[3,1], evident only after 48 and 72 h of treatment, starts already from 24 h preceded by specific morphological changes caused by **AM**[3,1]. Such morphology alteration strongly suggests a transition of U87-MG cell to an epithelial cell morphology (MET). Cellular morphological changes are evident in epithelial–mesenchymal transition (EMT) and in the reverse process mesenchymal–epithelial transition (MET) and are reported in previous works on U87-MG cells (Guan et al., 2017; Yang et al., 2020, p. 3). EMT and MET play an important role in development, reprogramming, and tumorigenesis (Chen T. et al., 2017; Pei et al., 2019). Different compounds are able to exert antitumoral effects reversing ETM and promoting MET (Peng et al., 2016; Cheng et al., 2018; Li et al., 2019; Yuan et al., 2019). In addition, a cocktail of reprogramming transcription factors is able to promote MET attenuating the malignancy of cancer cells (Takaishi et al., 2016). It is worth noting that glioblastoma is the most aggressive (WHO grade IV) and common of the human gliomas. The invasive characteristic of glioblastoma, at least in part, is due to their high migratory potential to invade the surrounding tissue. EMT has been pointed as one of the mechanisms that confer to GBM cells this invasive property (Iser et al., 2017). Therefore, transition to polarized epithelial cells (MET) increases efficacy of antineoplastic agents and makes tumors less aggressive and with a better patient prognosis (Takaishi et al., 2016). Further experiments will be necessary to demonstrate the property of **AM**[3,1] to induce the MET transition of U87-MG cells and confirm our hypothesis.

## Conclusions

To conclude, exploration of chemical space around our previously reported *hit* **RC-106** led us to identify compounds endowed with interesting antitumor properties. In particular, molecules derived from **RA-106** have different effects on RPMI 8226 and U87-MG cell lines, resulting in RPMI 8226 myeloma cells more sensitive than glioblastoma U87-MG cell line. These differences could be due to different mechanisms of action of the molecules in the cell lines used for biological testing. It is not surprising, since the compound may have different effects depending on the tumoral cell line examined (Bittkau et al., 2019; Chen et al., 2019) and on the concentrations evaluated (Jiménez-Orozco et al., 2011; Navarro-Villarán et al., 2016).

**Figure 8:**
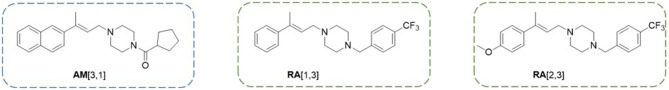
Structures of the three compounds emerged as the most interesting for their promising anticancer profile.

Regarding compounds effective against RPMI 8226 cells, of particular interest are **RA**[1,3] and **RA**[2,3] (Figure 8), able to significantly inhibit proteasome in a dose-dependent manner. Indeed, proteasome inhibitors, like bortezomib (BTZ), ixazomib, and carfilzomib, are currently used for the treatment of multiple myeloma (Cvek and Dvorak, 2008; Chen et al., 2011; Chen T. et al., 2017; Okazuka and Ishida, 2018; Kim et al., 2019). Conversely, **AM**[3,1] (Figure 8) emerged for its activity on U87-MG cell lines since it is able to induce a significant cellular morphology alteration and a time-dependent effect on cell viability.

In view of the results discussed so far, the biological profile of **RA**[1,3] and **RA**[2,3] and **AM**[3,1] will be deepened. Studies of mechanism of action and of the potential in improving the efficacy of cancer treatment and reducing the side effects are ongoing and will be presented in due course.

## Data Availability Statement

The raw data supporting the conclusions of this article will be made available by the authors, without undue reservation, to any qualified researcher.

## Author Contributions

All authors listed have made a substantial, direct and intellectual contribution to the work, and approved it for publication.

## Conflict of Interest

SS performed part of her Ph.D. project in the company Taros Chemicals GmbH and Co. KG, working on a different topic. The remaining authors declare that the research was conducted in the absence of any commercial or financial relationships that could be construed as a potential conflict of interest.

## Footnotes

^1^Cancer. n.d. Available online at: https://www.who.int/news-room/fact-sheets/detail/cancer (accessed February 7, 2020).

^2^Silicos-It Filter-ItTM. n.d. Available online at: http://silicos-it.be.s3-website-eu-west-1.amazonaws.com/software/filter-it/1.0.2/filter-it.html (accessed March 16, 2020).
