# Supplementary material for: Exploring the RC-106 Chemical Space: Design and Synthesis of Novel (E)-1-(3-Arylbut-2-en-1-yl)-4-(Substituted) Piperazine Derivatives as Potential Anticancer Agents
Source: Front Chem. 2020 Jun 30;8:495. doi: 10.3389/fchem.2020.00495 (PMC7338850; doi:10.3389/fchem.2020.00495)
Supplement: Supplementary file 1 [file Table_1.DOCX]

Supplementary Material

# SwissADME predictions

**Supplementary Table 1** and **Supplementary Table 1** report data obtained from SwissADME predictions on the whole library computed as both neutral and protonated species, respectively. For a complete description on computational methods implemented in SwissADME, please refer to Daina, A. et al. *Sci Rep* **7,**42717 (2017). <https://doi.org/10.1038/srep42717>.

# ^1^H and ^13^C NMR spectra

Herein are reported ^1^H and ^13^C NMR of the key intermediates and those of some representative compounds. ^1^H NMR spectra shown here were recorded at room temperature on a Bruker Avance spectrometer operating at 300 MHz. Proton decoupled ^13^C NMR data were acquired at 100 MHz.

| **Supporting Table 1.** Results of the predictions obtained with the SwissADME web tool. The designed compounds are computed as neutral species. | | | | | | | | | | | | | | | | | | | | | | | | | | |
| --- | --- | --- | --- | --- | --- | --- | --- | --- | --- | --- | --- | --- | --- | --- | --- | --- | --- | --- | --- | --- | --- | --- | --- | --- | --- | --- |
| **Name** | **TPSA** | **iLOGP** | **XLOGP3** | **WLOGP** | **MLOGP** | **Silicos-IT Log P** | **Consensus Log P** | **ESOL Log S** | **ESOL Solubility (mol/l)** | **Ali**  **Log S** | **Ali Solubility (mol/l)** | **Silicos-IT LogSw** | **Silicos-IT Solubility (mol/l)** | **GI**  **abs.** | **BBB permeant** | **Pgp substrate** | **CYP1A2 inhibitor** | **CYP2C19 inhibitor** | **CYP2C9 inhibitor** | **CYP2D6 inhibitor** | **CYP3A4 inhibitor** | **log Kp (cm/s)** | **Lipinski #violations** | **PAINS #alerts** | **Leadlikeness #violations** | **Synthetic Accessibility** |
| **RC-106** | 3.24 | 4.42 | 6.98 | 5.82 | 5.4 | 6.27 | 5.78 | -6.6 | 2.82E-07 | -6.86 | 1.37E-07 | -8.33 | 4.71E-09 | Low | No | Yes | Yes | No | No | Yes | No | -3.51 | 1 | 0 | 2 | 3.31 |
| **RC-206** | 6.48 | 4.32 | 5.58 | 4.15 | 4.27 | 5.14 | 4.69 | -5.7 | 2.12E-06 | -5.48 | 3.33E-06 | -7.61 | 2.43E-08 | High | Yes | Yes | No | No | No | Yes | No | -4.51 | 1 | 0 | 2 | 2.91 |
| **RA[1,1]** | 6.48 | 4.2 | 4.66 | 3.14 | 3.64 | 3.96 | 3.92 | -4.5 | 3.18E-05 | -4.52 | 3.00E-05 | -4.61 | 2.44E-05 | High | Yes | No | No | No | No | Yes | No | -4.81 | 0 | 0 | 1 | 2.78 |
| **RA[1,2]** | 6.48 | 4.36 | 5.21 | 3.53 | 3.86 | 4.2 | 4.23 | -4.9 | 1.19E-05 | -5.09 | 8.06E-06 | -4.88 | 1.30E-05 | High | Yes | No | No | No | No | Yes | No | -4.51 | 0 | 0 | 1 | 2.91 |
| **RA[1,3]** | 6.48 | 4.16 | 5.21 | 5.17 | 4.42 | 5.15 | 4.82 | -5.4 | 4.20E-06 | -5.09 | 8.06E-06 | -6.81 | 1.54E-07 | High | No | No | Yes | No | No | Yes | No | -4.88 | 1 | 0 | 2 | 2.91 |
| **RA[1,4]** | 6.48 | 4.19 | 4.69 | 3.3 | 3.83 | 4.62 | 4.13 | -4.8 | 1.51E-05 | -4.55 | 2.79E-05 | -6.35 | 4.49E-07 | High | Yes | No | No | No | No | Yes | No | -4.92 | 0 | 0 | 1 | 2.78 |
| **RA[1,5]** | 6.48 | 4.03 | 4.53 | 4.11 | 4.36 | 4.92 | 4.39 | -4.8 | 1.44E-05 | -4.39 | 4.09E-05 | -6.51 | 3.11E-07 | High | Yes | No | Yes | No | No | Yes | No | -5.17 | 1 | 0 | 1 | 2.85 |
| **RA[2,1]** | 15.71 | 4.47 | 4.64 | 3.14 | 3.25 | 4 | 3.9 | -4.6 | 2.58E-05 | -4.7 | 2.01E-05 | -4.72 | 1.88E-05 | High | Yes | No | No | No | No | Yes | No | -5.01 | 0 | 0 | 1 | 2.87 |
| **RA[2,2]** | 15.71 | 4.63 | 5.18 | 3.53 | 3.47 | 4.25 | 4.21 | -5 | 9.80E-06 | -5.26 | 5.54E-06 | -5 | 1.01E-05 | High | Yes | No | No | No | No | Yes | No | -4.71 | 0 | 0 | 1 | 3 |
| **RA[2,3]** | 15.71 | 4.55 | 5.19 | 5.17 | 4.02 | 5.22 | 4.83 | -5.5 | 3.45E-06 | -5.27 | 5.41E-06 | -6.92 | 1.22E-07 | High | Yes | No | Yes | No | No | Yes | No | -5.08 | 0 | 0 | 2 | 2.99 |
| **RA[2,4]** | 15.71 | 4.35 | 4.67 | 3.31 | 3.43 | 4.67 | 4.09 | -4.9 | 1.26E-05 | -4.73 | 1.87E-05 | -6.46 | 3.48E-07 | High | Yes | No | No | No | No | Yes | No | -5.12 | 0 | 0 | 2 | 2.88 |
| **RA[2,5]** | 15.71 | 4.26 | 4.5 | 4.12 | 3.97 | 4.98 | 4.37 | -4.9 | 1.21E-05 | -4.55 | 2.81E-05 | -6.61 | 2.44E-07 | High | Yes | No | No | Yes | No | Yes | No | -5.38 | 0 | 0 | 2 | 2.92 |
| **RA[3,1]** | 6.48 | 4.62 | 5.91 | 4.29 | 4.29 | 5 | 4.82 | -5.7 | 2.10E-06 | -5.82 | 1.51E-06 | -6.26 | 5.50E-07 | High | Yes | Yes | Yes | No | No | Yes | No | -4.23 | 1 | 0 | 1 | 3.02 |
| **RA[3,2]** | 6.48 | 4.73 | 6.46 | 4.68 | 4.5 | 5.24 | 5.12 | -6.1 | 7.91E-07 | -6.39 | 4.07E-07 | -6.53 | 2.96E-07 | High | Yes | Yes | Yes | No | No | Yes | No | -3.92 | 1 | 0 | 2 | 3.16 |
| **RA[3,3]** | 6.48 | 4.57 | 6.46 | 6.32 | 5.05 | 6.2 | 5.72 | -6.5 | 2.97E-07 | -6.39 | 4.07E-07 | -8.44 | 3.60E-09 | Low | No | Yes | No | No | No | Yes | No | -4.3 | 1 | 0 | 2 | 3.15 |
| **RA[3,4]** | 6.48 | 4.56 | 5.94 | 4.46 | 4.48 | 5.66 | 5.02 | -6 | 1.07E-06 | -5.85 | 1.41E-06 | -7.99 | 1.02E-08 | High | Yes | Yes | No | No | No | Yes | No | -4.34 | 1 | 0 | 2 | 3.03 |
| **RA[3,5]** | 6.48 | 4.53 | 5.78 | 5.27 | 5.01 | 5.97 | 5.31 | -6 | 1.02E-06 | -5.69 | 2.06E-06 | -8.14 | 7.16E-09 | High | No | Yes | No | No | No | Yes | No | -4.59 | 1 | 0 | 2 | 3.09 |
| **RA[4,1]** | 15.71 | 4.88 | 5.89 | 4.3 | 3.89 | 5.05 | 4.8 | -5.8 | 1.71E-06 | -5.99 | 1.02E-06 | -6.37 | 4.31E-07 | High | Yes | Yes | Yes | No | No | Yes | No | -4.43 | 0 | 0 | 2 | 3.09 |
| **RA[4,2]** | 15.71 | 5.04 | 6.43 | 4.69 | 4.09 | 5.3 | 5.11 | -6.18 | 6.55E-07 | -6.55 | 2.80E-07 | -6.63 | 2.33E-07 | High | Yes | Yes | Yes | No | No | Yes | No | -4.13 | 0 | 0 | 2 | 3.23 |
| **RA[4,3]** | 15.71 | 4.86 | 6.44 | 6.33 | 4.63 | 6.27 | 5.71 | -6.6 | 2.44E-07 | -6.56 | 2.73E-07 | -8.54 | 2.86E-09 | Low | No | Yes | No | No | No | Yes | No | -4.5 | 1 | 0 | 2 | 3.21 |
| **RA[4,4]** | 15.71 | 4.91 | 5.92 | 4.46 | 4.07 | 5.72 | 5.02 | -6.05 | 8.88E-07 | -6.02 | 9.46E-07 | -8.09 | 8.05E-09 | High | Yes | Yes | No | No | No | Yes | No | -4.54 | 0 | 0 | 2 | 3.09 |
| **RA[4,5]** | 15.71 | 4.77 | 5.75 | 5.27 | 4.6 | 6.03 | 5.29 | -6.1 | 8.55E-07 | -5.85 | 1.42E-06 | -8.25 | 5.68E-09 | High | Yes | Yes | No | No | No | Yes | No | -4.79 | 1 | 0 | 2 | 3.14 |
| **AM[1,1]** | 23.55 | 3.68 | 3.84 | 2.66 | 3.08 | 3.49 | 3.35 | -4.06 | 8.72E-05 | -4.03 | 9.32E-05 | -4.15 | 7.08E-05 | High | Yes | No | No | Yes | No | Yes | No | -5.48 | 0 | 0 | 1 | 2.68 |
| **AM[1,2]** | 23.55 | 3.91 | 4.38 | 3.05 | 3.3 | 3.73 | 3.67 | -4.48 | 3.32E-05 | -4.59 | 2.57E-05 | -4.42 | 3.79E-05 | High | Yes | No | No | Yes | No | Yes | No | -5.18 | 0 | 0 | 1 | 2.81 |
| **AM[1,3]** | 23.55 | 3.89 | 4.84 | 4.96 | 4.13 | 4.69 | 4.5 | -5.22 | 6.05E-06 | -5.07 | 8.55E-06 | -6.34 | 4.52E-07 | High | Yes | No | No | Yes | No | Yes | No | -5.23 | 0 | 0 | 2 | 2.79 |
| **AM[1,4]** | 23.55 | 3.78 | 4.32 | 3.09 | 3.55 | 4.15 | 3.78 | -4.66 | 2.19E-05 | -4.53 | 2.96E-05 | -5.88 | 1.31E-06 | High | Yes | No | No | Yes | Yes | Yes | No | -5.27 | 0 | 0 | 1 | 2.68 |
| **AM[1,5]** | 23.55 | 3.59 | 4.16 | 3.9 | 4.08 | 4.46 | 4.04 | -4.68 | 2.08E-05 | -4.36 | 4.34E-05 | -6.04 | 9.10E-07 | High | Yes | No | No | Yes | No | Yes | No | -5.52 | 0 | 0 | 2 | 2.74 |
| **AM[2,1]** | 32.78 | 4.1 | 3.81 | 2.67 | 2.72 | 3.54 | 3.37 | -4.15 | 7.16E-05 | -4.19 | 6.41E-05 | -4.26 | 5.50E-05 | High | Yes | No | No | No | No | Yes | No | -5.68 | 0 | 0 | 1 | 2.79 |
| **AM[2,2]** | 32.78 | 4.16 | 4.35 | 3.06 | 2.93 | 3.79 | 3.66 | -4.57 | 2.72E-05 | -4.75 | 1.76E-05 | -4.53 | 2.95E-05 | High | Yes | No | No | No | No | Yes | No | -5.39 | 0 | 0 | 2 | 2.92 |
| **AM[2,3]** | 32.78 | 4.12 | 4.81 | 4.97 | 3.76 | 4.77 | 4.49 | -5.3 | 5.03E-06 | -5.23 | 5.88E-06 | -6.45 | 3.58E-07 | High | Yes | No | No | Yes | No | Yes | Yes | -5.44 | 0 | 0 | 2 | 2.9 |
| **AM[2,4]** | 32.78 | 4.04 | 4.29 | 3.1 | 3.18 | 4.21 | 3.77 | -4.74 | 1.84E-05 | -4.69 | 2.04E-05 | -5.99 | 1.02E-06 | High | Yes | No | No | Yes | Yes | Yes | Yes | -5.48 | 0 | 0 | 2 | 2.8 |
| **AM[2,5]** | 32.78 | 3.86 | 4.13 | 3.91 | 3.71 | 4.53 | 4.03 | -4.76 | 1.74E-05 | -4.53 | 2.98E-05 | -6.15 | 7.14E-07 | High | Yes | No | No | Yes | Yes | Yes | No | -5.72 | 0 | 0 | 2 | 2.84 |
| **AM[3,1]** | 23.55 | 4.1 | 5.09 | 3.82 | 3.74 | 4.54 | 4.26 | -5.24 | 5.78E-06 | -5.33 | 4.70E-06 | -5.79 | 1.61E-06 | High | Yes | Yes | Yes | Yes | No | Yes | Yes | -4.9 | 0 | 0 | 2 | 2.93 |
| **AM[3,2]** | 23.55 | 4.25 | 5.63 | 4.21 | 3.95 | 4.78 | 4.56 | -5.66 | 2.21E-06 | -5.89 | 1.29E-06 | -6.06 | 8.65E-07 | High | Yes | Yes | Yes | No | No | Yes | Yes | -4.6 | 0 | 0 | 2 | 3.06 |
| **AM[3,3]** | 23.55 | 4.19 | 6.09 | 6.11 | 4.77 | 5.74 | 5.38 | -6.37 | 4.27E-07 | -6.37 | 4.31E-07 | -7.97 | 1.06E-08 | High | No | Yes | No | Yes | No | Yes | No | -4.65 | 1 | 0 | 2 | 3.04 |
| **AM[3,4]** | 23.55 | 4.23 | 5.57 | 4.25 | 4.2 | 5.2 | 4.69 | -5.81 | 1.54E-06 | -5.83 | 1.49E-06 | -7.52 | 2.99E-08 | High | Yes | No | No | Yes | Yes | Yes | Yes | -4.69 | 1 | 0 | 2 | 2.92 |
| **AM[3,5]** | 23.55 | 4.06 | 5.4 | 5.06 | 4.73 | 5.51 | 4.95 | -5.83 | 1.49E-06 | -5.65 | 2.24E-06 | -7.68 | 2.10E-08 | High | Yes | No | No | Yes | Yes | Yes | No | -4.95 | 1 | 0 | 2 | 2.98 |
| **AM[4,1]** | 32.78 | 4.36 | 5.06 | 3.82 | 3.37 | 4.59 | 4.24 | -5.32 | 4.78E-06 | -5.49 | 3.23E-06 | -5.9 | 1.26E-06 | High | Yes | Yes | No | No | No | Yes | Yes | -5.1 | 0 | 0 | 2 | 3.01 |
| **AM[4,2]** | 32.78 | 4.61 | 5.6 | 4.21 | 3.57 | 4.83 | 4.57 | -5.74 | 1.82E-06 | -6.05 | 8.90E-07 | -6.17 | 6.82E-07 | High | Yes | Yes | No | No | No | Yes | Yes | -4.8 | 0 | 0 | 2 | 3.15 |
| **AM[4,3]** | 32.78 | 4.62 | 6.06 | 6.12 | 4.38 | 5.82 | 5.4 | -6.45 | 3.56E-07 | -6.53 | 2.96E-07 | -8.07 | 8.47E-09 | High | No | Yes | No | No | No | Yes | No | -4.86 | 1 | 0 | 2 | 3.12 |
| **AM[4,4]** | 32.78 | 4.54 | 5.54 | 4.26 | 3.82 | 5.26 | 4.68 | -5.89 | 1.30E-06 | -5.99 | 1.03E-06 | -7.63 | 2.37E-08 | High | Yes | Yes | No | Yes | Yes | Yes | Yes | -4.9 | 0 | 0 | 2 | 3.01 |
| **AM[4,5]** | 32.78 | 4.31 | 5.38 | 5.07 | 4.35 | 5.58 | 4.94 | -5.91 | 1.23E-06 | -5.82 | 1.51E-06 | -7.78 | 1.67E-08 | High | Yes | Yes | No | Yes | Yes | Yes | No | -5.14 | 1 | 0 | 2 | 3.05 |
| **SU[1,1]** | 49 | 3.41 | 3.35 | 3.3 | 2.32 | 2.38 | 2.95 | -3.97 | 1.08E-04 | -4.06 | 8.78E-05 | -4.11 | 7.85E-05 | High | Yes | No | No | Yes | No | Yes | Yes | -6.05 | 0 | 0 | 0 | 3.45 |
| **SU[1,2]** | 49 | 3.66 | 3.89 | 3.69 | 2.55 | 2.62 | 3.28 | -4.39 | 4.11E-05 | -4.62 | 2.42E-05 | -4.37 | 4.23E-05 | High | Yes | No | No | Yes | Yes | Yes | Yes | -5.75 | 0 | 0 | 2 | 3.56 |
| **SU[1,3]** | 49 | 3.85 | 4.53 | 5.59 | 3.4 | 3.6 | 4.19 | -5.24 | 5.81E-06 | -5.28 | 5.24E-06 | -6.29 | 5.14E-07 | High | Yes | No | No | Yes | Yes | Yes | Yes | -5.67 | 0 | 0 | 2 | 3.31 |
| **SU[1,4]** | 49 | 3.48 | 4.01 | 3.72 | 2.81 | 3.04 | 3.41 | -4.67 | 2.11E-05 | -4.74 | 1.81E-05 | -5.84 | 1.46E-06 | High | Yes | No | No | Yes | Yes | Yes | Yes | -5.71 | 0 | 0 | 2 | 3.31 |
| **SU[1,5]** | 49 | 3.45 | 3.84 | 4.53 | 3.34 | 3.37 | 3.71 | -4.69 | 2.04E-05 | -4.56 | 2.72E-05 | -5.99 | 1.02E-06 | High | Yes | No | No | Yes | Yes | Yes | Yes | -5.97 | 0 | 0 | 2 | 3.25 |
| **SU[2,1]** | 58.23 | 3.84 | 3.32 | 3.31 | 1.99 | 2.44 | 2.98 | -4.05 | 8.85E-05 | -4.22 | 6.04E-05 | -4.21 | 6.15E-05 | High | Yes | No | No | Yes | No | Yes | Yes | -6.25 | 0 | 0 | 1 | 3.46 |
| **SU[2,2]** | 58.23 | 3.95 | 3.87 | 3.7 | 2.21 | 2.68 | 3.28 | -4.48 | 3.31E-05 | -4.79 | 1.62E-05 | -4.48 | 3.32E-05 | High | Yes | No | No | No | Yes | Yes | Yes | -5.95 | 0 | 0 | 2 | 3.58 |
| **SU[2,3]** | 58.23 | 4.06 | 4.5 | 5.6 | 3.05 | 3.67 | 4.18 | -5.32 | 4.82E-06 | -5.44 | 3.60E-06 | -6.39 | 4.09E-07 | High | No | No | No | Yes | Yes | Yes | Yes | -5.88 | 0 | 0 | 2 | 3.36 |
| **SU[2,4]** | 58.23 | 4.06 | 3.98 | 3.73 | 2.46 | 3.11 | 3.47 | -4.75 | 1.77E-05 | -4.9 | 1.25E-05 | -5.94 | 1.15E-06 | High | Yes | No | No | Yes | Yes | Yes | Yes | -5.92 | 0 | 0 | 2 | 3.35 |
| **SU[2,5]** | 49 | 3.93 | 4.6 | 4.45 | 3.01 | 3.42 | 3.88 | -5.14 | 7.19E-06 | -5.35 | 4.43E-06 | -5.74 | 1.81E-06 | High | Yes | No | Yes | Yes | Yes | Yes | Yes | -5.47 | 0 | 0 | 2 | 3.68 |
| **SU[3,1]** | 49 | 4.06 | 5.14 | 4.84 | 3.22 | 3.66 | 4.19 | -5.56 | 2.75E-06 | -5.91 | 1.22E-06 | -6.01 | 9.79E-07 | High | Yes | No | Yes | No | Yes | Yes | Yes | -5.17 | 0 | 0 | 2 | 3.8 |
| **SU[3,2]** | 49 | 4.17 | 5.78 | 6.74 | 4.05 | 4.64 | 5.08 | -6.39 | 4.11E-07 | -6.58 | 2.64E-07 | -7.92 | 1.22E-08 | High | No | No | No | Yes | Yes | Yes | Yes | -5.09 | 0 | 0 | 2 | 3.55 |
| **SU[3,3]** | 49 | 4.17 | 5.78 | 6.74 | 4.05 | 4.64 | 5.08 | -6.39 | 4.11E-07 | -6.58 | 2.64E-07 | -7.92 | 1.22E-08 | High | No | No | No | Yes | Yes | Yes | Yes | -5.09 | 0 | 0 | 2 | 3.55 |
| **SU[3,4]** | 49 | 4.26 | 5.26 | 4.88 | 3.48 | 4.09 | 4.39 | -5.83 | 1.49E-06 | -6.04 | 9.15E-07 | -7.47 | 3.40E-08 | High | Yes | No | No | No | Yes | Yes | Yes | -5.13 | 0 | 0 | 2 | 3.54 |
| **SU[3,5]** | 49 | 4.26 | 5.26 | 4.88 | 3.48 | 4.09 | 4.39 | -5.83 | 1.49E-06 | -6.04 | 9.15E-07 | -7.47 | 3.40E-08 | High | Yes | No | No | No | Yes | Yes | Yes | -5.13 | 0 | 0 | 2 | 3.54 |
| **SU[4,1]** | 58.23 | 4.21 | 4.57 | 4.46 | 2.66 | 3.49 | 3.88 | -5.23 | 5.93E-06 | -5.52 | 3.05E-06 | -5.84 | 1.44E-06 | High | Yes | No | No | Yes | Yes | Yes | Yes | -5.67 | 0 | 0 | 2 | 3.7 |
| **SU[4,2]** | 58.23 | 4.38 | 5.12 | 4.85 | 2.87 | 3.73 | 4.19 | -5.65 | 2.23E-06 | -6.09 | 8.18E-07 | -6.11 | 7.80E-07 | High | Yes | No | No | No | Yes | Yes | Yes | -5.36 | 0 | 0 | 2 | 3.82 |
| **SU[4,3]** | 58.23 | 4.48 | 5.75 | 6.75 | 3.69 | 4.72 | 5.08 | -6.47 | 3.41E-07 | -6.74 | 1.82E-07 | -8.01 | 9.79E-09 | High | No | No | No | Yes | Yes | Yes | Yes | -5.3 | 1 | 0 | 2 | 3.6 |
| **SU[4,4]** | 58.23 | 4.47 | 5.23 | 4.89 | 3.12 | 4.16 | 4.37 | -5.9 | 1.25E-06 | -6.2 | 6.29E-07 | -7.57 | 2.70E-08 | High | Yes | No | No | Yes | Yes | Yes | Yes | -5.34 | 0 | 0 | 2 | 3.58 |
| **SU[4,5]** | 58.23 | 4.13 | 5.06 | 5.7 | 3.66 | 4.47 | 4.6 | -5.92 | 1.20E-06 | -6.02 | 9.45E-07 | -7.72 | 1.92E-08 | High | No | No | No | Yes | Yes | Yes | Yes | -5.59 | 0 | 0 | 2 | 3.52 |

| **Supporting Table 2.** Results of the predictions obtained with the SwissADME web tool. The designed compounds are computed as protonated species. | | | | | | | | | | | | | | | | | | | | | | | | | | |
| --- | --- | --- | --- | --- | --- | --- | --- | --- | --- | --- | --- | --- | --- | --- | --- | --- | --- | --- | --- | --- | --- | --- | --- | --- | --- | --- |
| **Name** | **TPSA** | **iLOGP** | **XLOGP3** | **WLOGP** | **MLOGP** | **Silicos-IT Log P** | **Consensus Log P** | **ESOL Log S** | **ESOL Solubility (mol/l)** | **Ali**  **Log S** | **Ali Solubility (mol/l)** | **Silicos-IT LogSw** | **Silicos-IT Solubility (mol/l)** | **GI abs.** | **BBB permeant** | **Pgp substrate** | **CYP1A2 inhibitor** | **CYP2C19 inhibitor** | **CYP2C9 inhibitor** | **CYP2D6 inhibitor** | **CYP3A4 inhibitor** | **log Kp (cm/s)** | **Lipinski #violations** | **PAINS #alerts** | **Leadlikeness #violations** | **Synthetic Accessibility** |
| **RC-106** | 4.44 | 4.42 | 6.98 | 4.4 | 1.72 | 6.27 | 4.76 | -6.56 | 2.78E-07 | -6.89 | 1.30E-07 | -8.33 | 4.71E-09 | High | Yes | Yes | Yes | Yes | No | No | No | -3.52 | 0 | 0 | 2 | 3.35 |
| **RC-206** | 7.68 | 4.32 | 5.58 | 2.73 | 0.59 | 5.14 | 3.67 | -5.68 | 2.09E-06 | -5.5 | 3.14E-06 | -7.61 | 2.43E-08 | High | Yes | Yes | No | No | No | No | No | -4.52 | 0 | 0 | 2 | 2.95 |
| **RA[1,1]** | 7.68 | 4.2 | 4.66 | 1.72 | -0.05 | 3.96 | 2.9 | -4.5 | 3.13E-05 | -4.55 | 2.83E-05 | -4.61 | 2.44E-05 | High | Yes | No | No | No | No | Yes | No | -4.82 | 0 | 0 | 1 | 2.81 |
| **RA[1,2]** | 7.68 | 4.36 | 5.21 | 2.11 | 0.17 | 4.2 | 3.21 | -4.93 | 1.18E-05 | -5.12 | 7.61E-06 | -4.88 | 1.30E-05 | High | Yes | No | No | No | No | Yes | No | -4.51 | 0 | 0 | 1 | 2.95 |
| **RA[1,3]** | 7.68 | 4.16 | 5.21 | 3.75 | 0.73 | 5.15 | 3.8 | -5.38 | 4.14E-06 | -5.12 | 7.61E-06 | -6.81 | 1.54E-07 | High | Yes | No | No | No | No | No | No | -4.89 | 0 | 0 | 2 | 2.95 |
| **RA[1,4]** | 7.68 | 4.19 | 4.69 | 1.89 | 0.14 | 4.62 | 3.11 | -4.83 | 1.49E-05 | -4.58 | 2.63E-05 | -6.35 | 4.49E-07 | High | Yes | No | No | No | No | No | No | -4.93 | 0 | 0 | 1 | 2.82 |
| **RA[1,5]** | 7.68 | 4.03 | 4.53 | 2.7 | 0.68 | 4.92 | 3.37 | -4.85 | 1.42E-05 | -4.41 | 3.86E-05 | -6.51 | 3.11E-07 | High | Yes | No | No | No | No | Yes | No | -5.18 | 0 | 0 | 1 | 2.89 |
| **RA[2,1]** | 16.91 | 4.47 | 4.64 | 1.73 | -0.44 | 4 | 2.88 | -4.6 | 2.54E-05 | -4.72 | 1.90E-05 | -4.72 | 1.88E-05 | High | Yes | No | No | No | No | Yes | No | -5.02 | 0 | 0 | 1 | 2.91 |
| **RA[2,2]** | 16.91 | 4.63 | 5.18 | 2.12 | -0.22 | 4.25 | 3.19 | -5.01 | 9.66E-06 | -5.28 | 5.23E-06 | -5 | 1.01E-05 | High | Yes | No | No | No | No | Yes | No | -4.72 | 0 | 0 | 1 | 3.04 |
| **RA[2,3]** | 16.91 | 4.55 | 5.19 | 3.76 | 0.33 | 5.22 | 3.81 | -5.47 | 3.41E-06 | -5.29 | 5.11E-06 | -6.92 | 1.22E-07 | High | Yes | No | No | No | No | No | No | -5.09 | 0 | 0 | 2 | 3.03 |
| **RA[2,4]** | 16.91 | 4.35 | 4.67 | 1.89 | -0.25 | 4.67 | 3.07 | -4.91 | 1.24E-05 | -4.75 | 1.77E-05 | -6.46 | 3.48E-07 | High | Yes | No | No | No | No | No | No | -5.13 | 0 | 0 | 2 | 2.92 |
| **RA[2,5]** | 16.91 | 4.26 | 4.5 | 2.7 | 0.28 | 4.98 | 3.35 | -4.92 | 1.19E-05 | -4.58 | 2.65E-05 | -6.61 | 2.44E-07 | High | Yes | No | No | No | No | No | No | -5.38 | 0 | 0 | 2 | 2.96 |
| **RA[3,1]** | 7.68 | 4.62 | 5.91 | 2.87 | 0.61 | 5 | 3.8 | -5.69 | 2.07E-06 | -5.85 | 1.43E-06 | -6.26 | 5.50E-07 | High | Yes | Yes | No | Yes | No | No | No | -4.24 | 0 | 0 | 1 | 3.06 |
| **RA[3,2]** | 7.68 | 4.73 | 6.46 | 3.26 | 0.82 | 5.24 | 4.1 | -6.11 | 7.80E-07 | -6.42 | 3.84E-07 | -6.53 | 2.96E-07 | High | Yes | Yes | No | Yes | No | No | No | -3.93 | 0 | 0 | 2 | 3.2 |
| **RA[3,3]** | 7.68 | 4.57 | 6.46 | 4.9 | 1.36 | 6.2 | 4.7 | -6.53 | 2.92E-07 | -6.42 | 3.84E-07 | -8.44 | 3.60E-09 | High | Yes | Yes | No | No | No | No | No | -4.31 | 0 | 0 | 2 | 3.19 |
| **RA[3,4]** | 7.68 | 4.56 | 5.94 | 3.04 | 0.79 | 5.66 | 4 | -5.98 | 1.05E-06 | -5.88 | 1.33E-06 | -7.99 | 1.02E-08 | High | Yes | Yes | No | No | No | No | No | -4.35 | 0 | 0 | 2 | 3.07 |
| **RA[3,5]** | 7.68 | 4.53 | 5.78 | 3.85 | 1.33 | 5.97 | 4.29 | -6 | 1.00E-06 | -5.71 | 1.95E-06 | -8.14 | 7.16E-09 | High | Yes | Yes | No | No | No | No | No | -4.6 | 0 | 0 | 2 | 3.13 |
| **RA[4,1]** | 16.91 | 4.88 | 5.89 | 2.88 | 0.21 | 5.05 | 3.78 | -5.77 | 1.69E-06 | -6.02 | 9.59E-07 | -6.37 | 4.31E-07 | High | Yes | Yes | No | No | No | No | No | -4.43 | 0 | 0 | 2 | 3.13 |
| **RA[4,2]** | 16.91 | 5.04 | 6.43 | 3.27 | 0.41 | 5.3 | 4.09 | -6.19 | 6.45E-07 | -6.58 | 2.64E-07 | -6.63 | 2.33E-07 | High | Yes | Yes | No | No | No | No | No | -4.14 | 0 | 0 | 2 | 3.27 |
| **RA[4,3]** | 16.91 | 4.86 | 6.44 | 4.91 | 0.95 | 6.27 | 4.68 | -6.62 | 2.41E-07 | -6.59 | 2.58E-07 | -8.54 | 2.86E-09 | High | Yes | Yes | No | No | No | No | No | -4.51 | 0 | 0 | 2 | 3.25 |
| **RA[4,4]** | 16.91 | 4.91 | 5.92 | 3.05 | 0.38 | 5.72 | 4 | -6.06 | 8.75E-07 | -6.05 | 8.92E-07 | -8.09 | 8.05E-09 | High | Yes | Yes | No | No | No | No | No | -4.55 | 0 | 0 | 2 | 3.13 |
| **RA[4,5]** | 16.91 | 4.77 | 5.75 | 3.86 | 0.92 | 6.03 | 4.27 | -6.07 | 8.43E-07 | -5.87 | 1.34E-06 | -8.25 | 5.68E-09 | High | Yes | Yes | No | No | No | No | No | -4.8 | 0 | 0 | 2 | 3.18 |
| **AM[1,1]** | 24.75 | 3.68 | 3.84 | 1.25 | -0.61 | 3.49 | 2.33 | -4.07 | 8.60E-05 | -4.06 | 8.80E-05 | -4.15 | 7.08E-05 | High | Yes | No | No | Yes | No | No | No | -5.49 | 0 | 0 | 1 | 2.72 |
| **AM[1,2]** | 24.75 | 3.91 | 4.38 | 1.64 | -0.39 | 3.73 | 2.65 | -4.48 | 3.27E-05 | -4.62 | 2.42E-05 | -4.42 | 3.79E-05 | High | Yes | No | No | Yes | No | No | No | -5.19 | 0 | 0 | 1 | 2.85 |
| **AM[1,3]** | 24.75 | 3.89 | 4.84 | 3.54 | 0.45 | 4.69 | 3.48 | -5.22 | 5.96E-06 | -5.09 | 8.06E-06 | -6.34 | 4.52E-07 | High | Yes | No | No | No | No | No | No | -5.24 | 0 | 0 | 2 | 2.83 |
| **AM[1,4]** | 24.75 | 3.78 | 4.32 | 1.68 | -0.14 | 4.15 | 2.76 | -4.67 | 2.15E-05 | -4.55 | 2.79E-05 | -5.88 | 1.31E-06 | High | Yes | No | No | Yes | No | No | No | -5.28 | 0 | 0 | 1 | 2.72 |
| **AM[1,5]** | 24.75 | 3.59 | 4.16 | 2.49 | 0.4 | 4.46 | 3.02 | -4.69 | 2.05E-05 | -4.39 | 4.09E-05 | -6.04 | 9.10E-07 | High | Yes | No | No | No | No | No | No | -5.53 | 0 | 0 | 2 | 2.78 |
| **AM[2,1]** | 33.98 | 4.1 | 3.81 | 1.25 | -0.97 | 3.54 | 2.35 | -4.15 | 7.06E-05 | -4.22 | 6.05E-05 | -4.26 | 5.50E-05 | High | Yes | No | No | No | No | No | No | -5.69 | 0 | 0 | 1 | 2.83 |
| **AM[2,2]** | 33.98 | 4.16 | 4.35 | 1.64 | -0.75 | 3.79 | 2.64 | -4.57 | 2.68E-05 | -4.78 | 1.66E-05 | -4.53 | 2.95E-05 | High | Yes | No | No | No | No | Yes | No | -5.39 | 0 | 0 | 2 | 2.96 |
| **AM[2,3]** | 33.98 | 4.12 | 4.81 | 3.55 | 0.08 | 4.77 | 3.46 | -5.3 | 4.96E-06 | -5.26 | 5.54E-06 | -6.45 | 3.58E-07 | High | Yes | No | No | No | No | No | No | -5.44 | 0 | 0 | 2 | 2.93 |
| **AM[2,4]** | 33.98 | 4.04 | 4.29 | 1.69 | -0.51 | 4.21 | 2.74 | -4.74 | 1.81E-05 | -4.72 | 1.92E-05 | -5.99 | 1.02E-06 | High | Yes | No | No | No | No | No | No | -5.48 | 0 | 0 | 2 | 2.83 |
| **AM[2,5]** | 33.98 | 3.86 | 4.13 | 2.5 | 0.03 | 4.53 | 3.01 | -4.77 | 1.72E-05 | -4.55 | 2.82E-05 | -6.15 | 7.14E-07 | High | Yes | No | No | No | No | No | No | -5.73 | 0 | 0 | 2 | 2.87 |
| **AM[3,1]** | 24.75 | 4.1 | 5.09 | 2.4 | 0.06 | 4.54 | 3.24 | -5.24 | 5.69E-06 | -5.35 | 4.44E-06 | -5.79 | 1.61E-06 | High | Yes | Yes | No | Yes | No | No | No | -4.9 | 0 | 0 | 2 | 2.96 |
| **AM[3,2]** | 24.75 | 4.25 | 5.63 | 2.79 | 0.27 | 4.78 | 3.54 | -5.66 | 2.18E-06 | -5.91 | 1.22E-06 | -6.06 | 8.65E-07 | High | Yes | Yes | No | Yes | No | No | No | -4.61 | 0 | 0 | 2 | 3.1 |
| **AM[3,3]** | 24.75 | 4.19 | 6.09 | 4.69 | 1.08 | 5.74 | 4.36 | -6.38 | 4.21E-07 | -6.39 | 4.07E-07 | -7.97 | 1.06E-08 | High | Yes | Yes | No | Yes | No | No | No | -4.66 | 0 | 0 | 2 | 3.07 |
| **AM[3,4]** | 24.75 | 4.23 | 5.57 | 2.83 | 0.51 | 5.2 | 3.67 | -5.82 | 1.52E-06 | -5.85 | 1.41E-06 | -7.52 | 2.99E-08 | High | Yes | Yes | No | Yes | No | No | No | -4.7 | 0 | 0 | 2 | 2.96 |
| **AM[3,5]** | 24.75 | 4.06 | 5.4 | 3.64 | 1.05 | 5.51 | 3.93 | -5.83 | 1.47E-06 | -5.67 | 2.12E-06 | -7.68 | 2.10E-08 | High | Yes | Yes | No | No | No | No | No | -4.95 | 0 | 0 | 2 | 3.02 |
| **AM[4,1]** | 33.98 | 4.36 | 5.06 | 2.41 | -0.32 | 4.59 | 3.22 | -5.33 | 4.71E-06 | -5.52 | 3.05E-06 | -5.9 | 1.26E-06 | High | Yes | Yes | No | No | No | No | No | -5.11 | 0 | 0 | 2 | 3.05 |
| **AM[4,2]** | 33.98 | 4.61 | 5.6 | 2.8 | -0.11 | 4.83 | 3.55 | -5.75 | 1.80E-06 | -6.08 | 8.40E-07 | -6.17 | 6.82E-07 | High | Yes | Yes | No | No | No | No | No | -4.81 | 0 | 0 | 2 | 3.19 |
| **AM[4,3]** | 33.98 | 4.62 | 6.06 | 4.7 | 0.7 | 5.82 | 4.38 | -6.46 | 3.51E-07 | -6.55 | 2.80E-07 | -8.07 | 8.47E-09 | High | Yes | Yes | No | Yes | No | No | No | -4.86 | 0 | 0 | 2 | 3.15 |
| **AM[4,4]** | 33.98 | 4.54 | 5.54 | 2.84 | 0.13 | 5.26 | 3.66 | -5.89 | 1.28E-06 | -6.01 | 9.69E-07 | -7.63 | 2.37E-08 | High | Yes | Yes | No | Yes | No | No | No | -4.9 | 0 | 0 | 2 | 3.05 |
| **AM[4,5]** | 33.98 | 4.31 | 5.38 | 3.65 | 0.67 | 5.58 | 3.92 | -5.92 | 1.21E-06 | -5.85 | 1.42E-06 | -7.78 | 1.67E-08 | High | Yes | Yes | No | No | No | No | No | -5.15 | 0 | 0 | 2 | 3.09 |
| **SU[1,1]** | 50.2 | 3.41 | 3.35 | 1.88 | -1.36 | 2.38 | 1.93 | -3.97 | 1.07E-04 | -4.08 | 8.29E-05 | -4.11 | 7.85E-05 | High | Yes | No | No | No | No | No | No | -6.05 | 0 | 0 | 0 | 3.48 |
| **SU[1,2]** | 50.2 | 3.66 | 3.89 | 2.27 | -1.14 | 2.62 | 2.26 | -4.39 | 4.05E-05 | -4.64 | 2.28E-05 | -4.37 | 4.23E-05 | High | Yes | No | No | No | No | No | No | -5.76 | 0 | 0 | 2 | 3.6 |
| **SU[1,3]** | 50.2 | 3.85 | 4.53 | 4.17 | -0.28 | 3.6 | 3.17 | -5.24 | 5.73E-06 | -5.31 | 4.94E-06 | -6.29 | 5.14E-07 | High | Yes | No | No | Yes | Yes | No | No | -5.68 | 0 | 0 | 2 | 3.35 |
| **SU[1,4]** | 50.2 | 3.48 | 4.01 | 2.31 | -0.88 | 3.04 | 2.39 | -4.68 | 2.08E-05 | -4.77 | 1.71E-05 | -5.84 | 1.46E-06 | High | Yes | No | No | No | Yes | No | No | -5.72 | 0 | 0 | 2 | 3.35 |
| **SU[1,5]** | 50.2 | 3.45 | 3.84 | 3.12 | -0.34 | 3.37 | 2.69 | -4.7 | 2.01E-05 | -4.59 | 2.57E-05 | -5.99 | 1.02E-06 | High | Yes | No | No | No | Yes | No | No | -5.97 | 0 | 0 | 2 | 3.28 |
| **SU[2,1]** | 59.43 | 3.84 | 3.32 | 1.89 | -1.7 | 2.44 | 1.96 | -4.06 | 8.72E-05 | -4.24 | 5.70E-05 | -4.21 | 6.15E-05 | High | Yes | Yes | No | No | No | No | No | -6.26 | 0 | 0 | 1 | 3.5 |
| **SU[2,2]** | 59.43 | 3.95 | 3.87 | 2.28 | -1.47 | 2.68 | 2.26 | -4.49 | 3.26E-05 | -4.82 | 1.53E-05 | -4.48 | 3.32E-05 | High | Yes | No | No | No | No | No | No | -5.95 | 0 | 0 | 2 | 3.62 |
| **SU[2,3]** | 59.43 | 4.06 | 4.5 | 4.18 | -0.63 | 3.67 | 3.16 | -5.32 | 4.75E-06 | -5.47 | 3.40E-06 | -6.39 | 4.09E-07 | High | Yes | No | No | Yes | Yes | No | No | -5.88 | 0 | 0 | 2 | 3.4 |
| **SU[2,4]** | 59.43 | 4.06 | 3.98 | 2.32 | -1.22 | 3.11 | 2.45 | -4.76 | 1.75E-05 | -4.93 | 1.18E-05 | -5.94 | 1.15E-06 | High | Yes | No | No | No | Yes | No | No | -5.92 | 0 | 0 | 2 | 3.39 |
| **SU[2,5]** | 59.43 | 3.74 | 3.81 | 3.13 | -0.68 | 3.43 | 2.69 | -4.78 | 1.67E-05 | -4.75 | 1.77E-05 | -6.09 | 8.11E-07 | High | Yes | No | No | No | Yes | No | No | -6.18 | 0 | 0 | 2 | 3.33 |
| **SU[3,1]** | 50.2 | 3.93 | 4.6 | 3.04 | -0.67 | 3.42 | 2.86 | -5.15 | 7.09E-06 | -5.38 | 4.18E-06 | -5.74 | 1.81E-06 | High | Yes | Yes | No | No | Yes | No | No | -5.47 | 0 | 0 | 2 | 3.72 |
| **SU[3,2]** | 50.2 | 4.06 | 5.14 | 3.43 | -0.46 | 3.66 | 3.17 | -5.57 | 2.71E-06 | -5.94 | 1.15E-06 | -6.01 | 9.79E-07 | High | Yes | Yes | No | No | Yes | No | No | -5.17 | 0 | 0 | 2 | 3.84 |
| **SU[3,3]** | 50.2 | 4.17 | 5.78 | 5.32 | 0.37 | 4.64 | 4.06 | -6.39 | 4.05E-07 | -6.6 | 2.49E-07 | -7.92 | 1.22E-08 | High | Yes | No | No | Yes | Yes | No | No | -5.1 | 0 | 0 | 2 | 3.59 |
| **SU[3,4]** | 50.2 | 4.26 | 5.26 | 3.46 | -0.21 | 4.09 | 3.37 | -5.83 | 1.47E-06 | -6.06 | 8.64E-07 | -7.47 | 3.40E-08 | High | Yes | No | No | No | Yes | No | No | -5.14 | 0 | 0 | 2 | 3.58 |
| **SU[3,5]** | 50.2 | 3.82 | 5.09 | 4.27 | 0.33 | 4.4 | 3.58 | -5.85 | 1.42E-06 | -5.89 | 1.30E-06 | -7.62 | 2.40E-08 | High | Yes | No | No | No | Yes | No | No | -5.39 | 0 | 0 | 2 | 3.51 |
| **SU[4,1]** | 59.43 | 4.21 | 4.57 | 3.04 | -1.02 | 3.49 | 2.86 | -5.23 | 5.84E-06 | -5.54 | 2.87E-06 | -5.84 | 1.44E-06 | High | Yes | Yes | No | No | Yes | No | No | -5.68 | 0 | 0 | 2 | 3.74 |
| **SU[4,2]** | 59.43 | 4.38 | 5.12 | 3.43 | -0.82 | 3.73 | 3.17 | -5.66 | 2.19E-06 | -6.11 | 7.72E-07 | -6.11 | 7.80E-07 | High | Yes | Yes | No | No | Yes | No | No | -5.37 | 0 | 0 | 2 | 3.86 |
| **SU[4,3]** | 59.43 | 4.48 | 5.75 | 5.33 | 0 | 4.72 | 4.06 | -6.47 | 3.36E-07 | -6.77 | 1.71E-07 | -8.01 | 9.79E-09 | High | Yes | No | No | Yes | Yes | No | No | -5.3 | 1 | 0 | 2 | 3.63 |
| **SU[4,4]** | 59.43 | 4.47 | 5.23 | 3.47 | -0.56 | 4.16 | 3.35 | -5.91 | 1.23E-06 | -6.23 | 5.94E-07 | -7.57 | 2.70E-08 | High | Yes | No | No | No | Yes | No | No | -5.34 | 0 | 0 | 2 | 3.62 |
| **SU[4,5]** | 59.43 | 4.13 | 5.06 | 4.28 | -0.03 | 4.47 | 3.58 | -5.93 | 1.18E-06 | -6.05 | 8.91E-07 | -7.72 | 1.92E-08 | High | Yes | No | No | Yes | Yes | No | No | -5.6 | 0 | 0 | 2 | 3.56 |

**^1^H and ^13^C NMR of the intermediates**

**^1^H NMR (300 MHz, CDCl_3_) *t*-butyl-4-[(2*E*)-3-phenylbut-2-en-1-yl]piperazine-1-carboxylate (Gate 2081)**


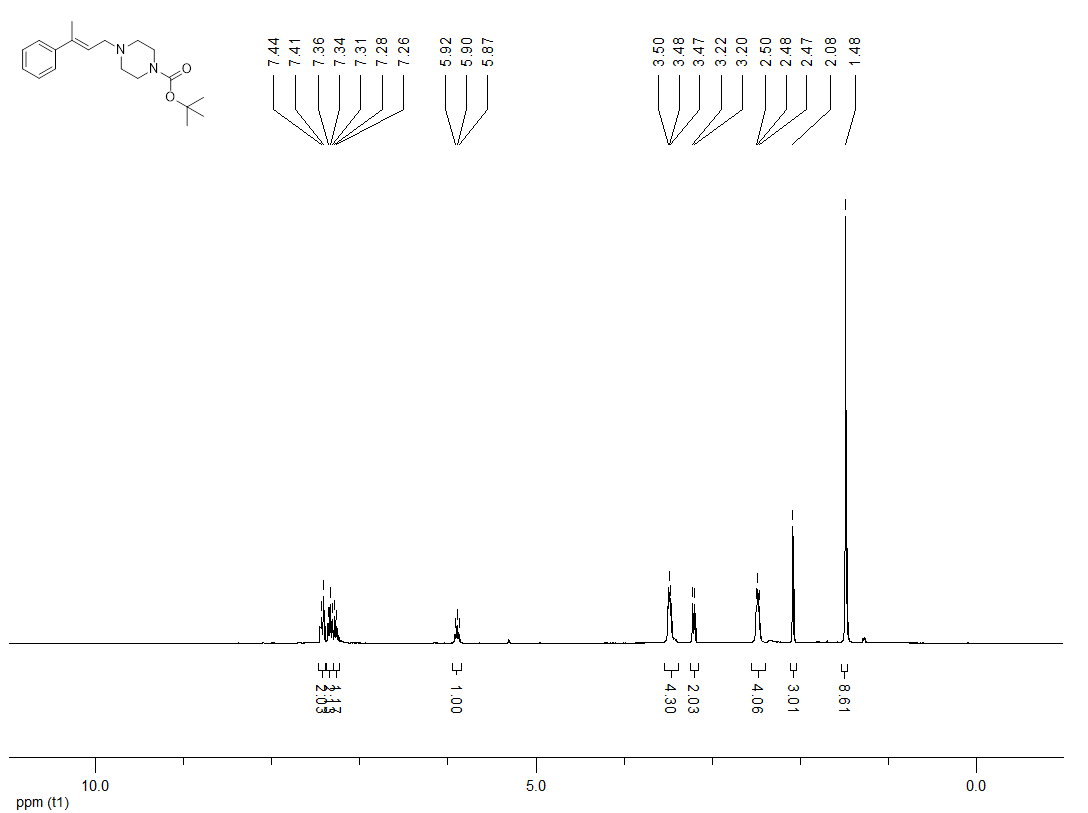


**^13^C NMR (100 MHz, CDCl_3_) *t*-butyl-4-[(2*E*)-3-phenylbut-2-en-1-yl]piperazine-1-carboxylate (Gate 2081)**


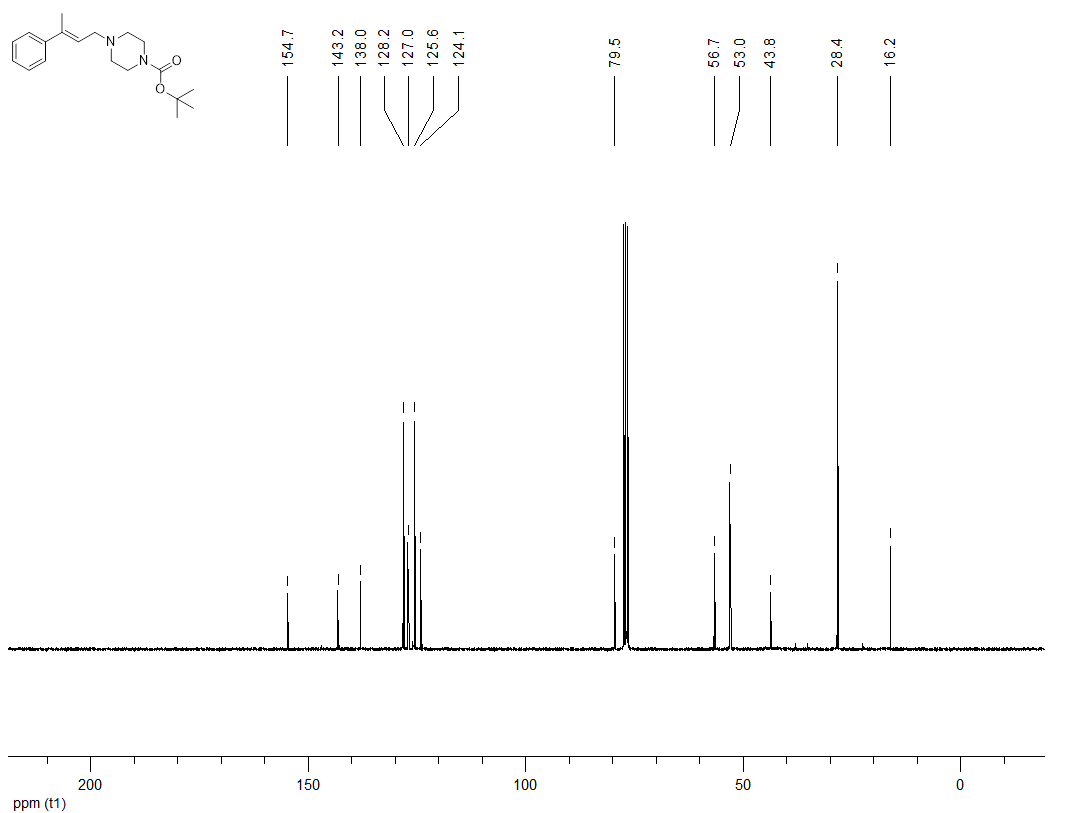


**^1^H NMR (300 MHz, CDCl_3_) *t*-butyl-4-[(2*E*)-3-(4-methoxyphenyl)but-2-en-1-yl]piperazine-1-carboxylate (Gate 2082)**


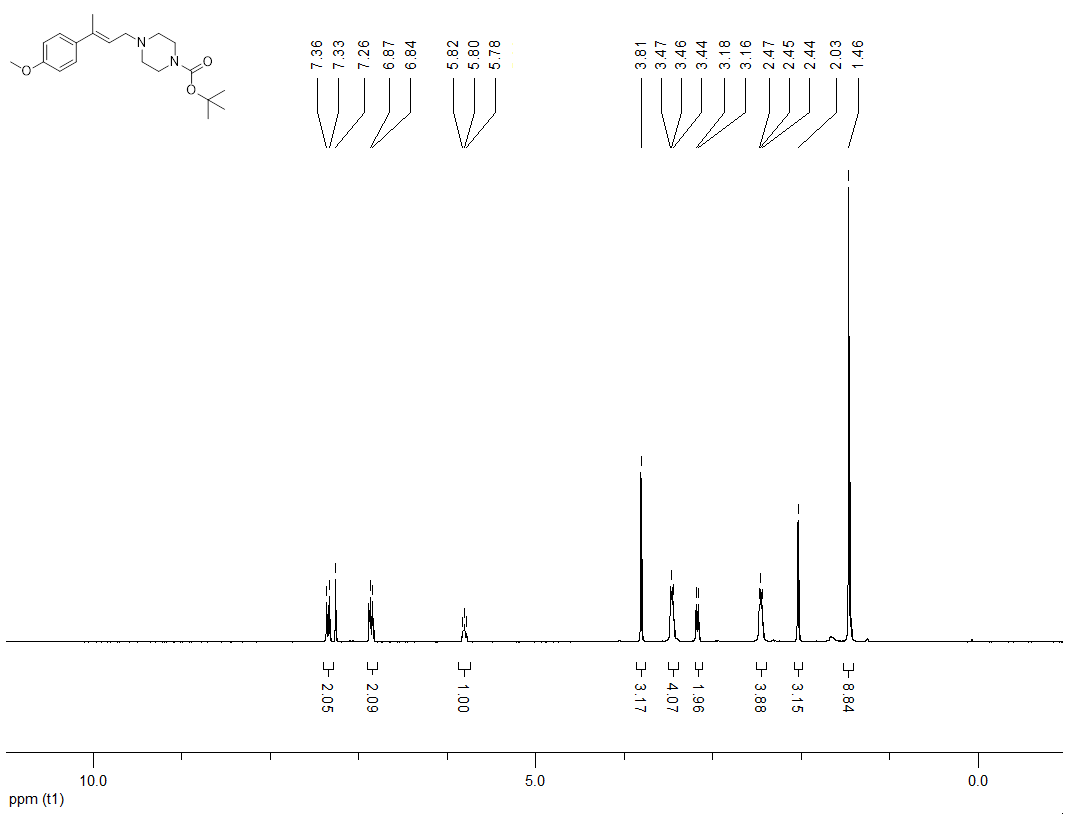


**^13^C NMR (100 MHz, CDCl_3_) *t*-butyl-4-[(2*E*)-3-(4-methoxyphenyl)but-2-en-1-yl]piperazine-1-carboxylate (Gate 2082)**


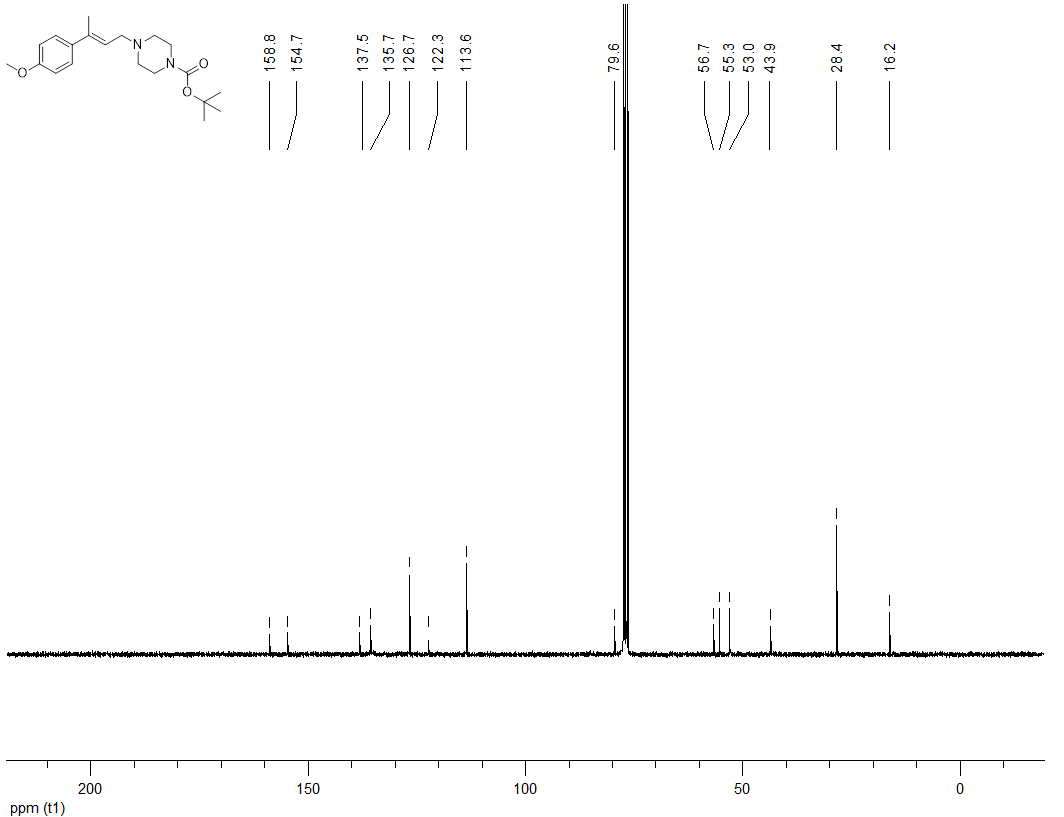


**^1^H NMR (300 MHz, CDCl_3_) *t*-butyl-4-[(2*E*)-3-(naphthalen-2-yl)but-2-en-1-yl]piperazine-1-carboxylate (Gate 2083)**

**
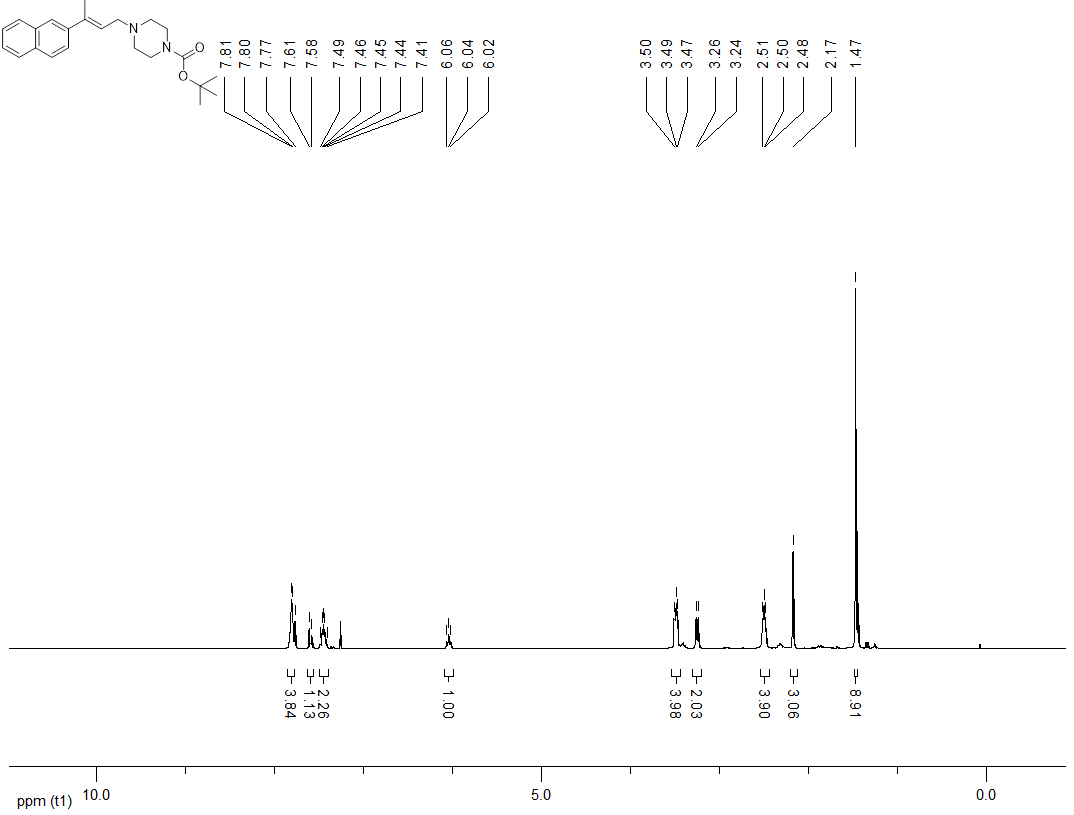
**

**^13^C NMR (100 MHz, CDCl_3_) *t*-butyl-4-[(2*E*)-3-(naphthalen-2-yl)but-2-en-1-yl]piperazine-1-carboxylate (Gate 2083)**

**
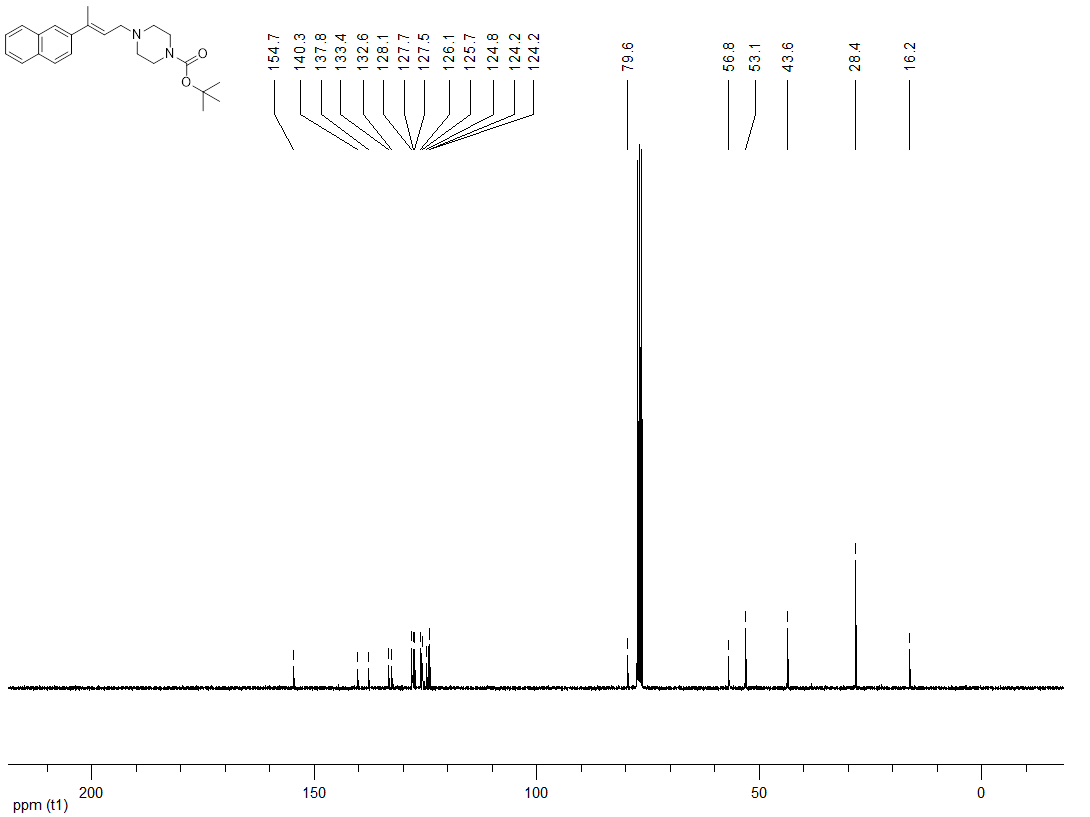
**

**^1^H NMR (300 MHz, CDCl_3_) *t*-butyl-4-[(2*E*)-3-(6-methoxynaphthalen-2-yl)but-2-en-1-yl]piperazine-1-carboxylate (Gate 2084)**

**
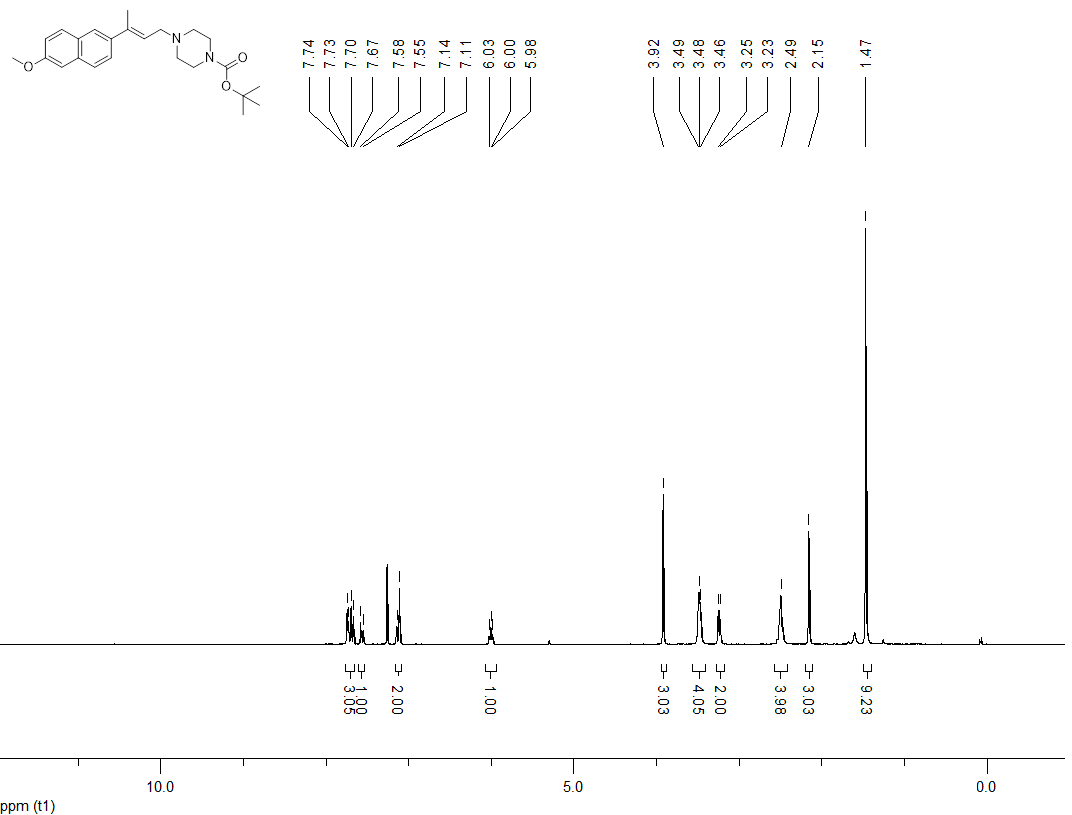
**

**^13^C NMR (100 MHz, CDCl_3_) *t*-butyl-4-[(2*E*)-3-(6-methoxynaphthalen-2-yl)but-2-en-1-yl]piperazine-1-carboxylate (Gate 2084)**

**
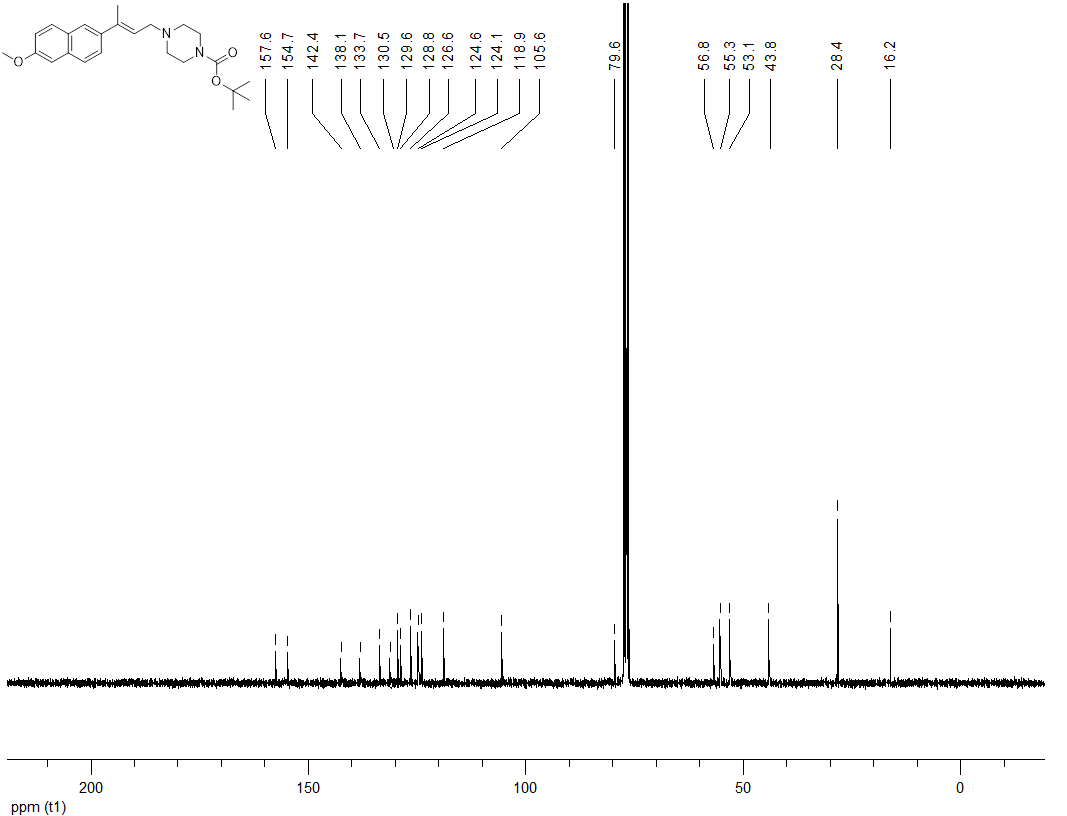
**

**^1^H NMR (300 MHz, CDCl_3_) 1-[(2*E*)-3-phenylbut-2-en-1-yl]piperazine (Gate 2085)**

**
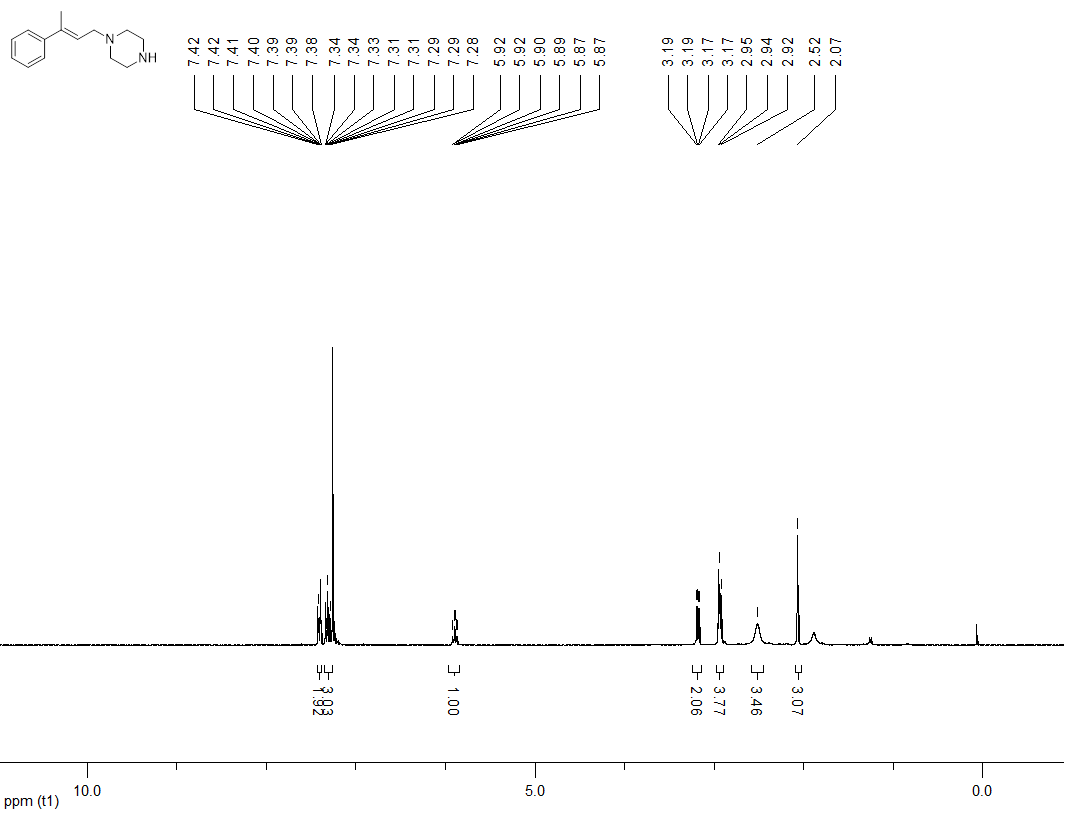
**

**^13^C NMR (100 MHz, CDCl_3_) 1-[(2*E*)-3-phenylbut-2-en-1-yl]piperazine (Gate 2085)**

**
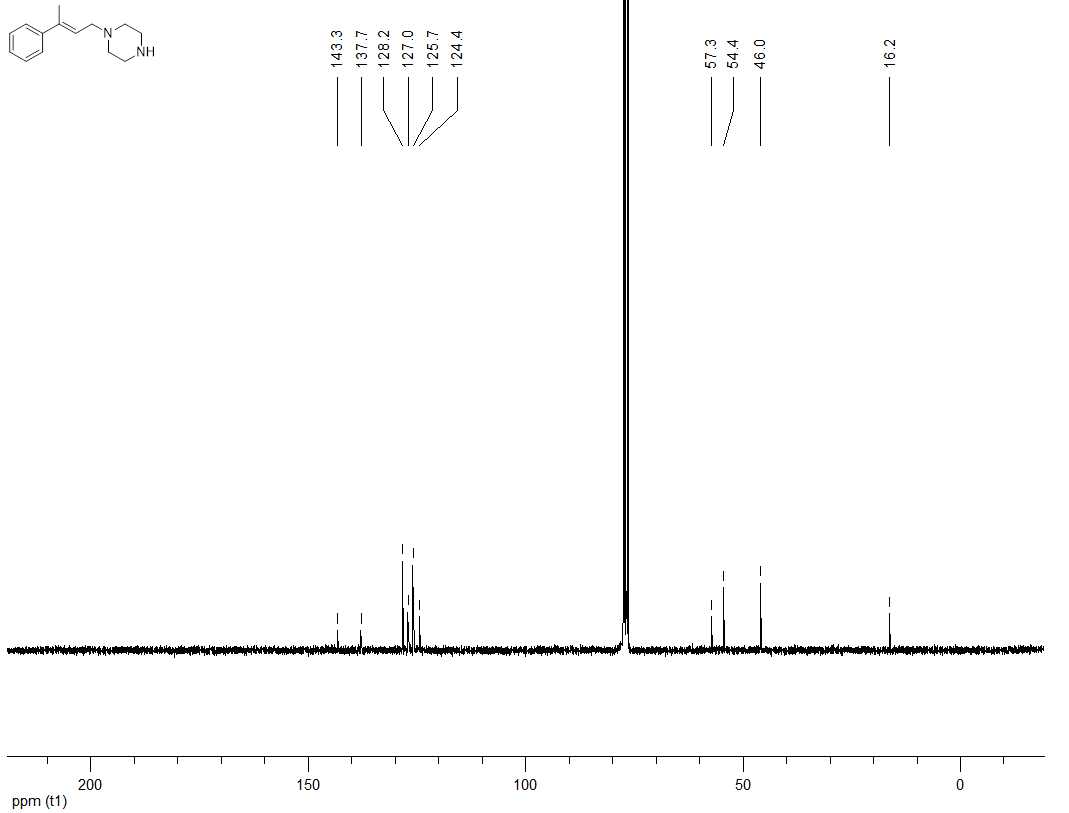
**

**^1^H NMR (300 MHz, CDCl_3_) 1-[(2*E*)-3-(4-methoxyphenyl)but-2-en-1-yl]piperazine (Gate 2086)**


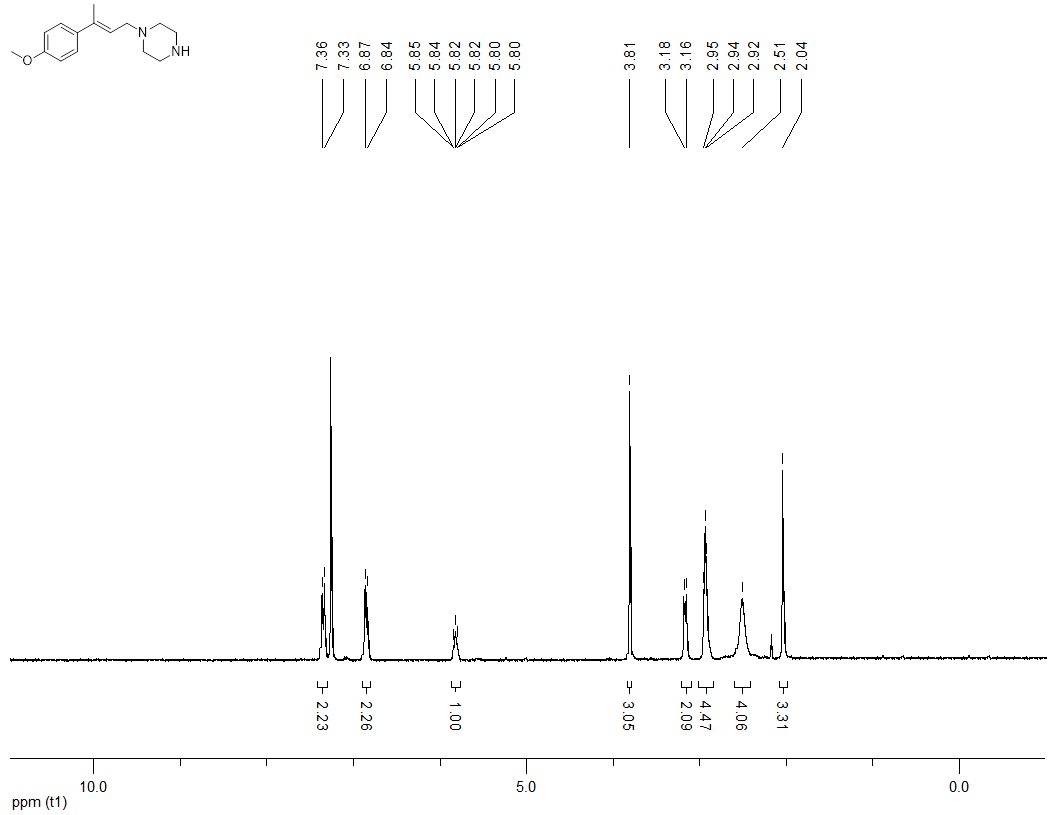


**^13^C NMR (100 MHz, CDCl_3_) 1-[(2*E*)-3-(4-methoxyphenyl)but-2-en-1-yl]piperazine (Gate 2086)**

**
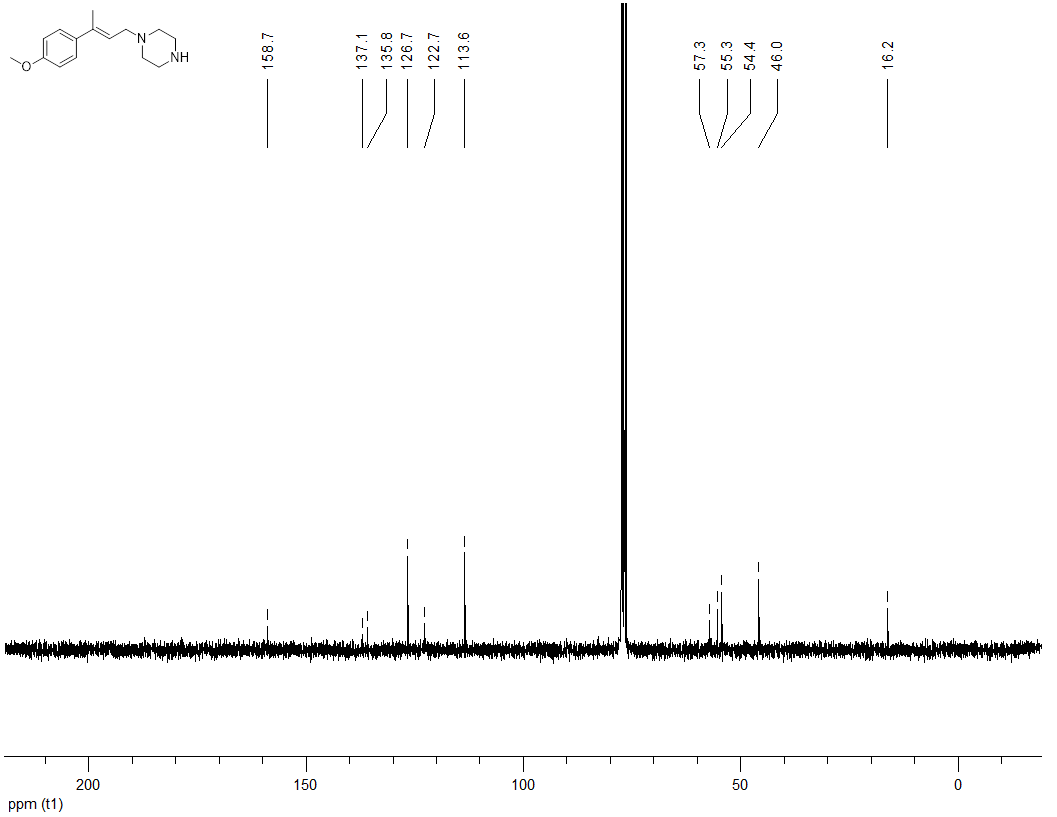
**

**^1^H NMR (300 MHz, CDCl_3_) 1-[(2*E*)-3-(naphthalen-2-yl)but-2-en-1-yl]piperazine (Gate 2087)**


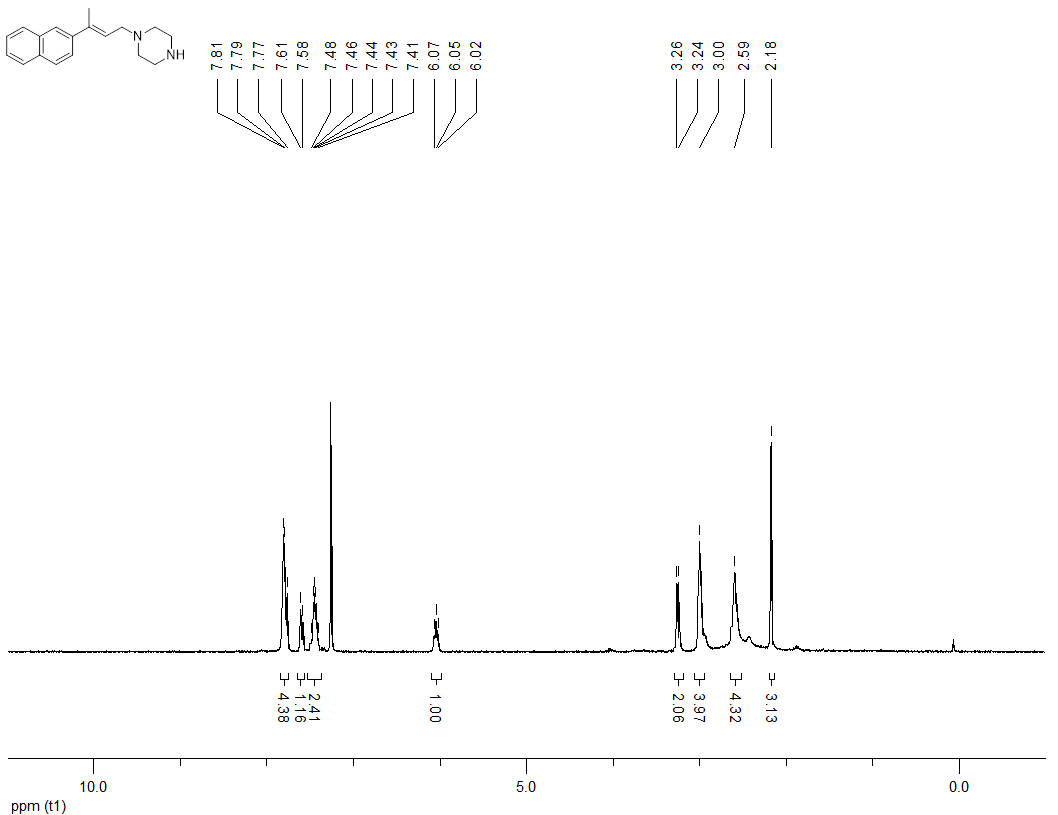


**^13^C NMR (100 MHz, CDCl_3_) 1-[(2*E*)-3-(naphthalen-2-yl)but-2-en-1-yl]piperazine (Gate 2087)**

**
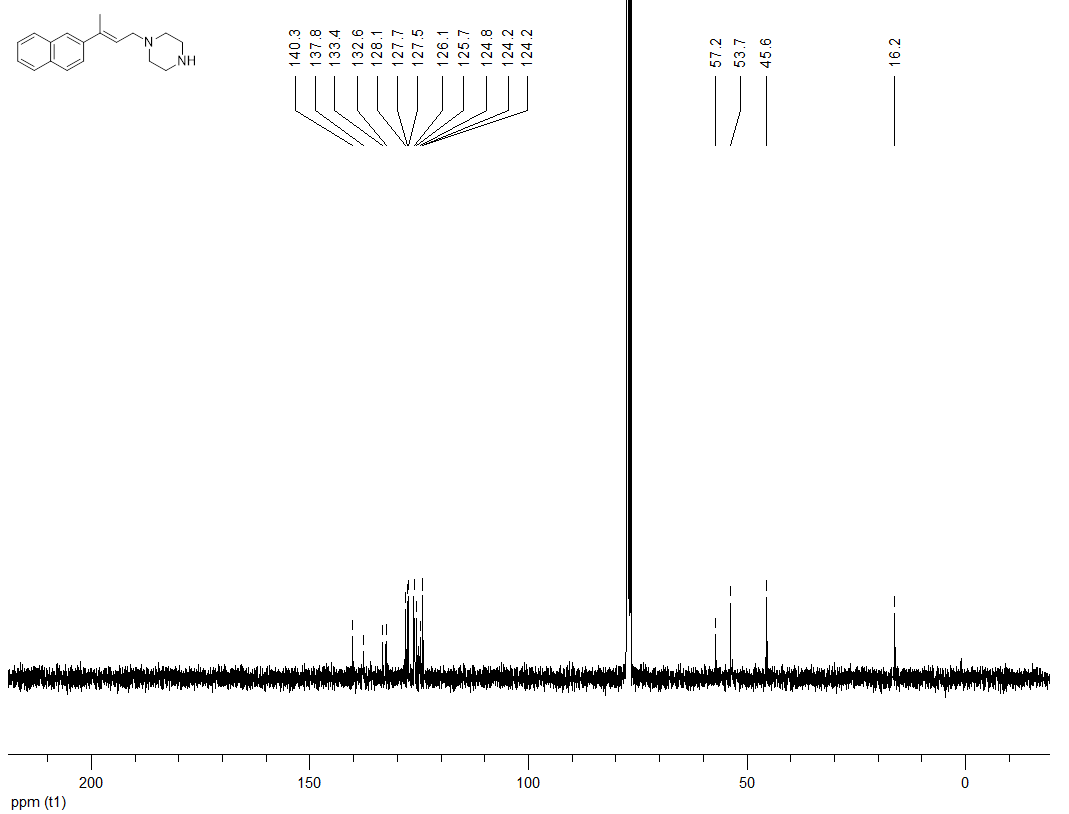
**

**^1^H NMR (300 MHz, CDCl_3_) 1-[(2*E*)-3-(6-methoxynaphthalen-2-yl)but-2-en-1-yl]piperazine (Gate 2088)**


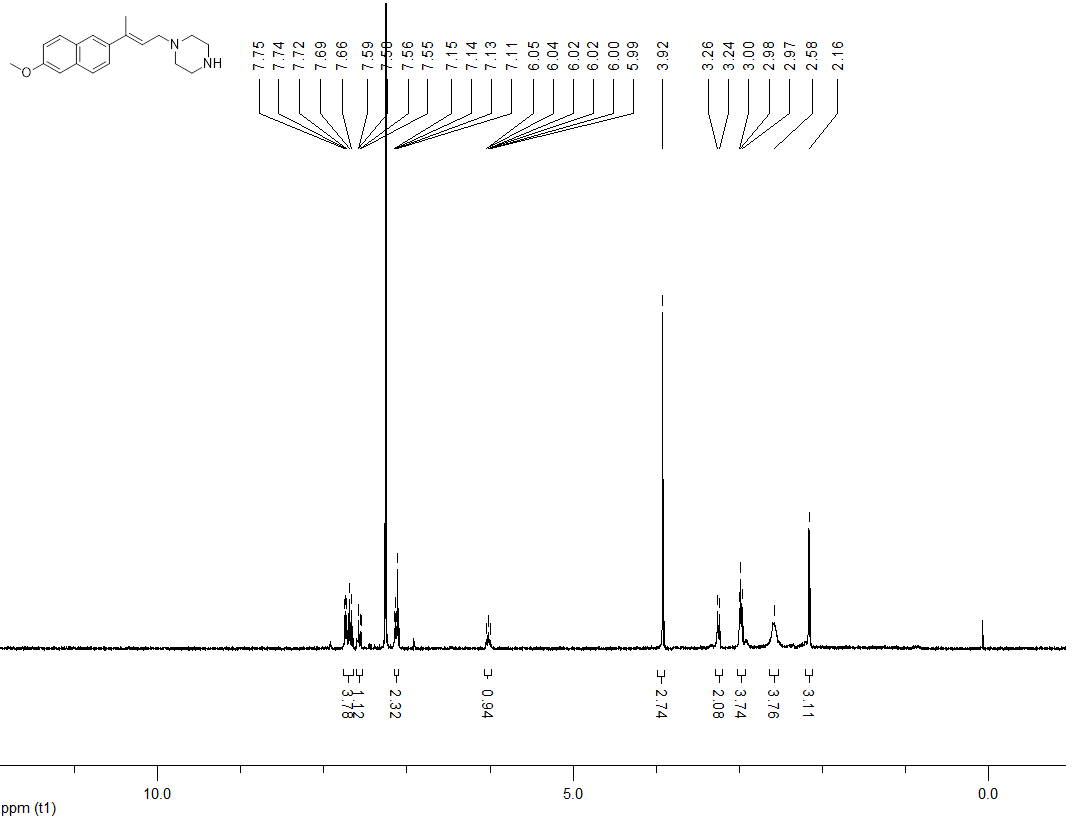


**^13^C NMR (100 MHz, CDCl_3_) 1-[(2*E*)-3-(6-methoxynaphthalen-2-yl)but-2-en-1-yl]piperazine (Gate 2088)**

**
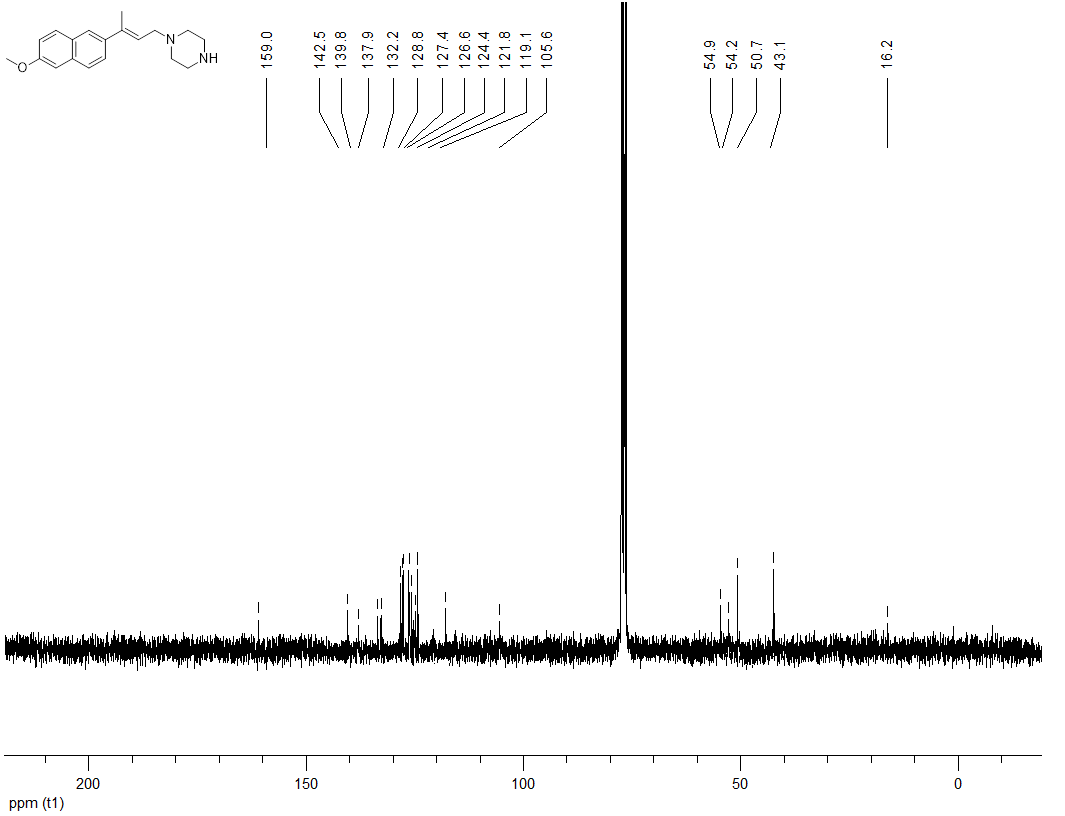
**

**^1^H and ^13^C NMR of some representative amidification products**

**^1^H NMR (300 MHz, CDCl_3_) 1-(2,6-difluorobenzoyl)-4-[(2*E*)-3-phenylbut-2-en-1-yl]piperazine (Gate 2144)**

**
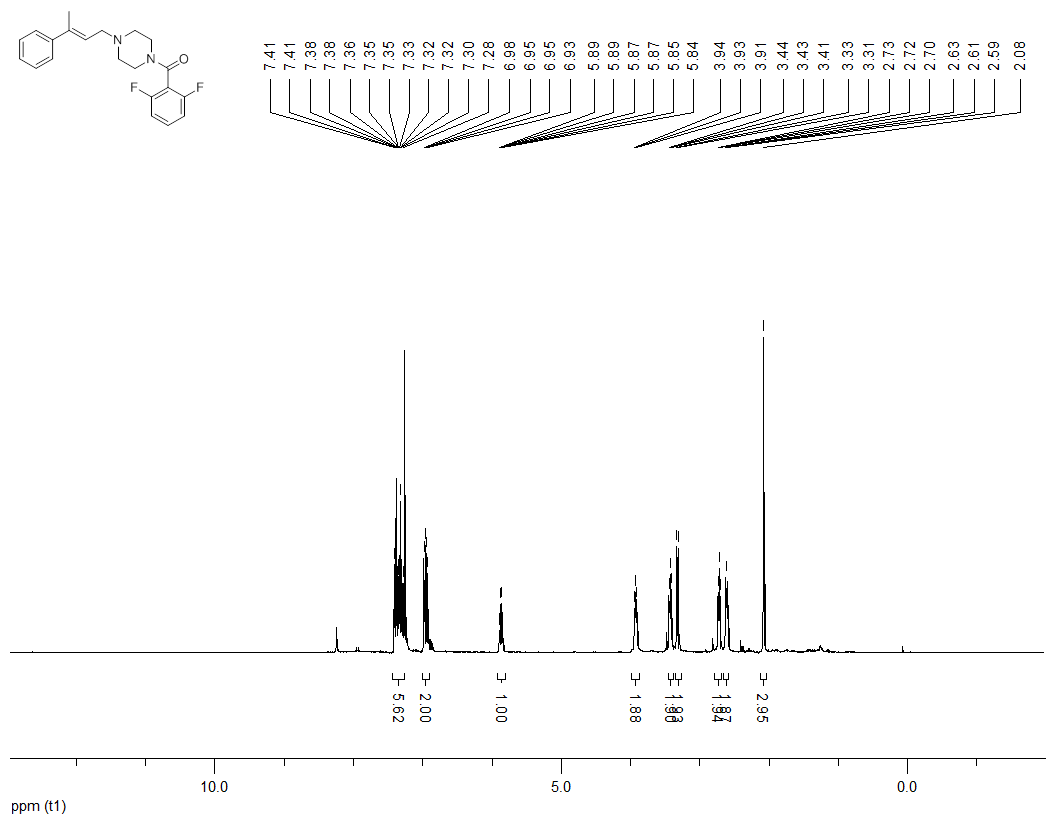
**

**^13^C NMR (100 MHz, CDCl_3_) 1-(2,6-difluorobenzoyl)-4-[(2*E*)-3-phenylbut-2-en-1-yl]piperazine (Gate 2144)**

**
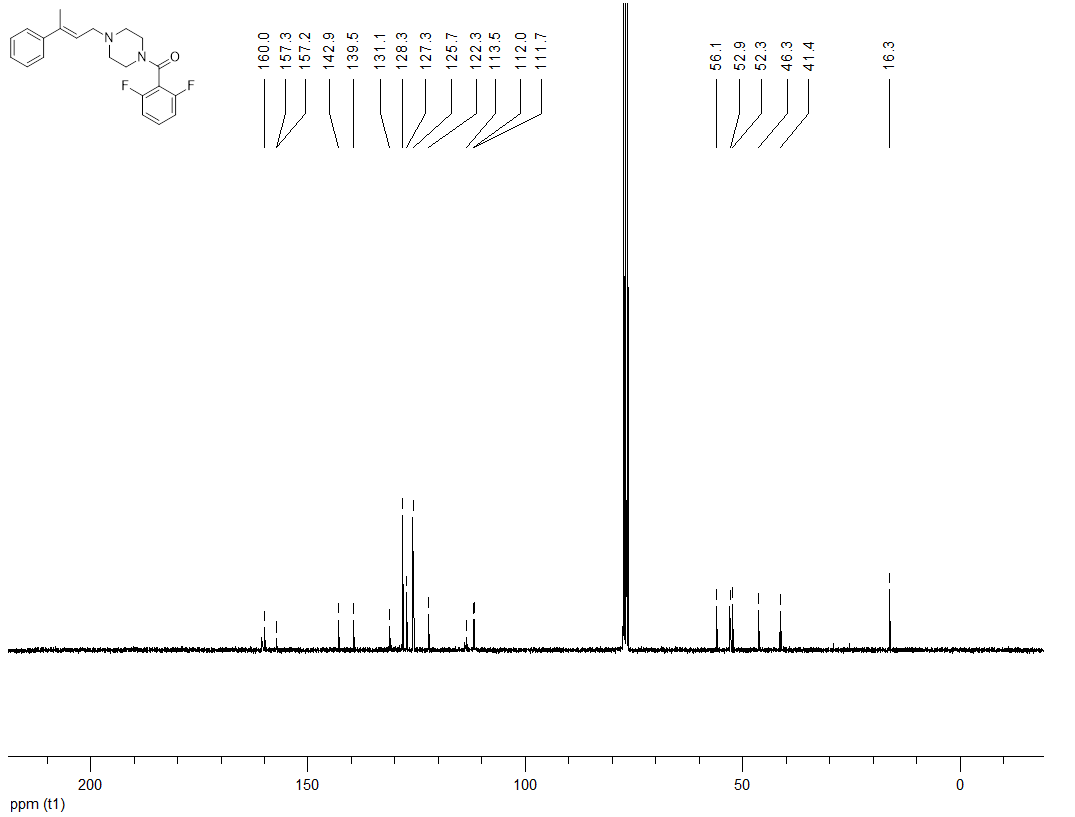
**

**^1^H NMR (300 MHz, CDCl_3_) 1-[(2*E*)-3-(4-methoxyphenyl)but-2-en-1-yl]-4-(4-methylbenzoyl)piperazine (Gate 2149)**

**
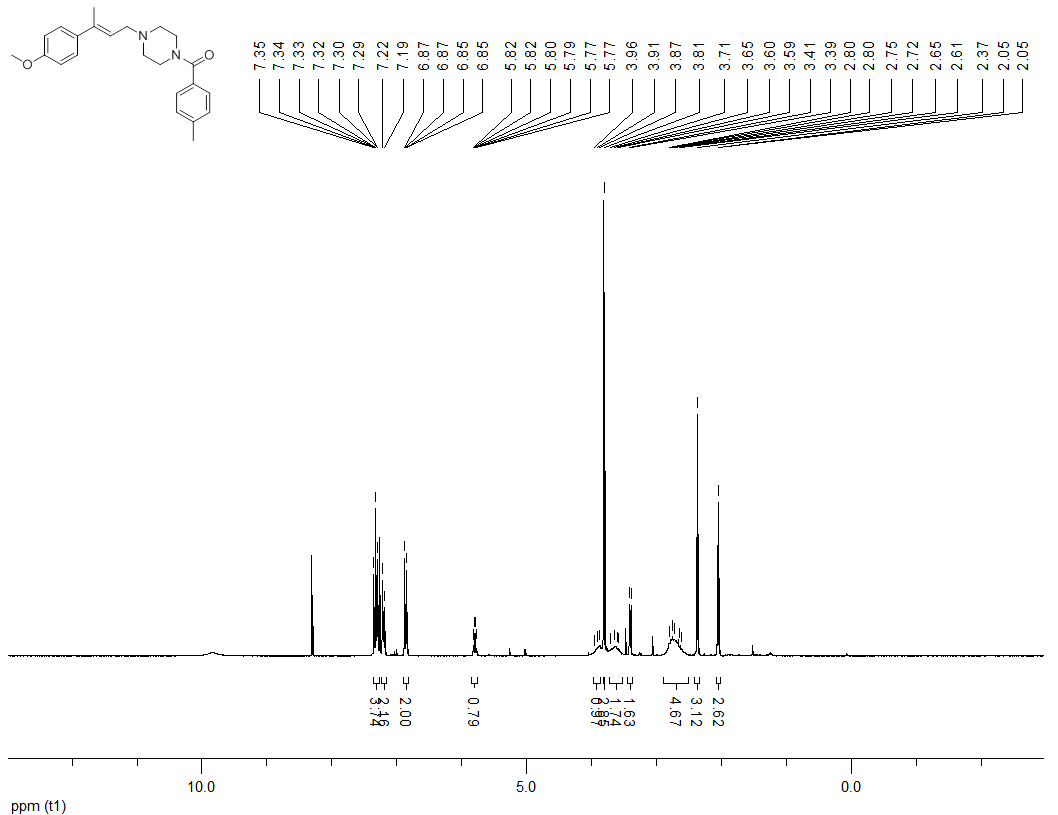
**

**^13^C NMR (100 MHz, CDCl_3_) 1-[(2*E*)-3-(4-methoxyphenyl)but-2-en-1-yl]-4-(4-methylbenzoyl)piperazine (Gate 2149)**

**
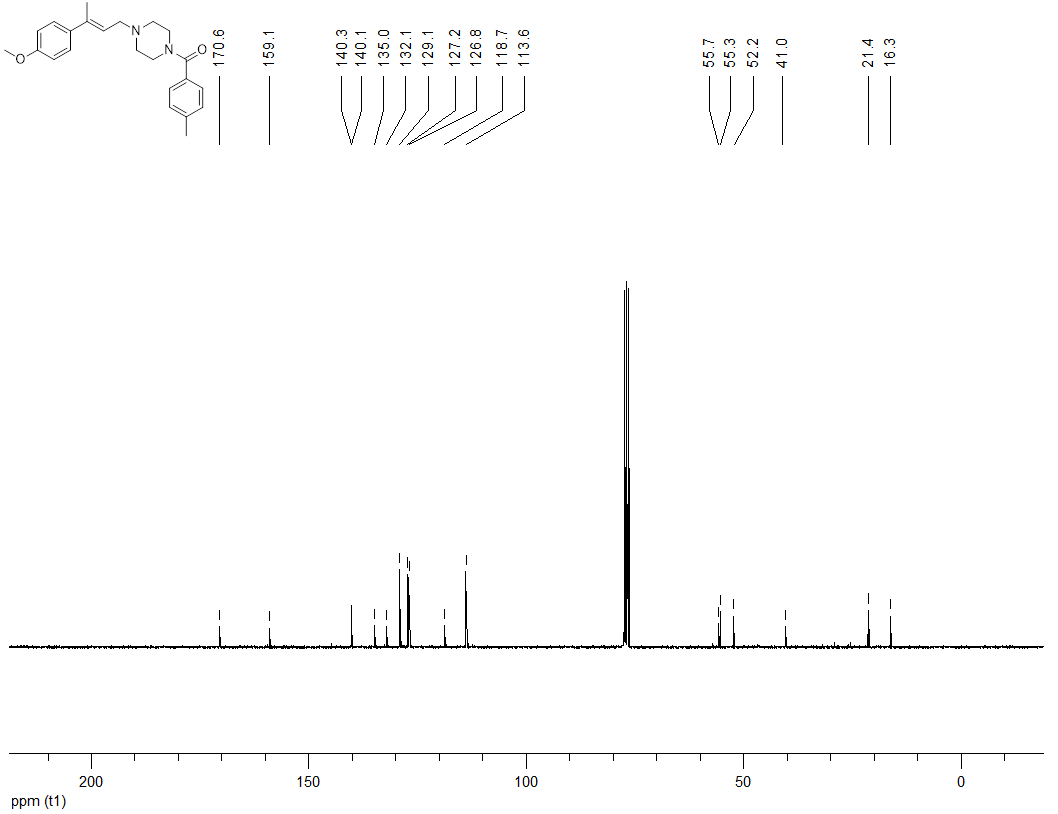
**

**^1^H NMR (300 MHz, CDCl_3_) 1-cyclopentanecarbonyl-4-[(2*E*)-3-(naphthalen-2-yl)but-2-en-1-yl]piperazine (Gate 2152)**

**
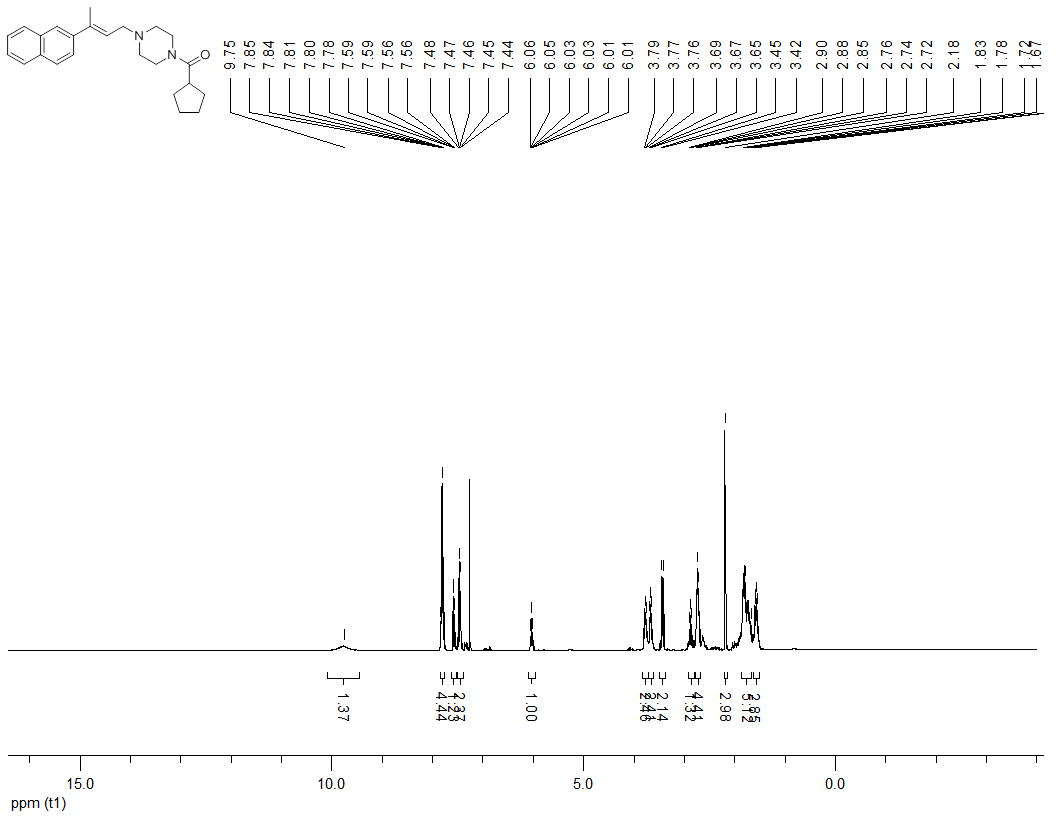
**

**^13^C NMR (100 MHz, CDCl_3_) 1-cyclopentanecarbonyl-4-[(2*E*)-3-(naphthalen-2-yl)but-2-en-1-yl]piperazine (Gate 2152)**

**
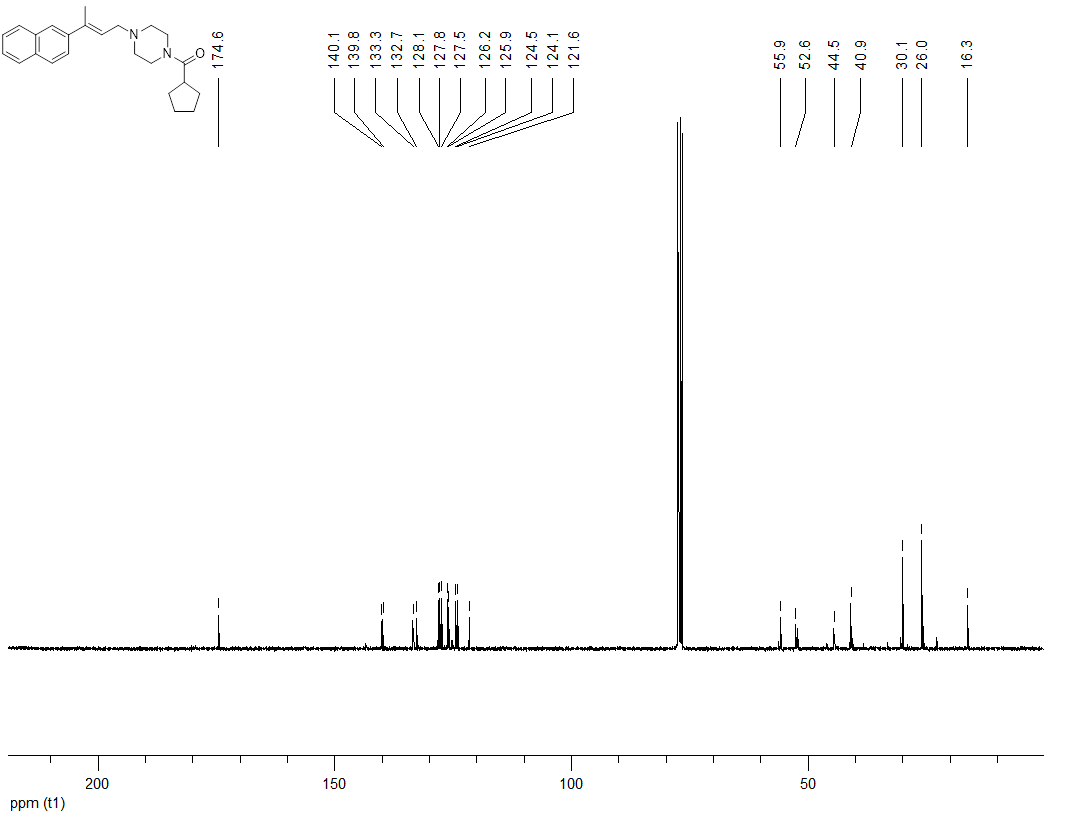
**

**^1^H NMR (300 MHz, CDCl_3_) 1-(2,6-difluorobenzoyl)-4-[(2*E*)-3-(naphthalen-2-yl)but-2-en-1-yl]piperazine (Gate 2156)**

**
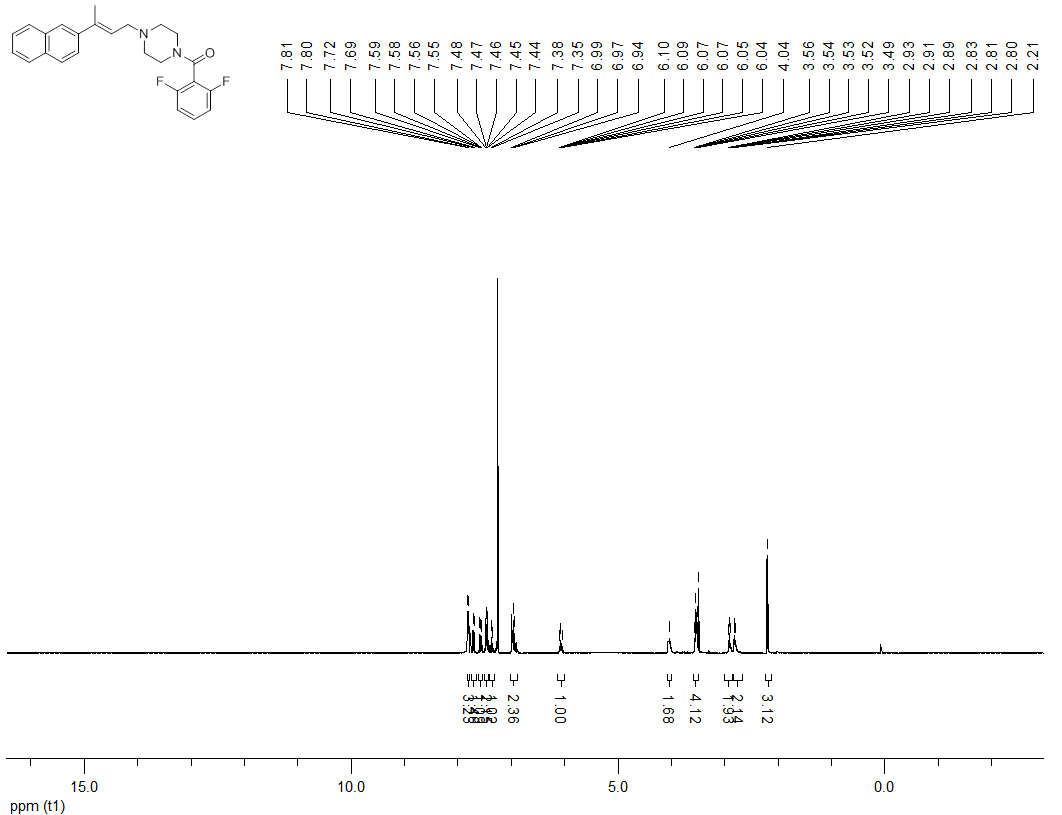
**

**^13^C NMR (100 MHz, CDCl_3_) 1-(2,6-difluorobenzoyl)-4-[(2*E*)-3-(naphthalen-2-yl)but-2-en-1-yl]piperazine (Gate 2156)**

**
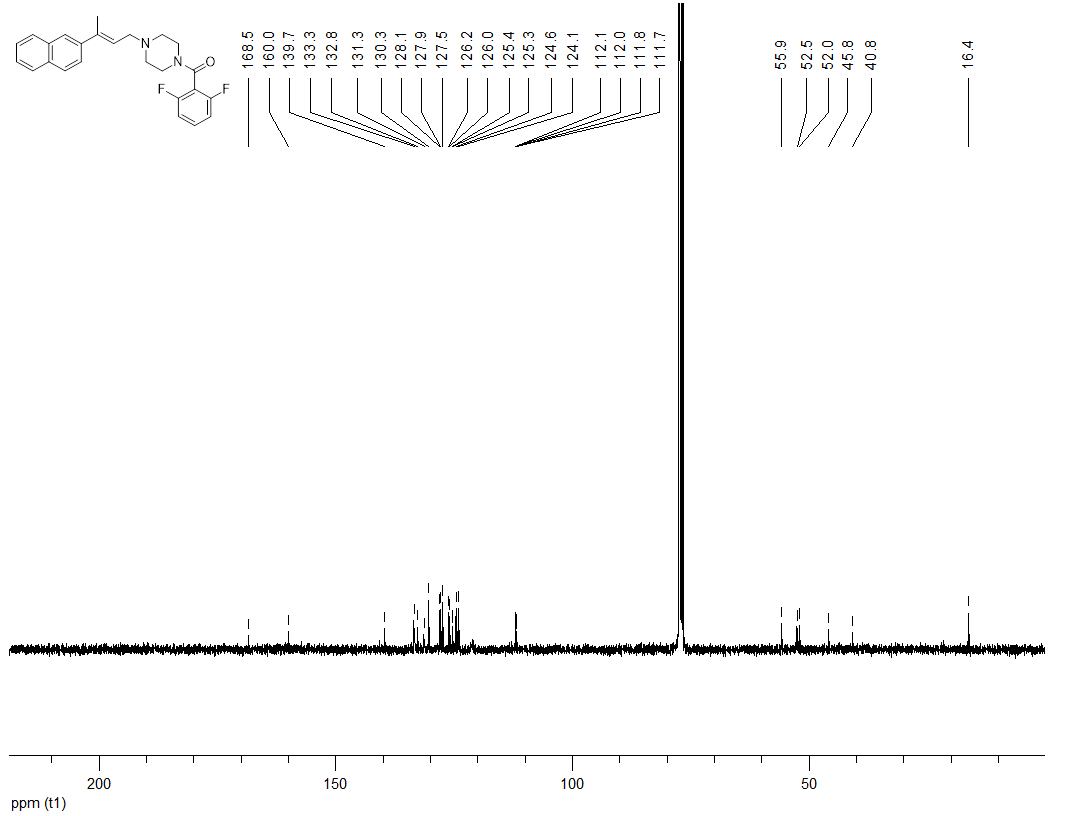
**

**^1^H and ^13^C NMR of some representative reductive amination products**

**^1^H NMR (300 MHz, CDCl_3_) 1-[(4-methylphenyl)methyl]-4-[(2*E*)-3-phenylbut-2-en-1-yl]piperazine (Gate2119)**


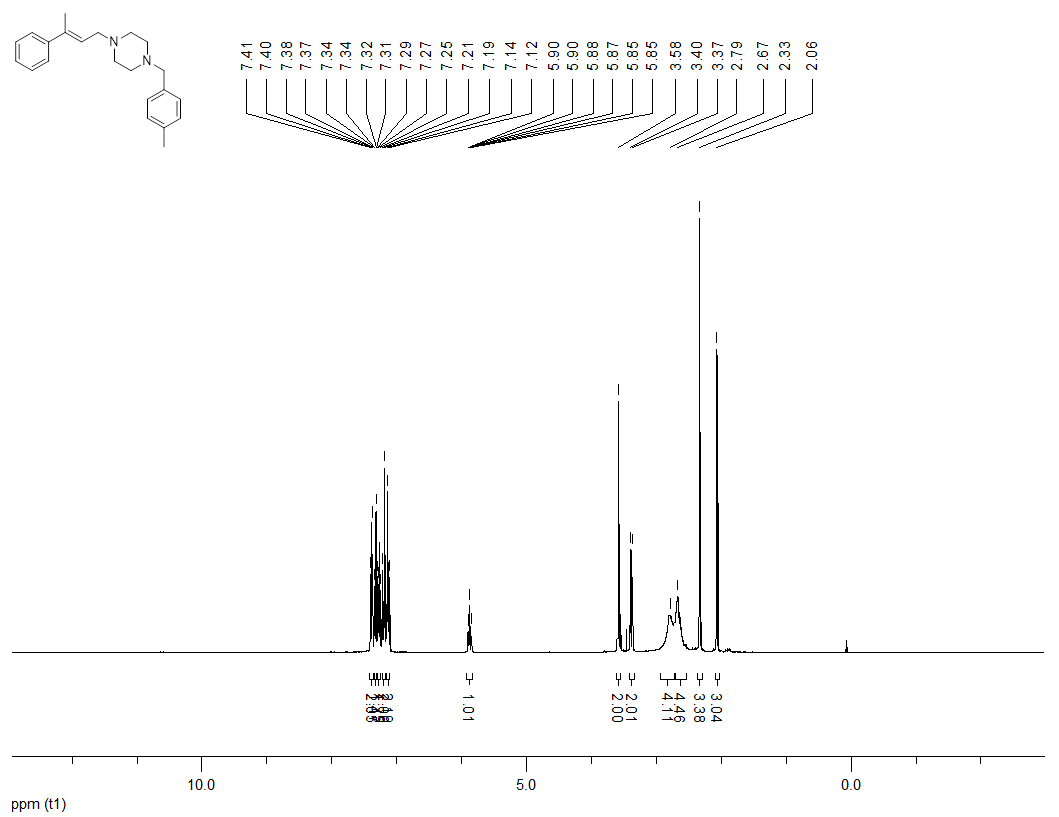


**^13^C NMR (100 MHz, CDCl_3_) 1-[(4-methylphenyl)methyl]-4-[(2*E*)-3-phenylbut-2-en-1-yl]piperazine (Gate2119)**

**
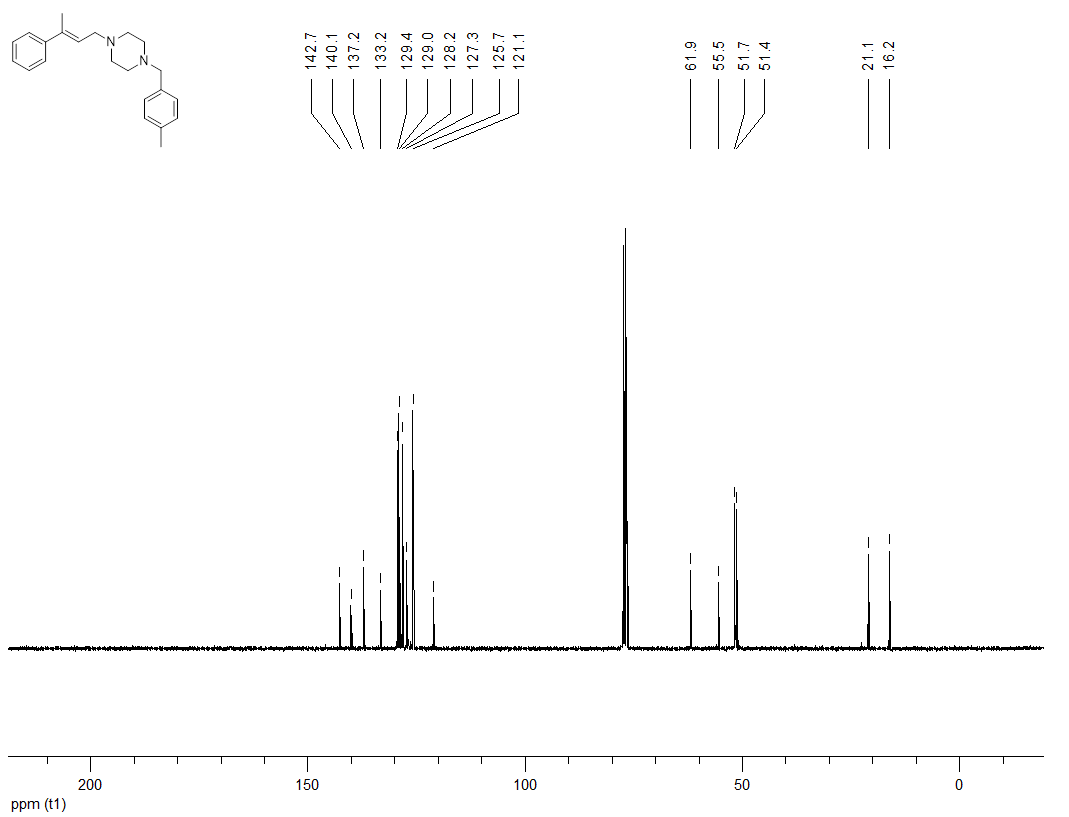
**

**^1^H NMR (300 MHz, CDCl_3_) 1-(cyclohexylmethyl)-4-[(2*E*)-3-(4-methoxyphenyl)but-2-en-1-yl]piperazine (Gate2123)**

**
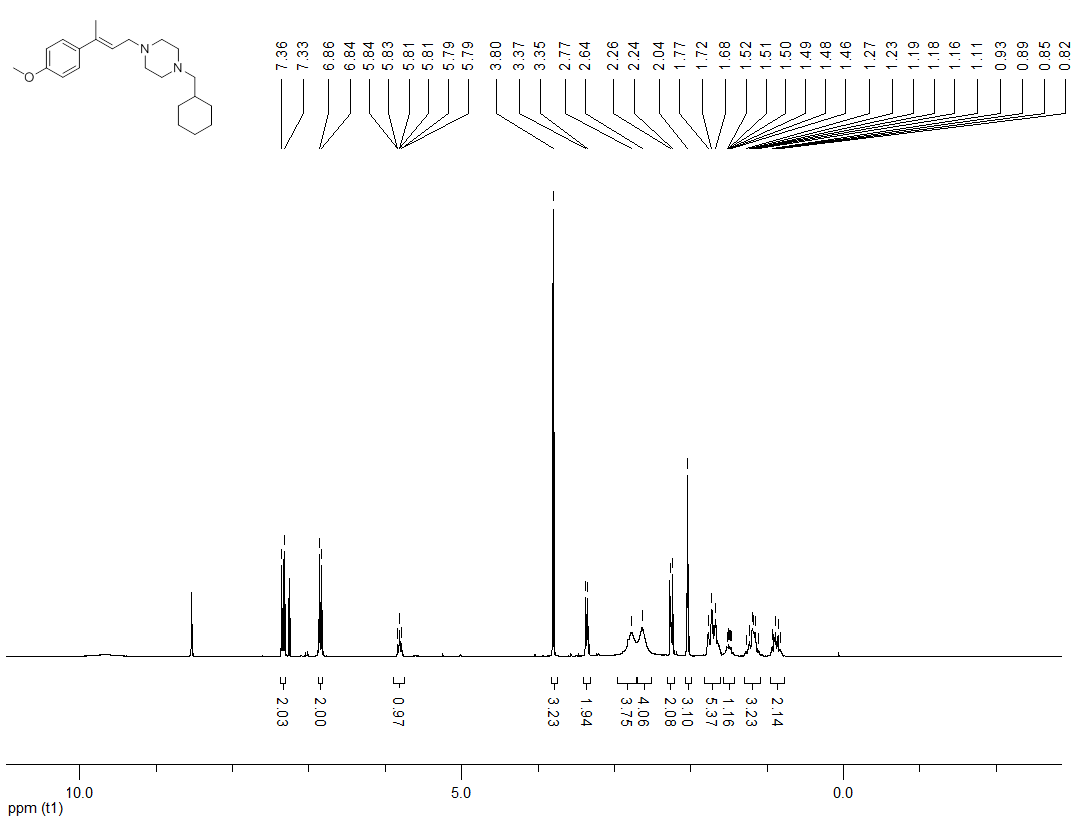
**

**^13^C NMR (100 MHz, CDCl_3_) 1-(cyclohexylmethyl)-4-[(2*E*)-3-(4-methoxyphenyl)but-2-en-1-yl]piperazine (Gate2123)**

**
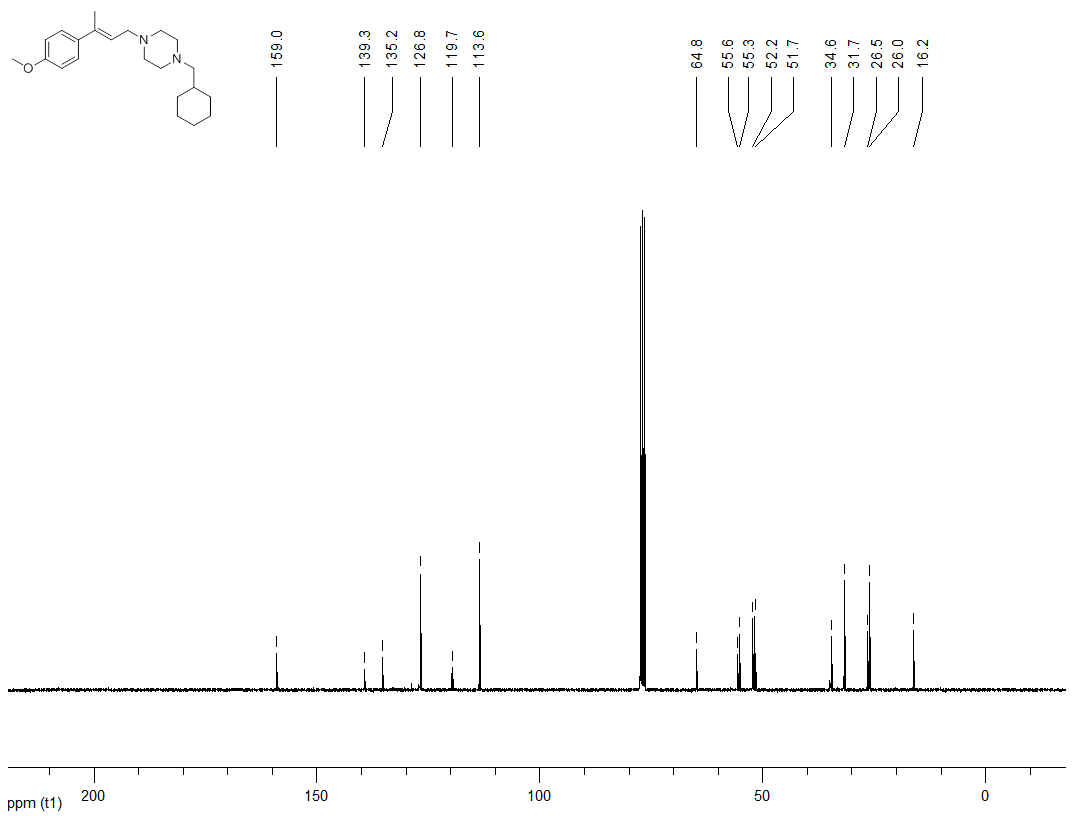
**

**^1^H NMR (300 MHz, CDCl_3_) 1-[(2*E*)-3-(4-methoxyphenyl)but-2-en-1-yl]-4-{[4-(trifluoromethyl)phenyl]methyl}piperazine (Gate 2124)**

**
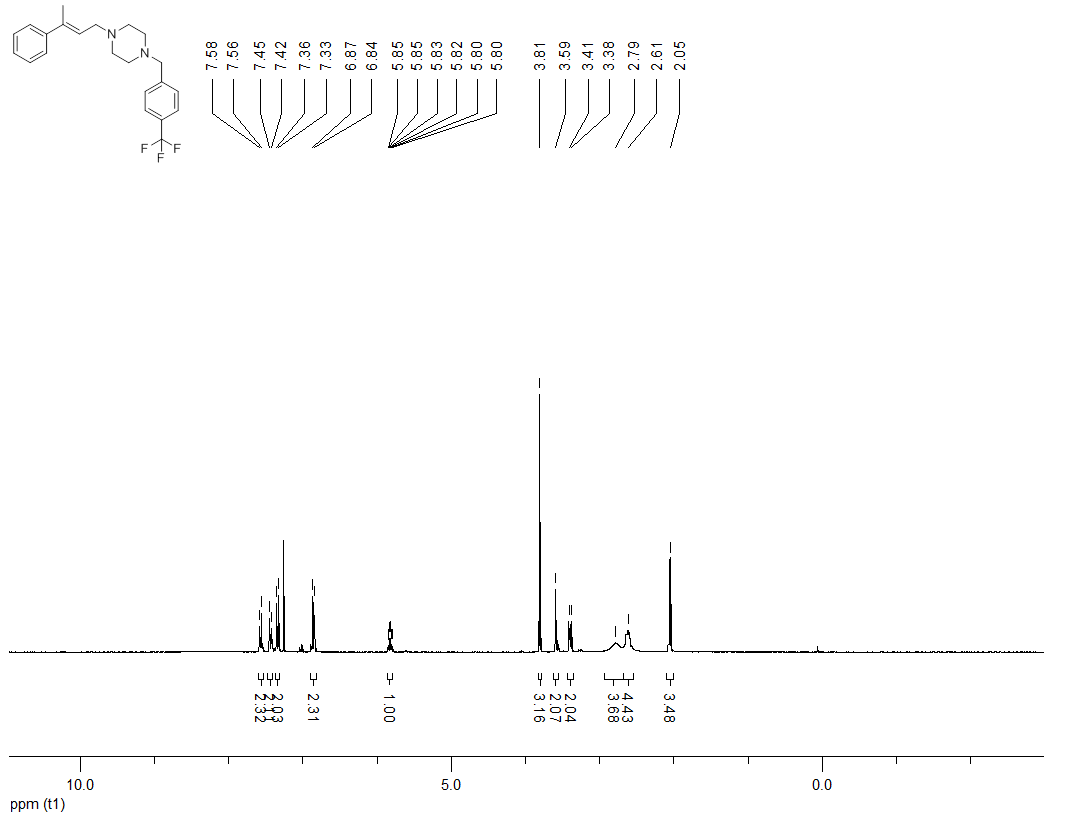
**

**^13^C NMR (100 MHz, CDCl_3_) 1-[(2*E*)-3-(4-methoxyphenyl)but-2-en-1-yl]-4-{[4-(trifluoromethyl)phenyl]methyl}piperazine (Gate 2124)**

**
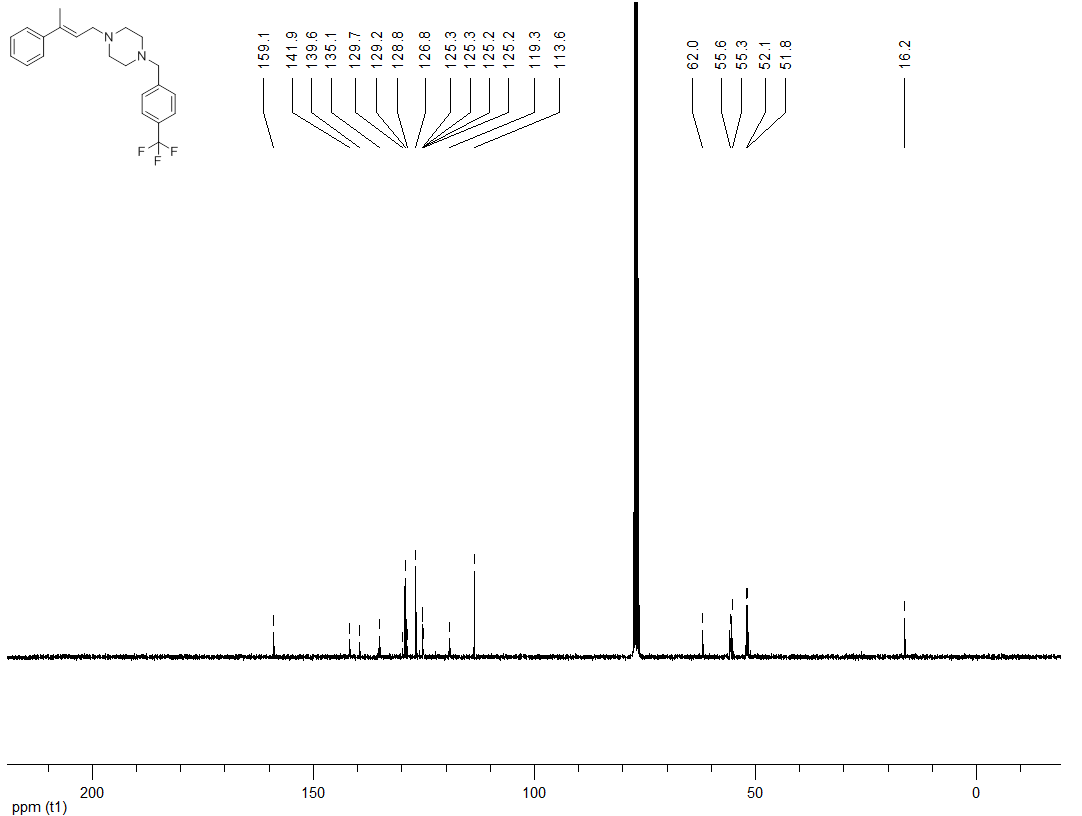
**

**^1^H NMR (300 MHz, CDCl_3_) 1-(cyclopentylmethyl)-4-[(2*E*)-3-(naphthalen-2-yl)but-2-en-1-yl]piperazine (Gate 2128)**


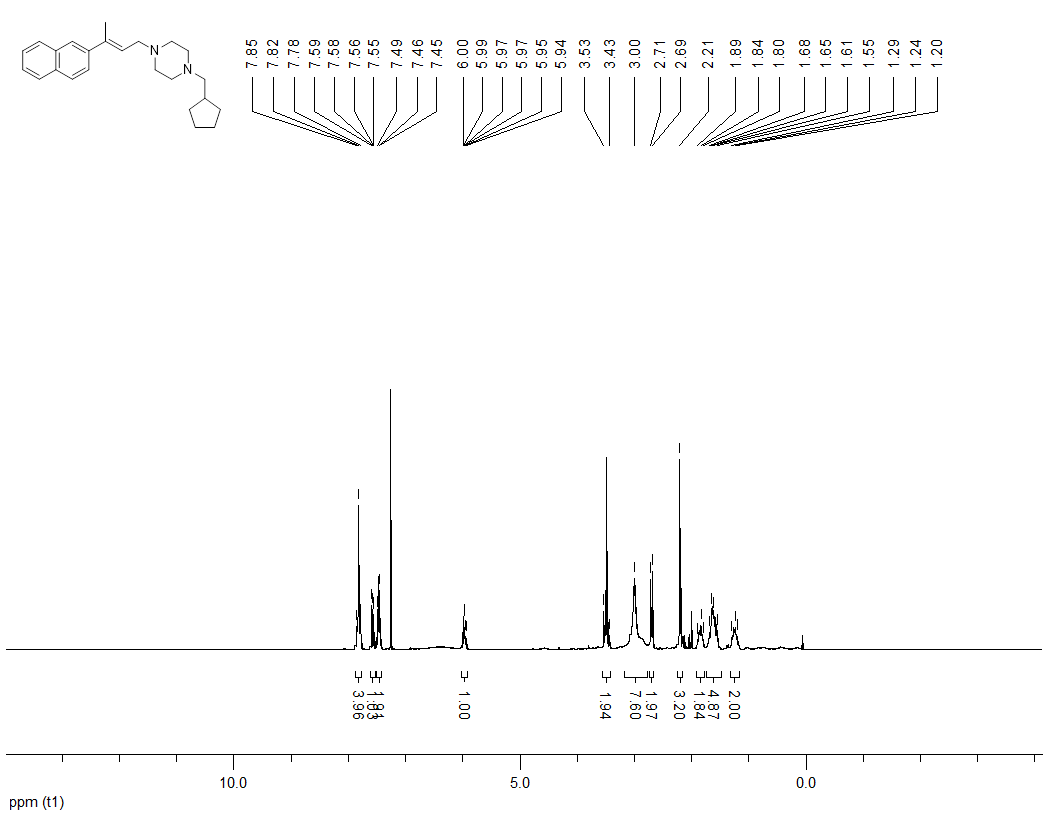


**^13^C NMR (100 MHz, CDCl_3_) 1-(cyclopentylmethyl)-4-[(2*E*)-3-(naphthalen-2-yl)but-2-en-1-yl]piperazine (Gate 2128)**

**
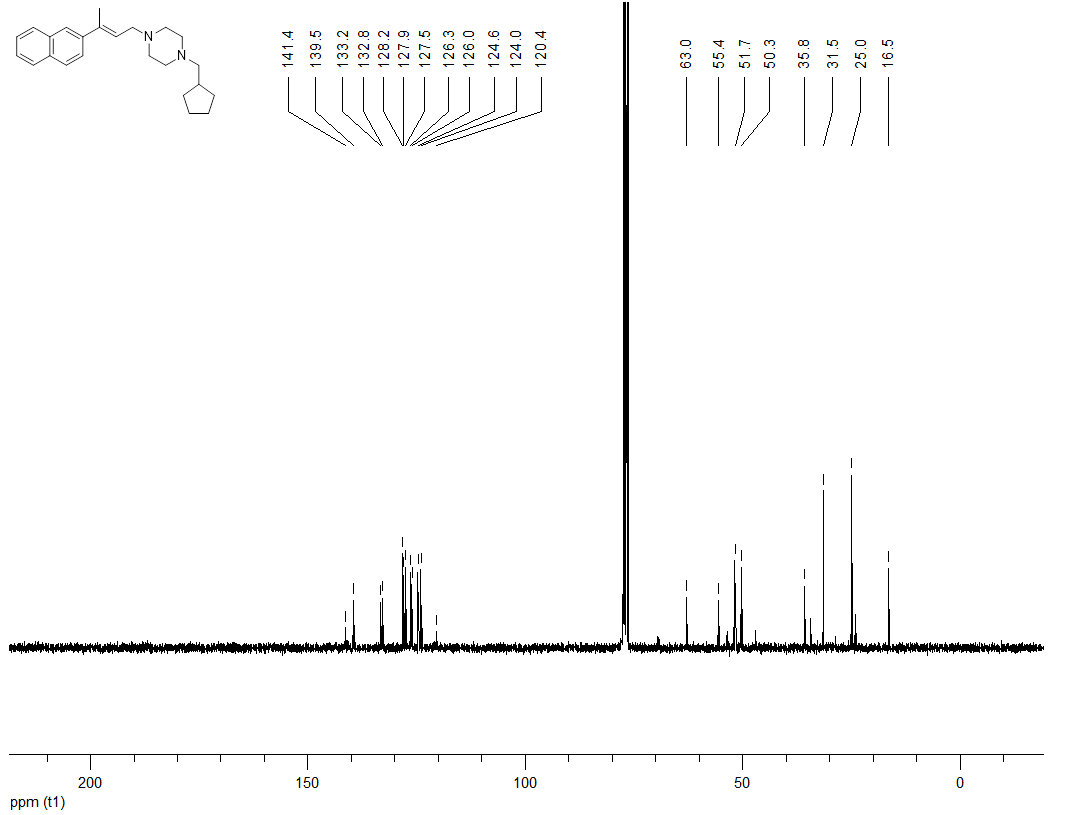
**

**^1^H NMR (300 MHz, CDCl_3_) 1-[(2,6-difluorophenyl)methyl]-4-[(2*E*)-3-(6-methoxynaphthalen-2-yl)but-2-en-1-yl]piperazine (Gate 2138)**


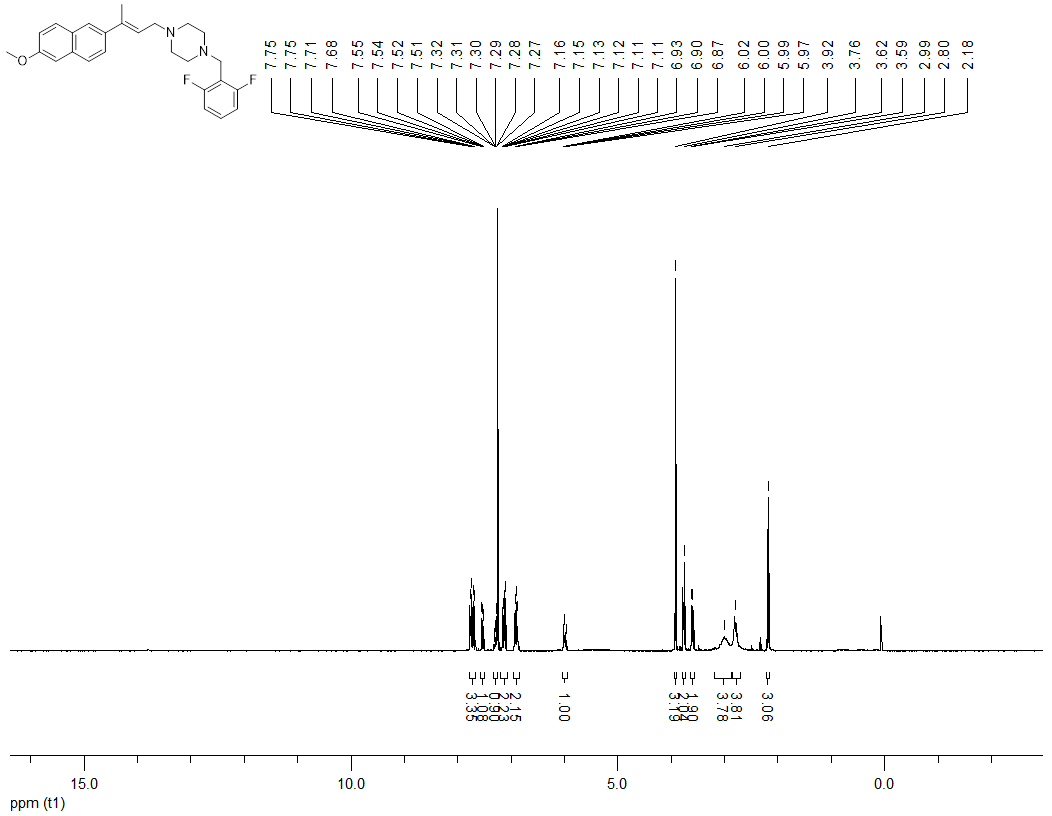


**^13^C NMR (100 MHz, CDCl_3_) 1-[(2,6-difluorophenyl)methyl]-4-[(2*E*)-3-(6-methoxynaphthalen-2-yl)but-2-en-1-yl]piperazine (Gate 2138)**

**
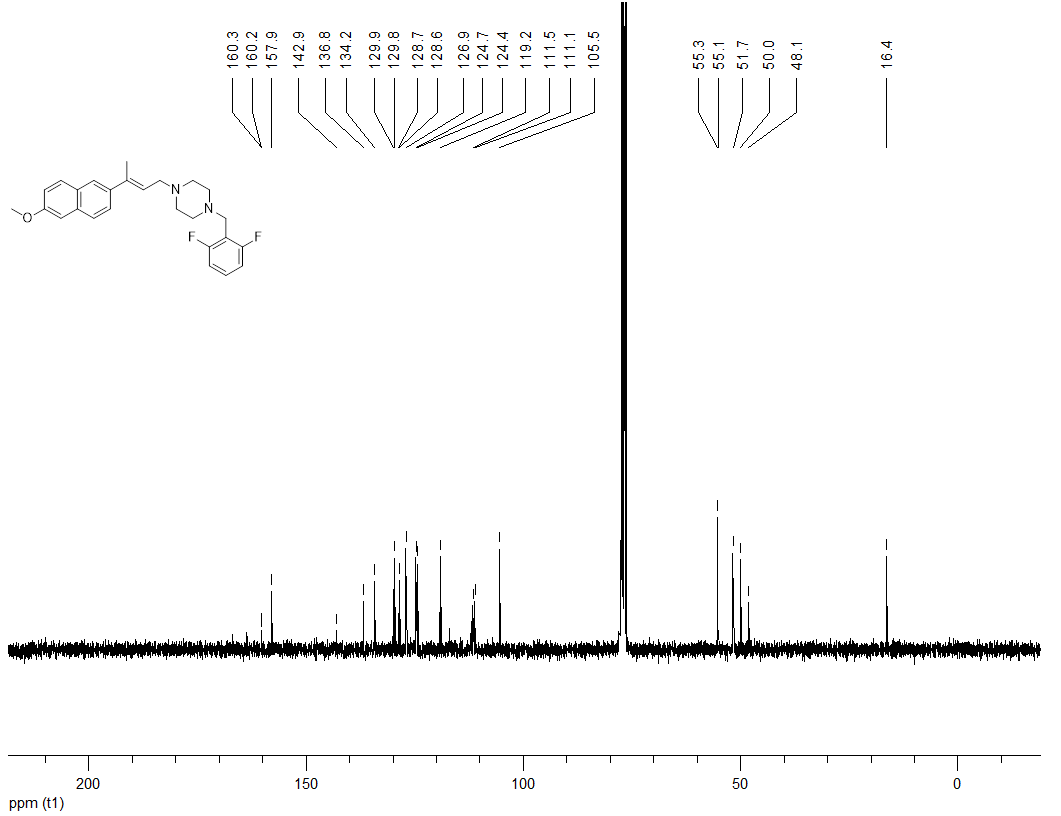
**

**^1^H and ^13^C NMR of some representative sulphonylation products**

**^1^H NMR (300 MHz, CDCl_3_) 1-[(2*E*)-3-phenylbut-2-en-1-yl]-4-[4-(trifluoromethyl)benzenesulfonyl]piperazine (Gate2166)**

**
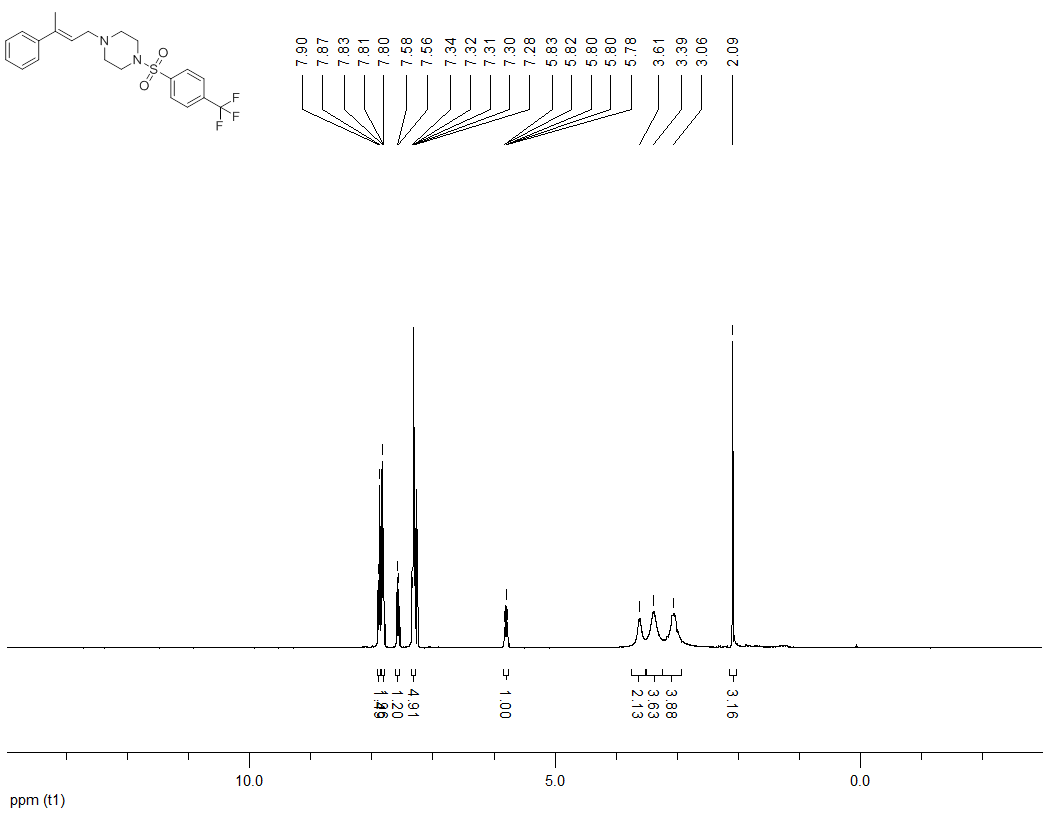
**

**^13^C NMR (100 MHz, CDCl_3_) 1-[(2*E*)-3-phenylbut-2-en-1-yl]-4-[4-(trifluoromethyl)benzenesulfonyl]piperazine (Gate2166)**

**
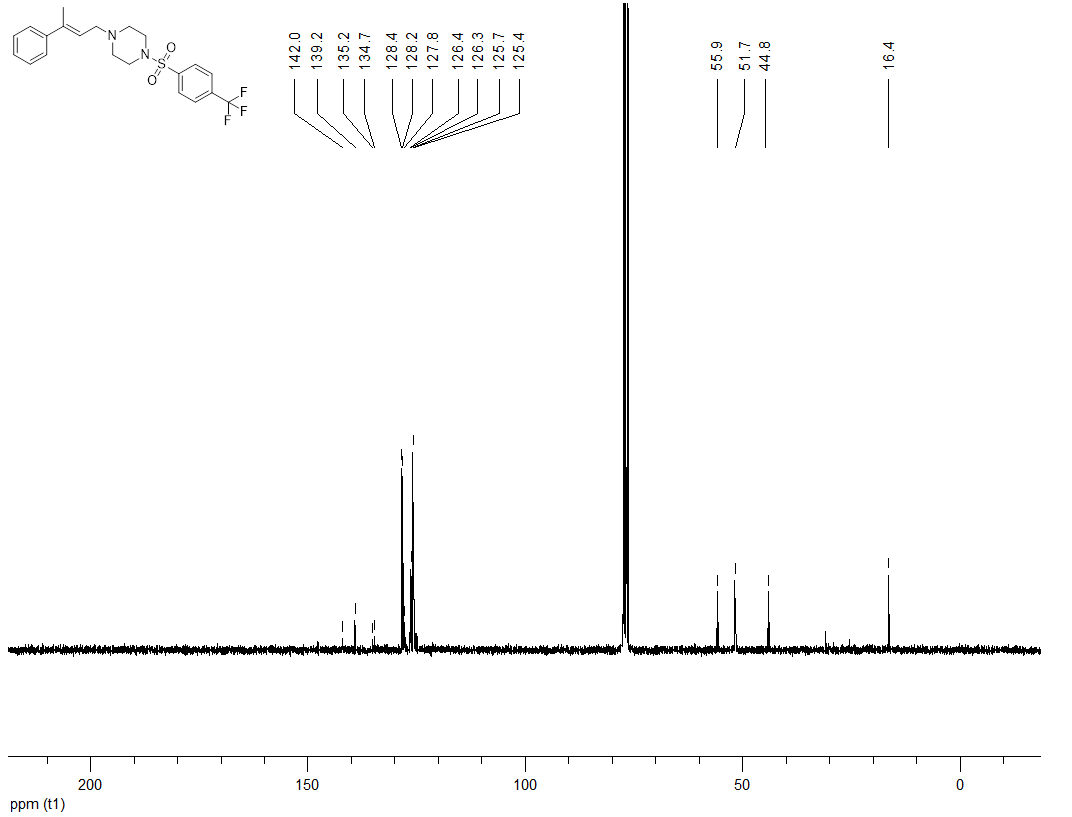
**

**^1^H NMR (300 MHz, CDCl_3_) 1-(4-methylbenzenesulfonyl)-4-[(2*E*)-3-phenylbut-2-en-1-yl]piperazine (Gate 2167)**

**
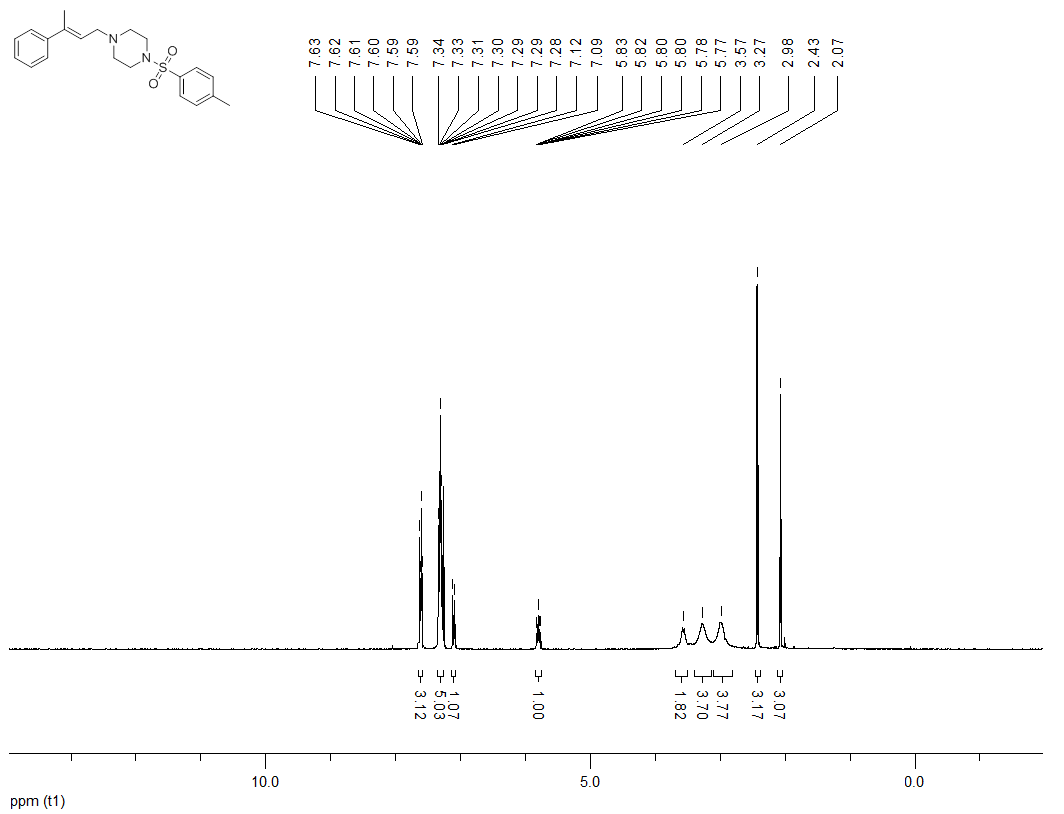
**

**^13^C NMR (100 MHz, CDCl_3_) 1-(4-methylbenzenesulfonyl)-4-[(2*E*)-3-phenylbut-2-en-1-yl]piperazine (Gate 2167)**

**
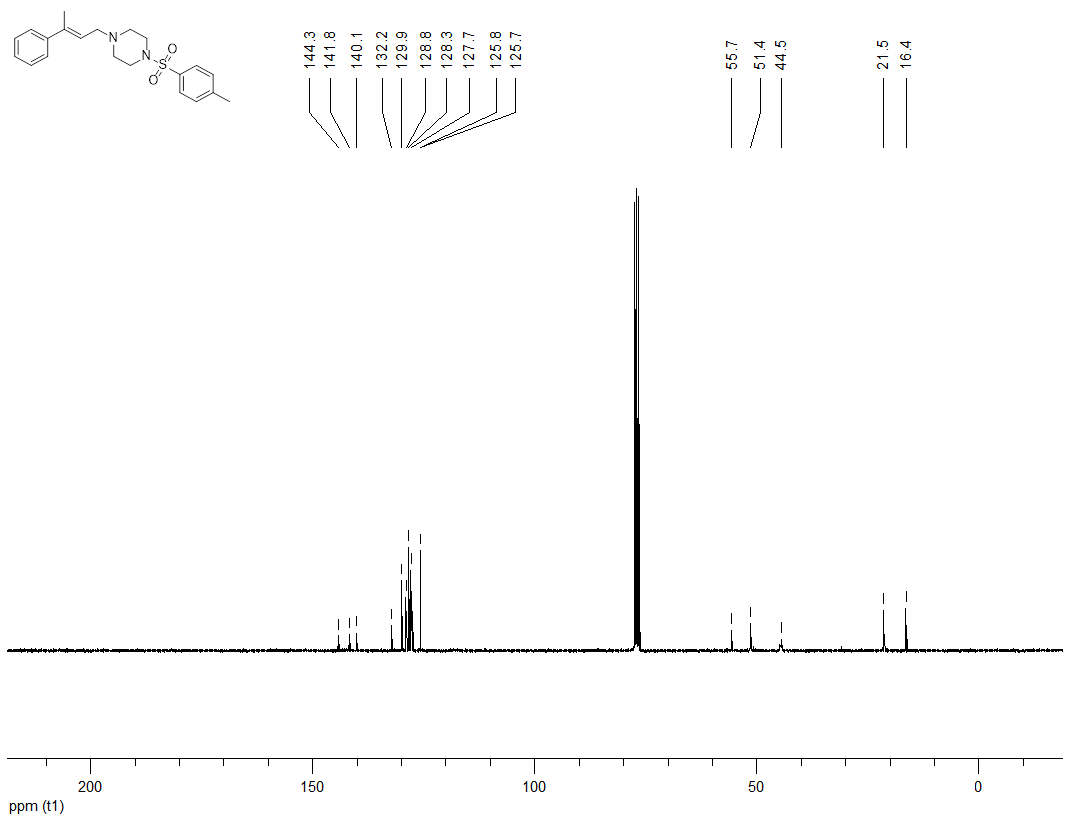
**

**^1^H NMR (300 MHz, CDCl_3_) 1-(cyclopentanesulfonyl)-4-[(2*E*)-3-(4-methoxyphenyl)but-2-en-1-yl]piperazine (Gate2170)**

**
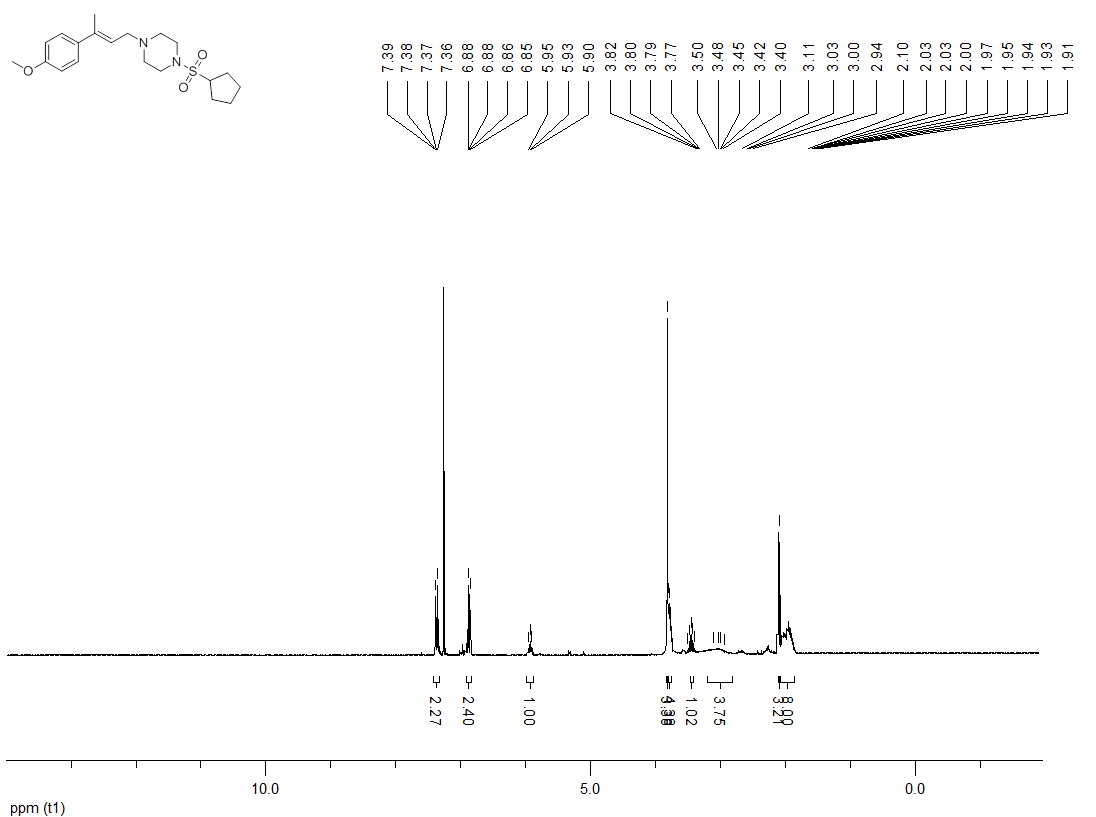
**

**^13^C NMR (100 MHz, CDCl_3_) 1-(cyclopentanesulfonyl)-4-[(2*E*)-3-(4-methoxyphenyl)but-2-en-1-yl]piperazine (Gate2170)**

**
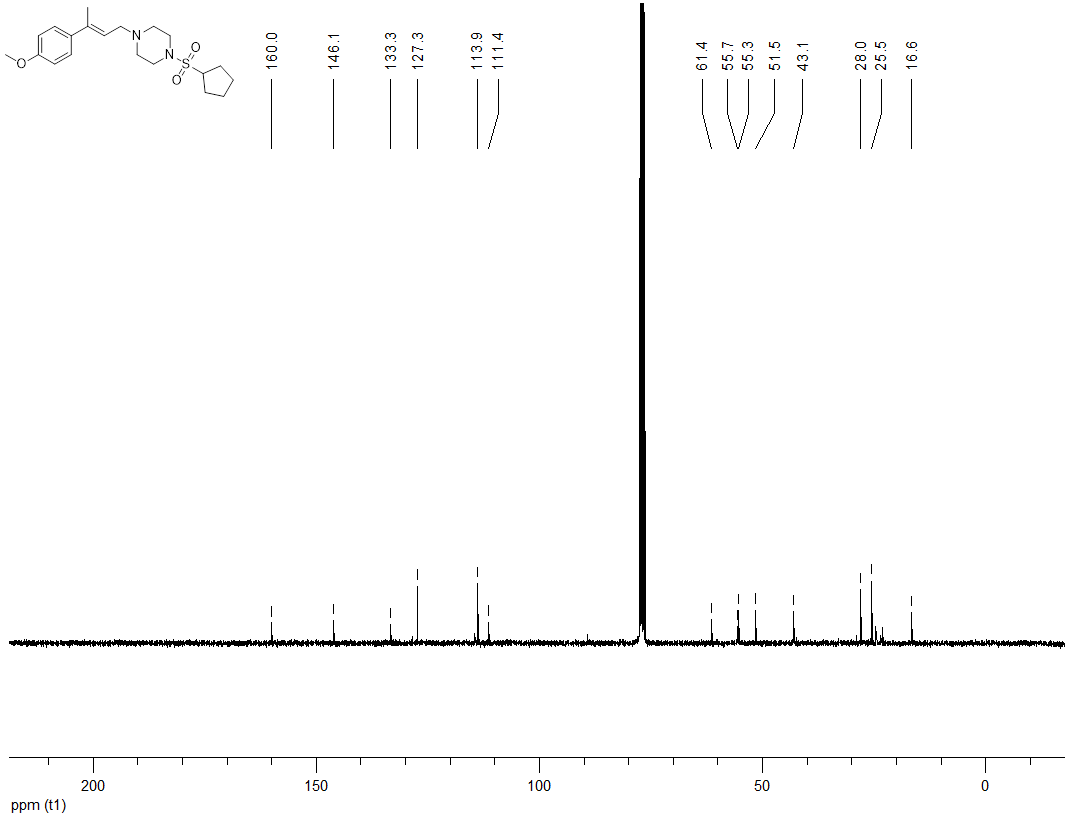
**

**^1^H NMR (300 MHz, CDCl_3_) 1-(cyclopentanesulfonyl)-4-[(2*E*)-3-(naphthalen-2-yl)but-2-en-1-yl]piperazine (Gate 2176)**

**
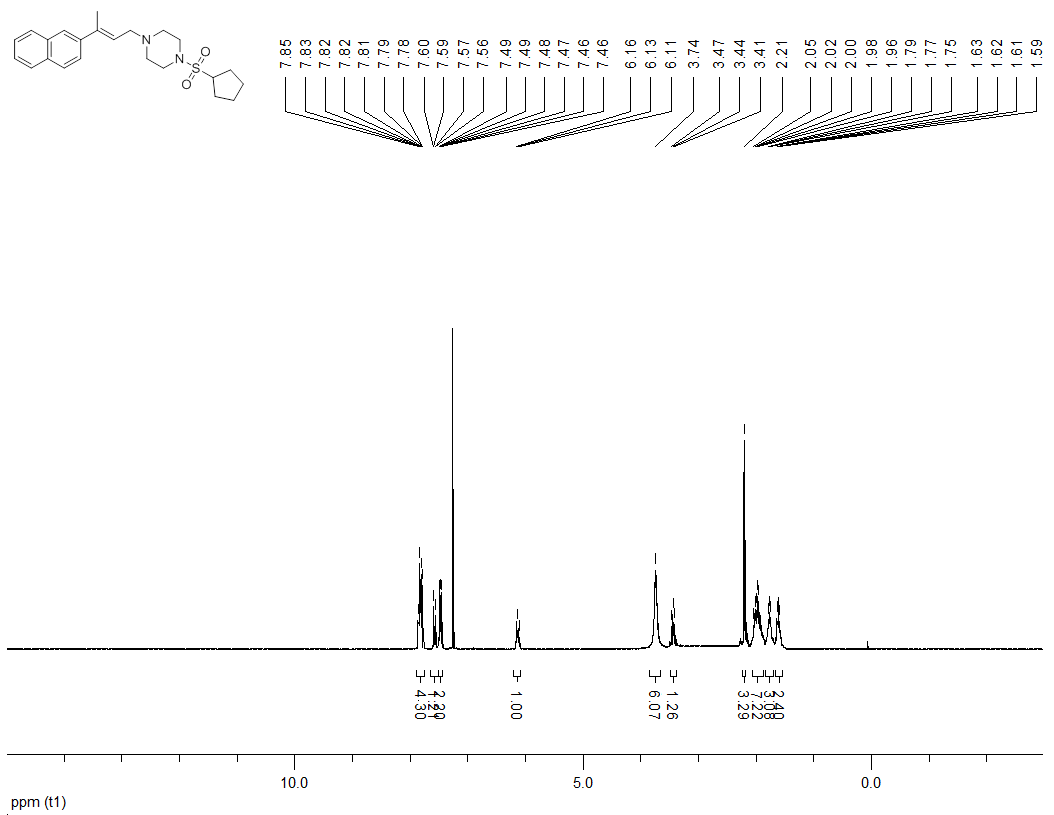
**

**^13^C NMR (100 MHz, CDCl_3_) 1-(cyclopentanesulfonyl)-4-[(2*E*)-3-(naphthalen-2-yl)but-2-en-1-yl]piperazine (Gate 2176)**

**
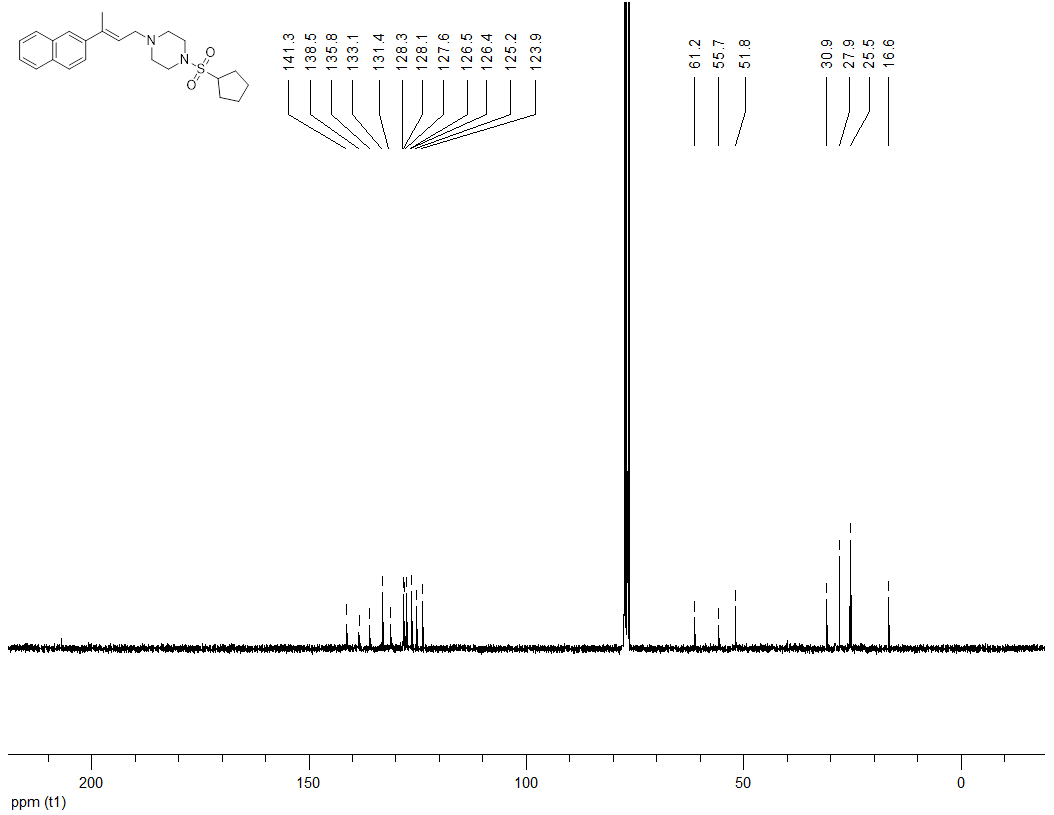
**

**^1^H and ^13^C NMR of some representative urea formation products**

**^1^H NMR (300 MHz, CDCl_3_) *N*-(4-methylphenyl)-4-[(2*E*)-3-phenylbut-2-en-1-yl]piperazine-1-carboxamide (Gate 2191)**

**
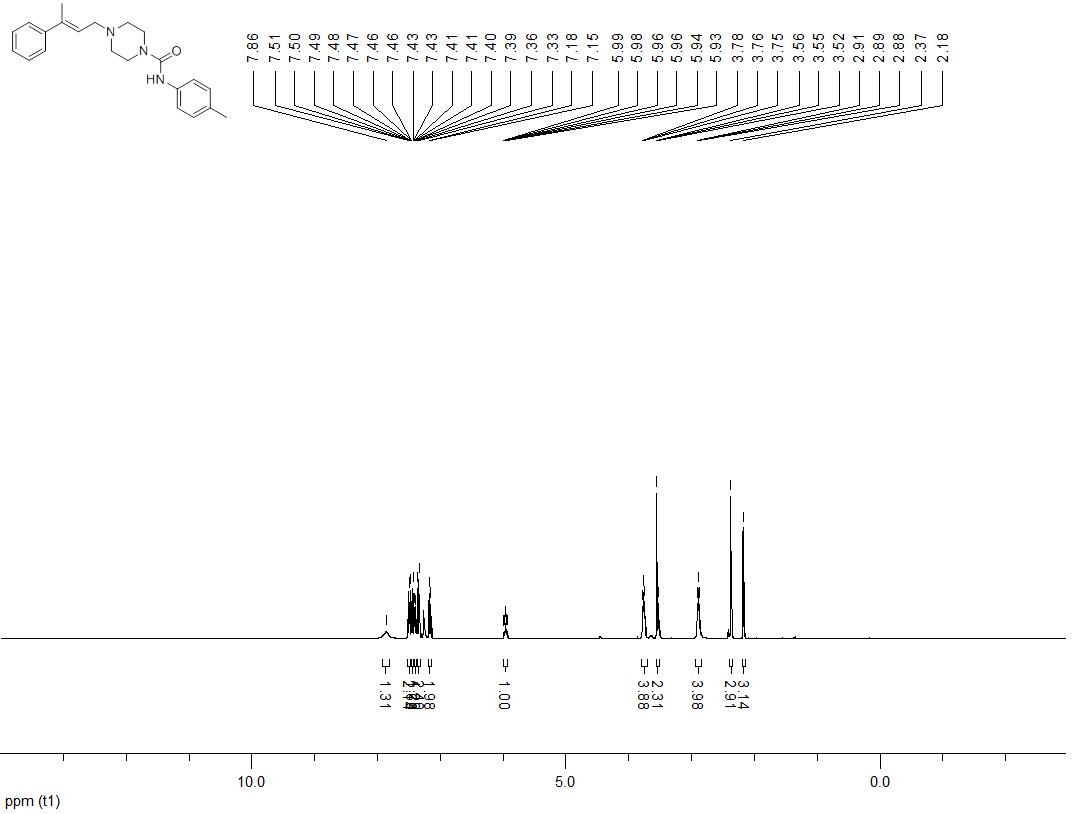
**

**^13^C NMR (100 MHz, CDCl_3_) *N*-(4-methylphenyl)-4-[(2*E*)-3-phenylbut-2-en-1-yl]piperazine-1-carboxamide (Gate 2191)**

**
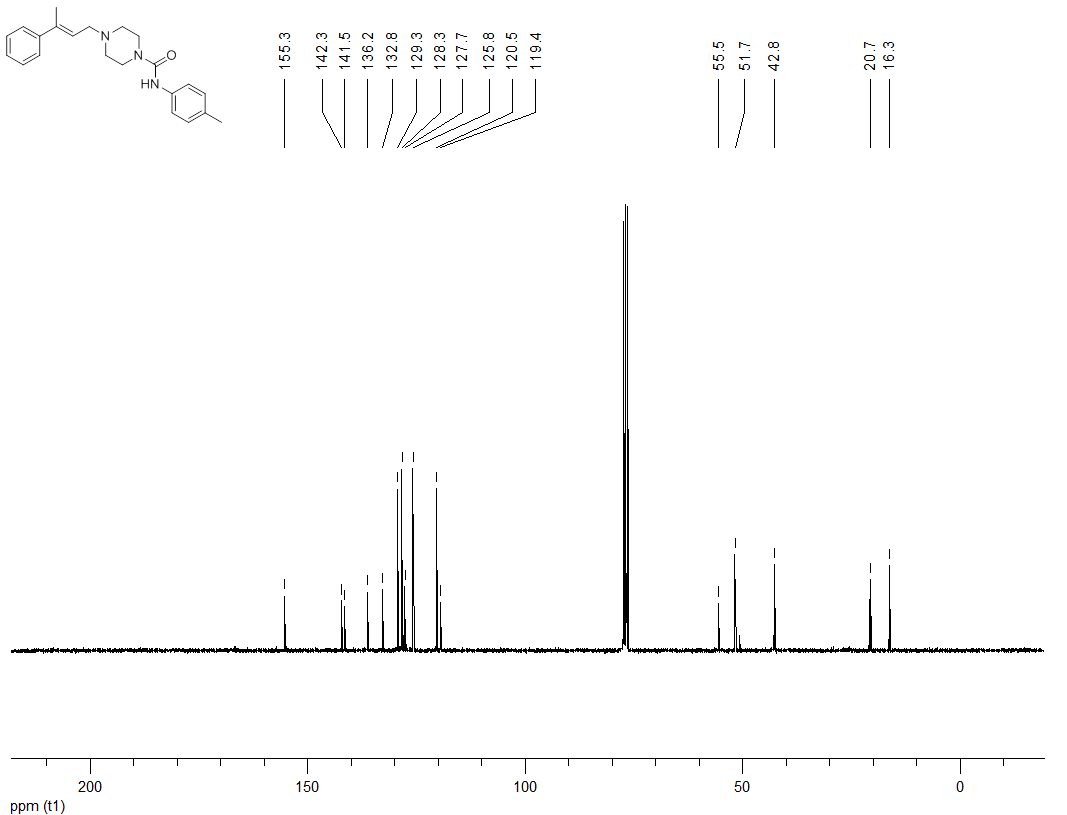
**

**^1^H NMR (300 MHz, CDCl_3_) *N*-cyclohexyl-4-[(2*E*)-3-(4-methoxyphenyl)but-2-en-1-yl]piperazine-1-carboxamide (Gate 2195)**

**
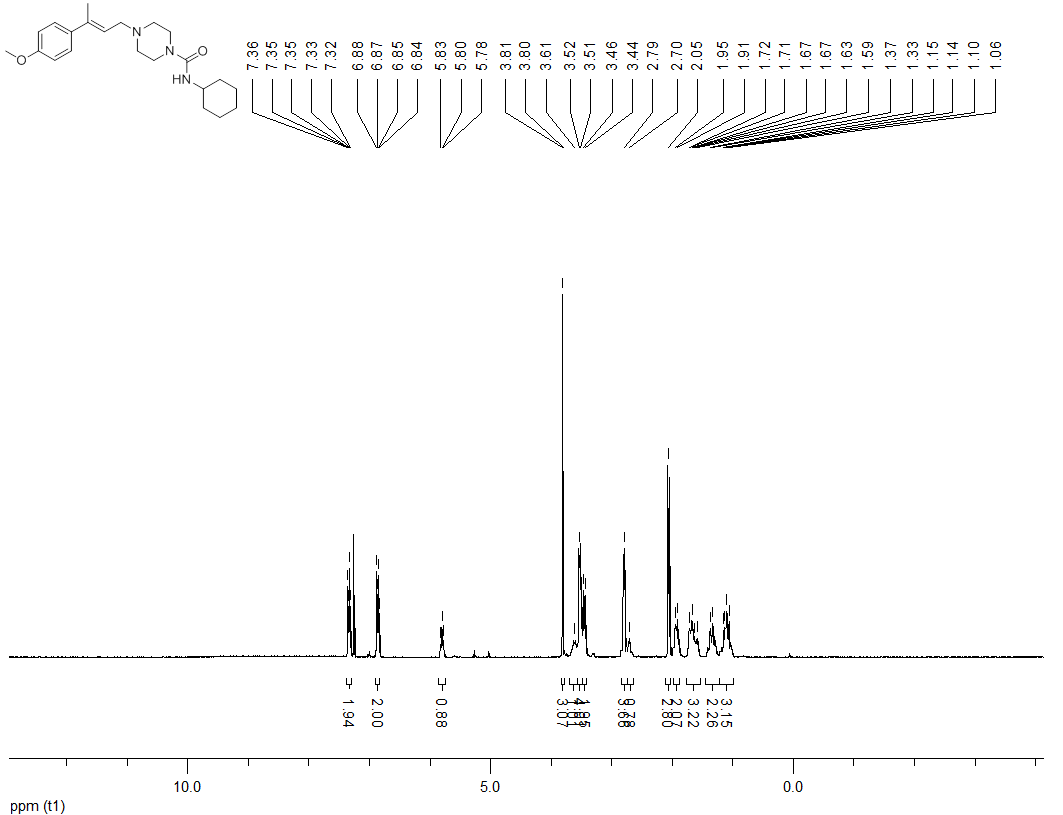
**

**^13^C NMR (100 MHz, CDCl_3_) *N*-cyclohexyl-4-[(2*E*)-3-(4-methoxyphenyl)but-2-en-1-yl]piperazine-1-carboxamide (Gate 2195)**

**
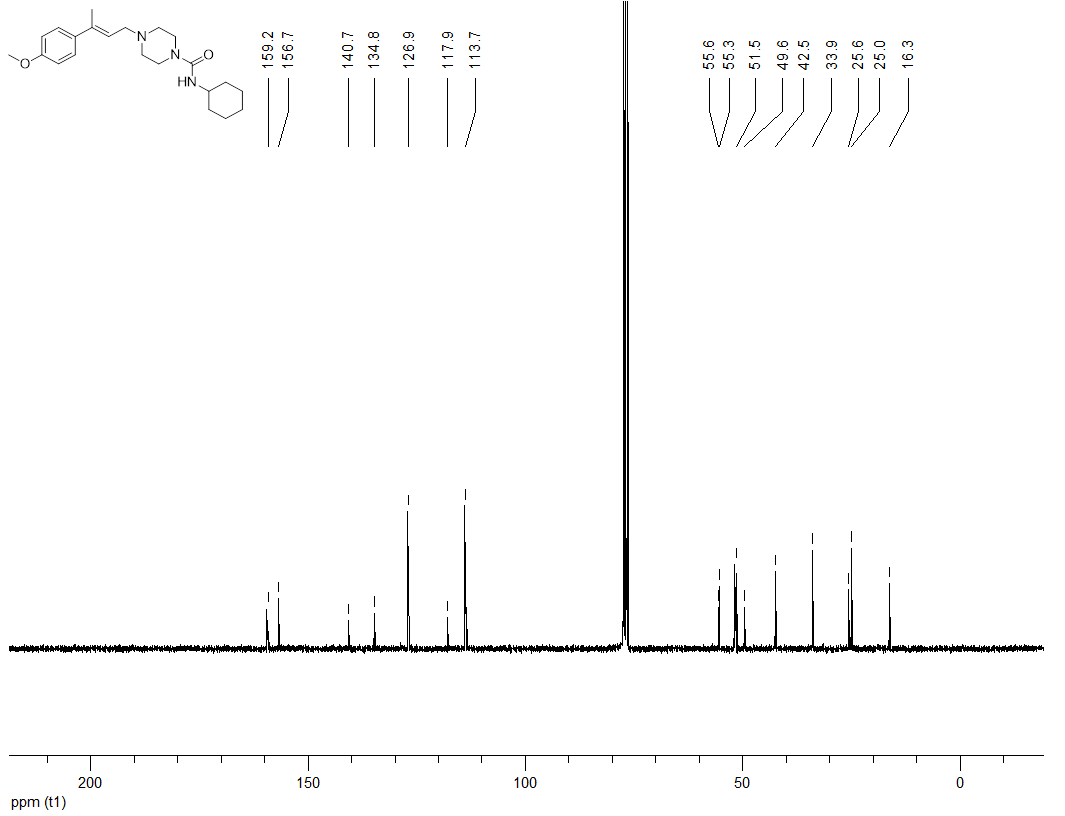
**

**^1^H NMR (300 MHz, CDCl_3_) *N*-cyclopentyl-4-[(2*E*)-3-(naphthalen-2-yl)but-2-en-1-yl]piperazine-1-carboxamide (Gate 2200)**

**
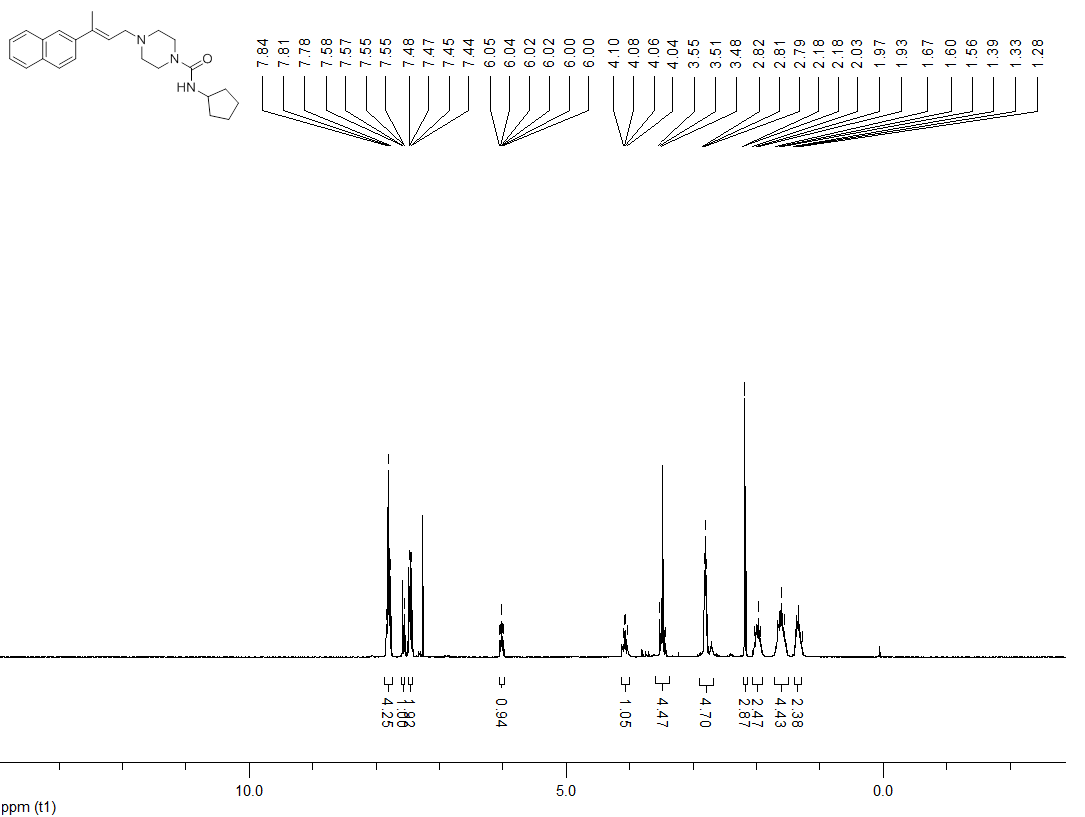
**

**^13^C NMR (100 MHz, CDCl_3_) *N*-cyclopentyl-4-[(2*E*)-3-(naphthalen-2-yl)but-2-en-1-yl]piperazine-1-carboxamide (Gate 2200)**

**
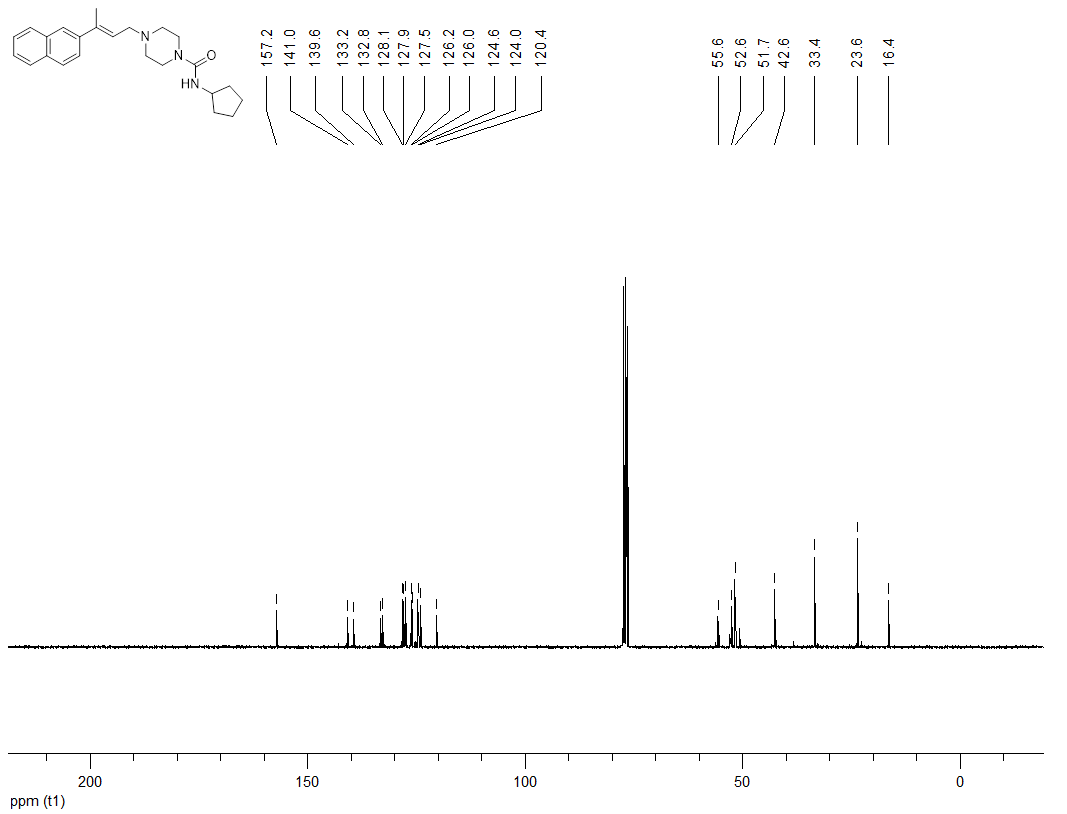
**

**^1^H NMR (300 MHz, CDCl_3_) *N*-cyclohexyl-4-[(2*E*)-3-(naphthalen-2-yl)but-2-en-1-yl]piperazine-1-carboxamide (Gate 2201)**

**
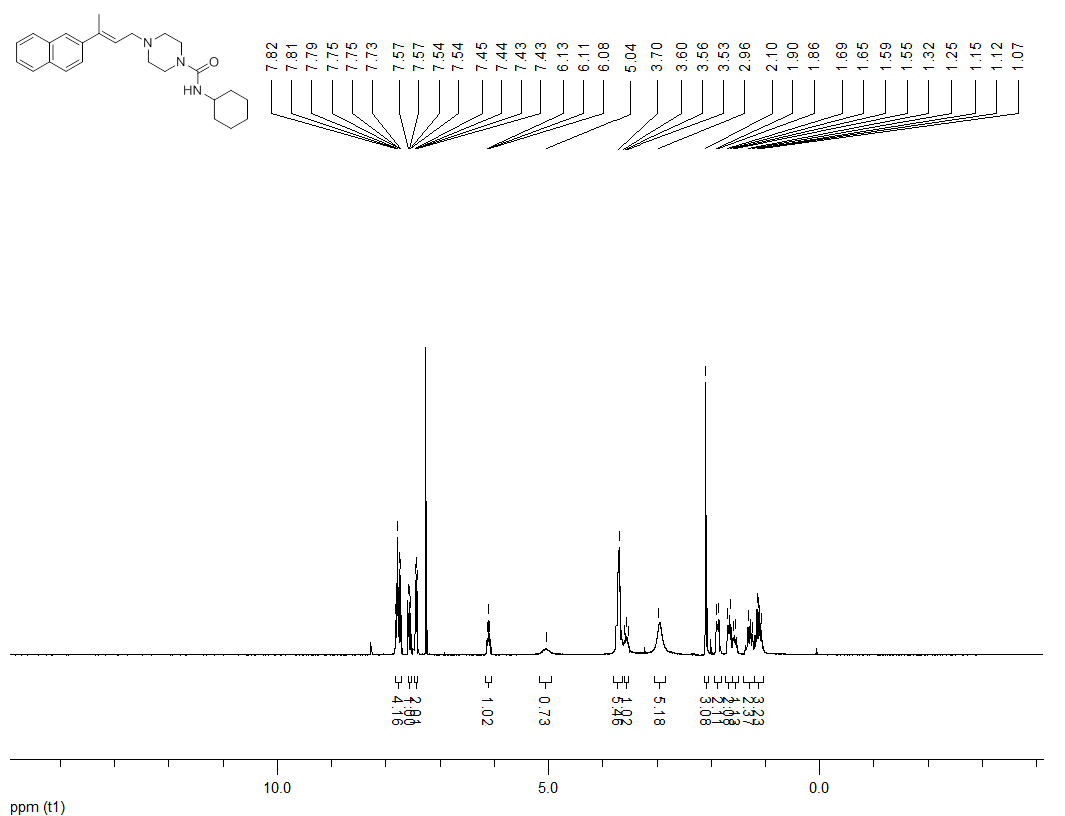
**

**^13^C NMR (100 MHz, CDCl_3_) *N*-cyclohexyl-4-[(2*E*)-3-(naphthalen-2-yl)but-2-en-1-yl]piperazine-1-carboxamide (Gate 2201)**

**
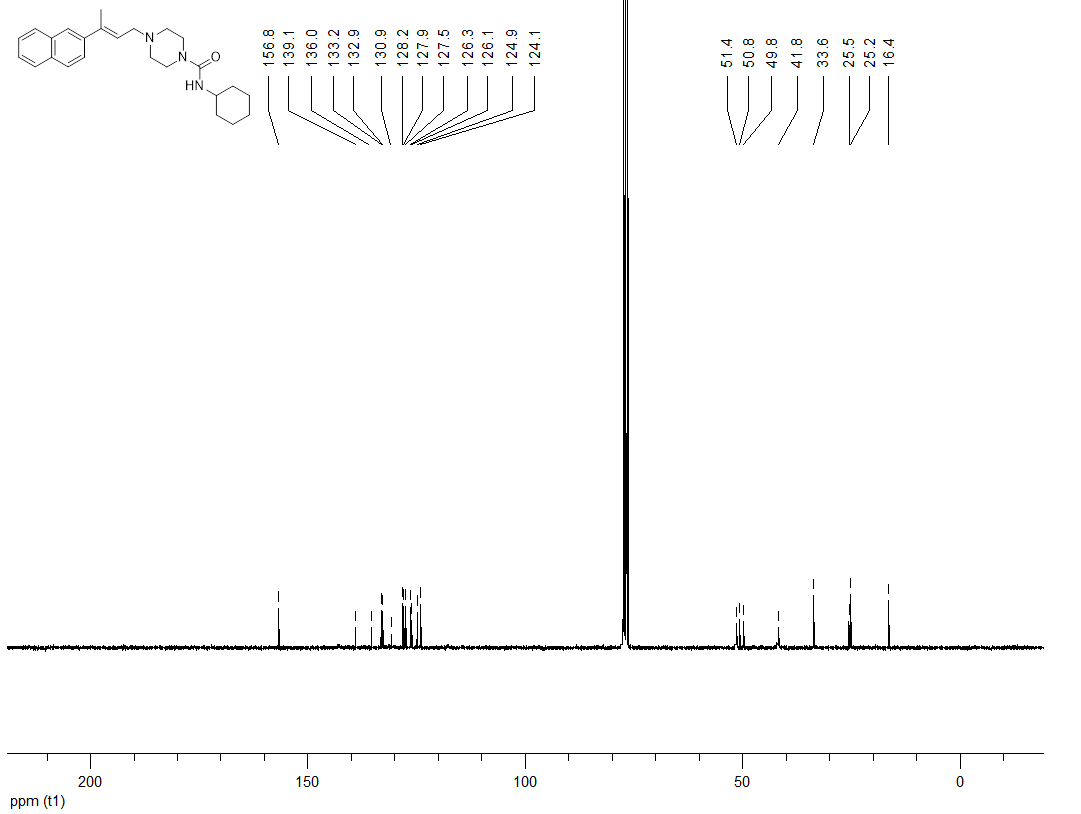
**

**^1^H NMR (300 MHz, CDCl_3_) *N*-(2,6-difluorophenyl)-4-[(2*E*)-3-(6-methoxynaphthalen-2-yl)but-2-en-1-yl]piperazine-1-carboxamide (Gate 2210)**

**
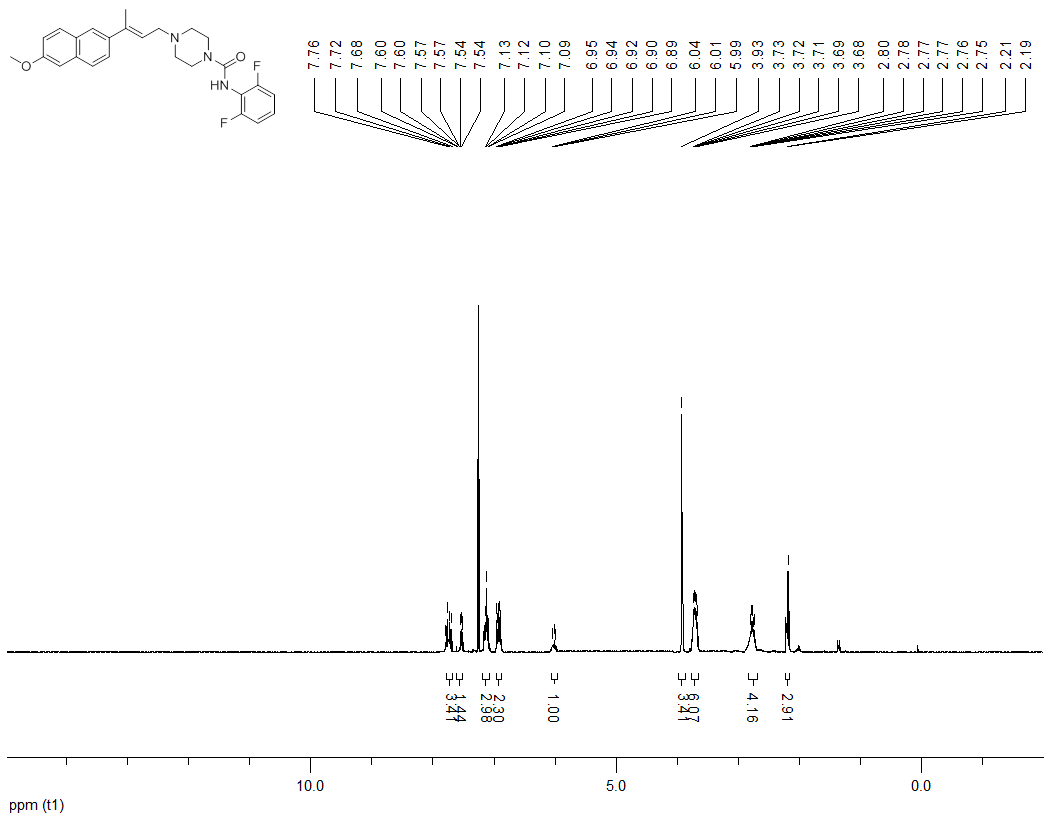
**

**^13^C NMR (100 MHz, CDCl_3_) *N*-(2,6-difluorophenyl)-4-[(2*E*)-3-(6-methoxynaphthalen-2-yl)but-2-en-1-yl]piperazine-1-carboxamide (Gate 2210)**

**
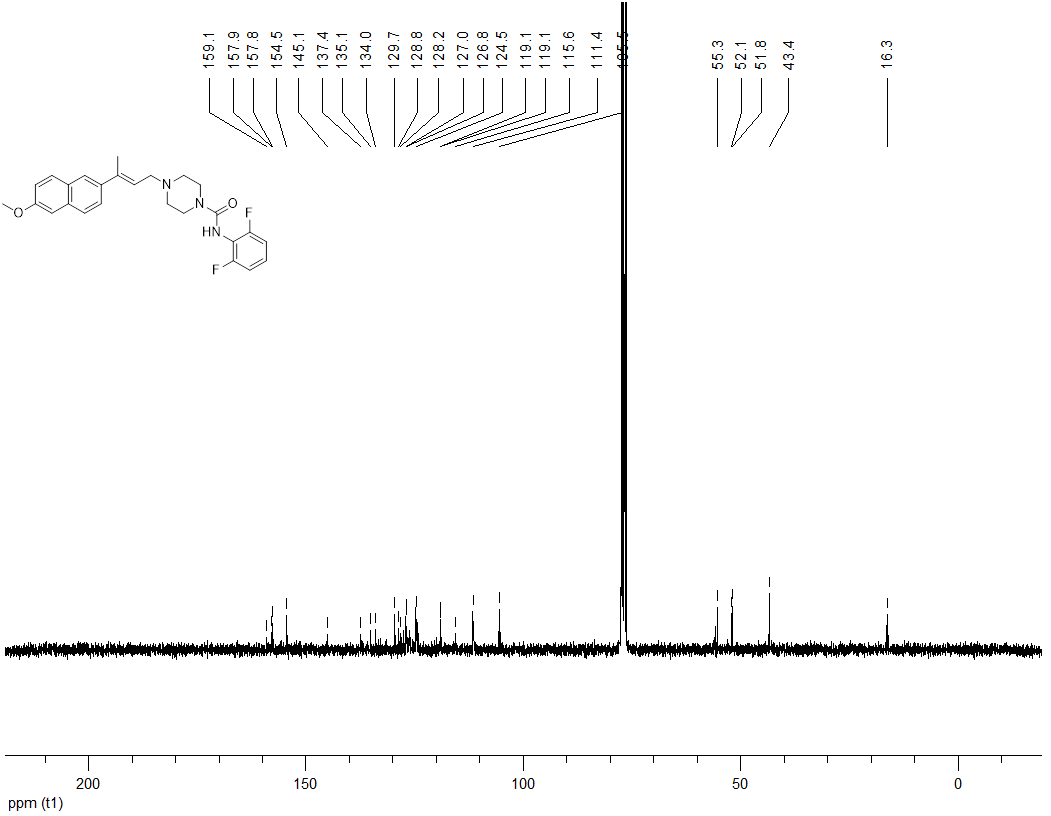
**
